# Cyclopamine analogs bearing exocyclic methylenes are highly potent and acid-stable inhibitors of hedgehog signaling

**DOI:** 10.3762/bjoc.9.267

**Published:** 2013-10-31

**Authors:** Johann Moschner, Anna Chentsova, Nicole Eilert, Irene Rovardi, Philipp Heretsch, Athanassios Giannis

**Affiliations:** 1 Universität Leipzig, Institut für Organische Chemie, Johannisallee 29, D-04103 Leipzig, Germany; 2 Rice University, Department of Chemistry, BioScience Research Collaborative, 6500 Main St., Houston, Texas 77030, USA

**Keywords:** cyclopamine, hedgehog signaling pathway, natural products, steroidal alkaloids, structure–activity relationship

## Abstract

The chemical synthesis and biological evaluation of new cyclopamine analogs bearing exocyclic methylenes in different positions is described. Bis-*exo*-cyclopamine **6** was identified as a potent inhibitor of the Gli1-dependent luciferase expression in Shh-LIGHTII cells. An extension of this study to F-ring-modified structures shows the necessity of a rigidly positioned nitrogen atom for bioactivity as well as the presence of the C21 methyl group for acid stability and bioactivity.

## Introduction

Hedgehog signaling is involved in embryonic development and plays an important role in the maintenance of stem cells, tissue repair and regeneration in adult organisms [1–4]. The erroneous activation of hedgehog signaling is tightly associated with the occurrence of basal cell carcinoma and medulloblastoma [5]. In addition, several other tumors are co-dependent on hedgehog signaling, examples for this type are cancers of the skin [6–7], brain [8–9], lung [10], pancreas [11], breast [12], prostate [13–14], colon [15], rhabdomyosarcoma [16], lymphoma [17–19], multiple myeloma [17,20], and chronic myeloic leukemia [21–23]. More recently, other diseases like diabetes [24–25], neurodegenerative disorders [26], and trisomy [27–28], have been linked with hedgehog signaling.

**Figure 1:**
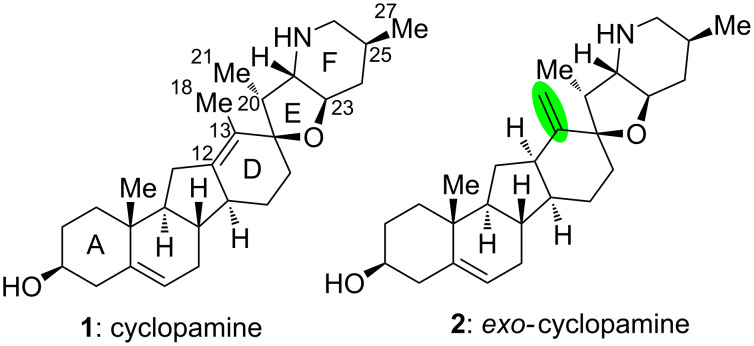
Structures of cyclopamine and *exo*-cyclopamine.

Cyclopamine (**1**, see Figure 1) was the first inhibitor of the hedgehog signaling pathway to be identified. As a highly selective inhibitor of the transmembrane protein Smoothened (Smo), an integral component of hedgehog signaling, it presents an attractive target for medicinal and pharmaceutical research [29–30]. Unfortunately, its direct development into a drug is hampered by its low metabolic stability (decomposition at pH < 3) [31] and rather moderate potency (IC_50_ ~ 5 µM).

We previously reported the first chemical synthesis of cyclopamine (**1**) starting from dehydroepiandrosterone and utilizing the C–H-functionalization logic and a biomimetic skeleton rearrangement [32–33]. Furthermore, quantum mechanical calculations guided our design and synthesis of *exo*-cyclopamine (**2**, see Figure 1), a ten-fold more potent and acid-stable analog with an *exo*-methylene unit at C13–C18 [34]. Herein, we describe a comprehensive study of cyclopamine analogs bearing *exo*-methylene units in different positions and extent this rational to F-ring-modified structures.

## Results and Discussion

Our synthetic approach to the analogs described here started from previously reported azide **3** which was converted in six steps [32] to the protected bis-*exo*-methylene compound **4**. Carefully chosen conditions for the deprotection of the benzyl ether (DDQ, DCE/pH 7 phosphate buffer, 40 °C, 86%) and the benzenesulfonylamine (sodium naphthalenide, DME, −78 °C, 79%) allowed for the isolation of bis-*exo*-cyclopamine **6** in 18% overall yield from **3**. The hydrogenation of intermediate **4** by using Wilkinson’s catalyst in benzene yielded previously described *exo*-cyclopamine **2** and its C25 epimer **5**. Deprotection with Raney-nickel (EtOH, 78 °C) and then sodium naphthalenide (DME, −78 °C, 41% over two steps) furnished 25-epi-*exo*-cyclopamine **5** in 3% overall yield from **3**.

**Scheme 1:**
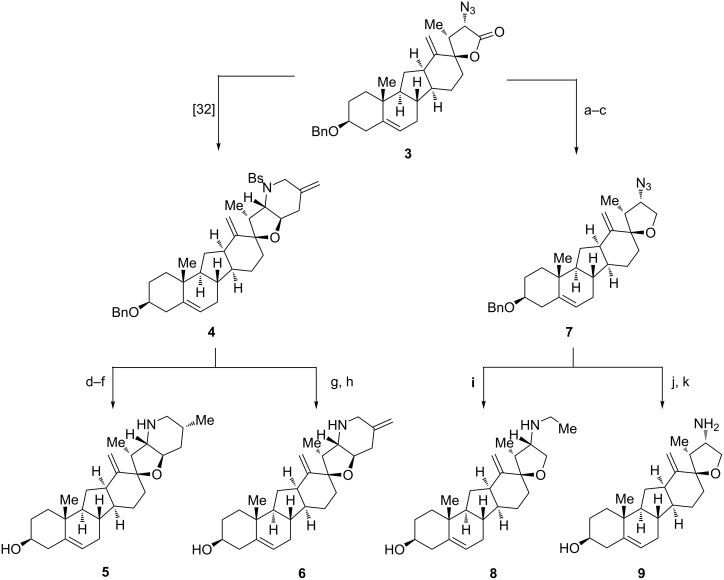
Synthesis of 25-epi-*exo*-cyclopamine **5**, bis-*exo*-cyclopamine **6**, and derivatives **8** and **9**. Reaction conditions: (a) DIBAl-H, THF, −78 °C to −65 °C, 4 h, 95%; (b) Ac_2_O, pyridine, DMAP (cat.), 25 °C, 20 h, quant; (c) BF_3_·Et_2_O, Et_3_SiH, CH_2_Cl_2_, −78 °C to −20 °C, 7 h, 79%; (d) Rh(PPh_3_)_3_Cl, benzene, H_2_, 25 °C, 24 h, quant. (3:1 dr); (e) Raney-nickel (W2), EtOH, 78 °C, 5 min; (f) sodium naphthalenide, DME, −78 °C, 30 min, 41% over two steps; (g) DDQ, DCE/phosphate buffer (pH 7), 40 °C, 95 min, 86%; (h) sodium naphthalenide, DME, −78 °C, 1 h, 79%; (i) Raney-nickel (W2), EtOH, 37%; (j) DDQ, DCE/phosphate buffer (pH 7), 45 °C, 1.5 h, 78%, (k) NaBH_4_, EtOH, 65 °C, 5 d, 62%.

A first set of *exo*-cyclopamine analogs with a modified F-ring was obtained starting again from azide **3** (see Scheme 1). Reduction of the lactone moiety to give a tetrahydrofuran was accomplished in three steps including (1) partial reduction to the lactol (DIBAl-H, THF, −78 °C to −60 °C, 95%), (2) acetylation (Ac_2_O, pyridine, cat. DMAP, quant.) and (3) reductive removal of the so-obtained acetate (Et_3_SiH, BF_3_·Et_2_O, −78 °C to −20 °C, 79%). Removal of the benzyl ether, reduction of the azide moiety and concomitant alkylation was then all effected in one pot by using Raney-nickel in EtOH to give analog **8**, an F-ring-opened *exo*-cyclopamine derivative in 27% overall yield from **3**. Alternatively, the use of previously devised conditions for benzyl deprotection (DDQ, DCE/pH 7 phosphate buffer, 45 °C, 78%) and then sodium borohydride-mediated reduction of the azide (EtOH, 65 °C, 62%) cleanly furnished primary amine **9**, an *exo*-cyclopamine derivative with no F-ring (35% overall yield from **3**).

**Scheme 2:**
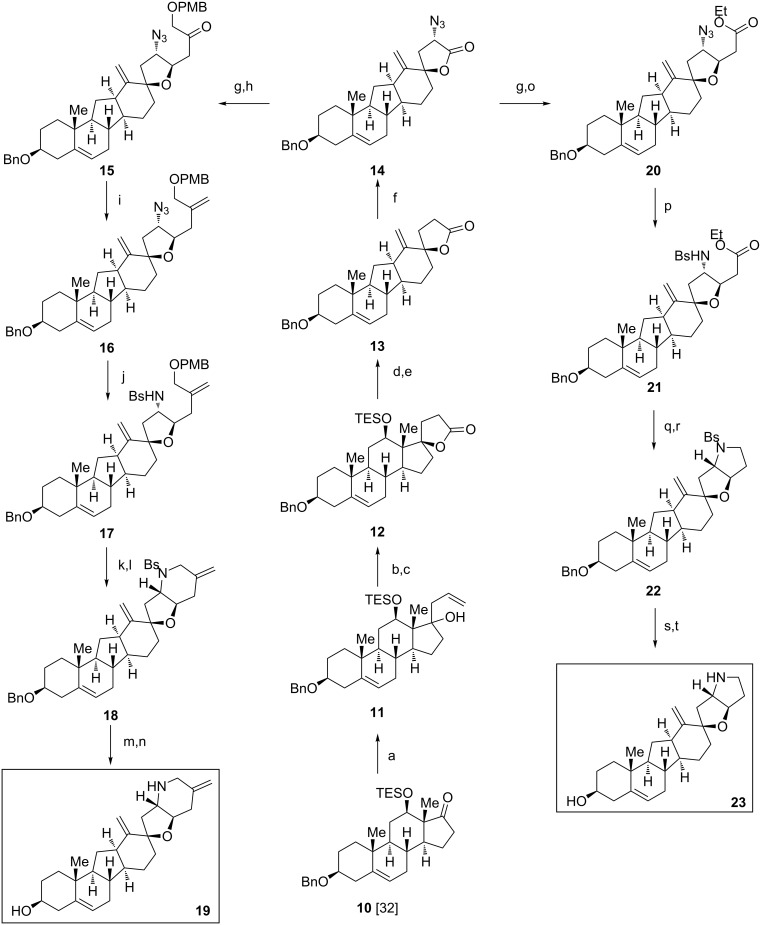
Synthesis of 20-demethyl-bis-*exo*-cyclopamine **19** and F-*nor*-20,25-bis-demethyl-*exo*-cyclopamine **23**. Reaction conditions: (a) allylcerium chloride, THF, 0 °C, 30 min, 93%; (b) 9-BBN, THF, 70 °C, 6 h; then NaBO_3_, 50 °C, 12 h, 91%; (c) BAIB, TEMPO, CH_2_Cl_2_, 25 °C, 3 h, 73%; (d) HF 50 wt % in H_2_O, MeCN, 20 min, 25 °C, 87%; (e) Tf_2_O, pyridine, 0 °C→50 °C, 1.5 h; then additional Tf_2_O, 0 °C→50 °C, 2 h, 46%; (f) LDA, THF, −78 °C to −30 °C, 10 min; then trisylazide, THF, −78 °C, 1.5 h, 43%; g) DIBAl-H, THF, −78 °C to −65 °C, 2 h, 88%; (h) dimethyl (3-((4-methoxybenzyl)oxy)-2-oxopropyl)phosphonate, Ba(OH)_2_, THF/H_2_O, 80 °C, 13 h, 50%; (i) (trimethylsilyl)methylcerium chloride, THF, −78 °C, 30 min; then TMEDA, −78 °C, 15 min; then HF 50 wt % in H_2_O, MeCN, 25 °C, 10 min, 64%; (j) LiAlH_4_, THF, 0 °C→25 °C, 13 h; then benzenesulfonyl chloride, Et_3_N, DMF, 0 °C, 25 min, 69%; (k) DDQ, CH_2_Cl_2_/phosphate buffer (pH 7), 25 °C, 2 h, 59%; (l) *n*-Bu_3_P, DEAD, toluene, 0 °C→25 °C, 12 h, 93%; (m) DDQ, DCE/phosphate buffer (pH 7), 44 °C, 50 min, 70%; (n) sodium naphthalenide, DME, −78 °C, 30 min, 95%; (o) triethyl phosphonoacetate, Ba(OH)_2_, THF, 80 °C, 12 h, 55%; (p) PPh_3_, THF/H_2_O, 50 °C, 24 h; then benzenesulfonyl chloride, Et_3_N, CH_2_Cl_2_, 40 °C, 5 h, 97%; (q) DIBAl-H, THF, −78 °C to −40 °C, 3 h, 97%; (r) *n*-Bu_3_P, DEAD, toluene, 0 °C→25 °C, 24 h, 81%; (s) DDQ, DCE/phosphate buffer (pH 7), 40 °C, 30 min, 62%; (t) sodium naphthalenide, DME, −78 °C, 40 min, 74%.

A second set of F-ring-modified *exo*-cyclopamine derivatives was obtained starting from previously described 12-ß-triethylsilyloxy compound **10** (see Scheme 2) [32]. To construct the spirolactone **12**, a three-step process consisting of (1) allylation (allylcerium chloride, THF, 0 °C, 93%), (2) hydroboration/oxidation (9-BBN, THF, 70 °C; then NaBO_3_, H_2_O, 50 °C, 91%) and finally, (3) oxidative cyclization (BAIB, cat. TEMPO, CH_2_Cl_2_, 25 °C, 73%) was employed. Deprotection of the silyl ether (HF, MeCN, 25 °C, 87%) and base-induced rearrangement of the steroid skeleton via the corresponding triflate (Tf_2_O, pyridine, 0 °C→50 °C, 46%) gave C-*nor*-D-*homo*-derivative **13** which, in turn, was transformed into lactone azide **14** by using Evans’ conditions (LDA, THF, −78 °C to −30 °C, 10 min; then trisylazide, THF, −78 °C, 1.5 h, 43%) [35]. From **14** two additional *exo*-cyclopamine derivatives became accessible. Partial reduction to the lactol (DIBAl-H, THF, −78 °C to −65 °C, 88%) and Horner–Wadsworth–Emmons reaction by using previously reported dimethyl {3-[(4-methoxybenzyl)oxy]-2-oxopropyl}phosphonate [32], (Ba(OH)_2_, THF/H_2_O, 80 °C, 50%) led to **15** which, in turn, was methylenated by employing the Peterson protocol (TMSCH_2_CeCl_2_, THF, −78 °C; then TMEDA; then HF, MeCN, 25 °C, 64%) [36]. Towards this end the azide moiety was reduced and monoprotection of the so-obtained amine as a sulfonamide (LiAlH_4_, THF, 0 °C→25 °C; then benzenesulfonyl chloride, Et_3_N, DMF, 0 °C, 69%) gave **17**. Deprotetion of the 4-methoxybenzyl ether (DDQ, DCE/pH 7 phosphate buffer, 25 °C, 59%) and cyclization under Mitsunobu conditions (*n*-Bu_3_P, DEAD, toluene, 0 °C→25 °C, 93%) yielded piperidine **18**. Deprotection of the benzyl ether by using previously devised conditions (DDQ, DCE/pH 7 phosphate buffer, 44 °C, 70%) and the benzenesulfonylamine (sodium naphthalenide, DME, −78 °C, 95%) gave 20-demethyl-bis-*exo*-cyclopamine **19** in 7% overall yield starting from **14**.

Starting from azido lactone **14** (see Scheme 2) a partial reduction to the lactol (DIBAl-H, THF, −78 °C to −65 °C, 88%) and the Horner–Wadsworth–Emmons reaction by using triethyl phosphonoacetate (Ba(OH)_2_, THF/H_2_O, 80 °C, 55%) led to ethyl ester **20**. Reduction of the azide moiety in **20** was carried out by using Staudinger’s protocol (Ph_3_P, THF/H_2_O, 50 °C). Immediate protection of the obtained amine (benzenesulfonyl chloride, Et_3_N, CH_2_Cl_2_, 40 °C, 97% over 2 steps) gave sulfonylamide **21**. Reduction of the ester in **21** to the primary alcohol (DIBAl-H, THF, −78 °C to −40 °C, 97%) and cyclization by employing Mitsunobu conditions (*n*-Bu_3_P, DEAD, toluene, 0 °C→25 °C, 81%) yielded pyrrolidine **22**. Previously devised conditions for the deprotection (1. DDQ, DCE/pH 7 phosphate buffer, 40 °C; 2. sodium naphthalenide, DME, −78 °C, 46%) finally afforded F-*nor*-20,25-bis-demethyl-*exo*-cyclopamine **23** in 9% overall yield starting from **14**.

**Figure 2:**
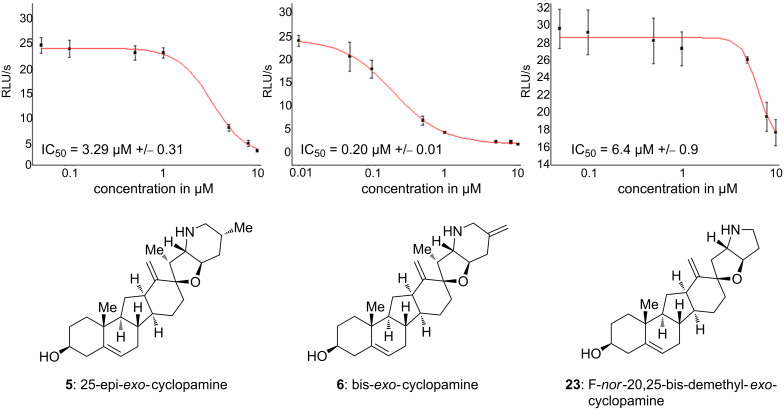
IC_50_ values of Shh inhibition by compounds **5**, **6** and **23** in a Gli1-reporter gene assay. Data were obtained from three independent experiments and represent the mean ± standard deviation.

Next, we examined all synthesized compounds for their ability to inhibit the Gli1-dependent luciferase expression in Shh-LIGHTII cells, a clonal mouse fibroblast cell line which stably incorporates a Gli-dependent firefly luciferase reporter and a constitutive *Renilla* luciferase reporter [37]. The compounds were tested in a concentration range from 0.01 μM to 10 μM. While analogs **8**, **9**, and **19** showed no activity (data not shown) in this concentration range, **5**, **6**, and **23** were active with 25-epi-*exo*-cyclopamine **5** having an IC_50_ of 3.29 ± 0.31 μM, F-*nor*-20,25-bis-demethyl-*exo*-cyclopamine **23** of 6.4 ± 0.9 μM, and bis-*exo*-cyclopamine **6** being the most active with an IC_50_ of 0.20 ± 0.01 μM (see Figure 2).

Given the negative test results of compounds **8** and **9** it becomes evident that the F-ring is necessary for bioactivity. The piperidine moiety provides a rather rigidly placed nitrogen atom. Nevertheless, a pyrrolidine as in compound **23** still provides the correct orientation of the nitrogen atom. Despite compounds **8** and **9** being inactive in the assay, both derivatives induced cytotoxicity in the concentration range tested. Very subtle changes of the conformation of the piperidine ring significantly change bioactivity: While 25-epi-*exo*-cyclopamine **5** shows reduced activity in comparison to *exo*-cyclopamine **2**, bis-*exo*-cyclopamine **6** is the most active compound tested in this study. Furthermore, the methyl group at C-20 seems to have a pronounced effect on the bioactivity, with 20-demethyl-bis-*exo*-cyclopamine **19** being completely inactive in the tested concentration range.

Finally, we studied the stability of all newly synthesized compounds towards acidic conditions. Therefore, they were exposed to a pH of approximately 1 (MeOH, 1 M HCl) for 24 h. After evaporation of all volatiles, ^1^H NMR spectra were acquired and compared to the initially obtained spectra of the pure compounds. While compounds **5**, **6**, **8**, and **9** remained unchanged, compounds **19** and **23** showed decomposition. This experiment emphasizes the importance of the C-21 methyl group for the stability of *exo*-cyclopamine derivatives. All synthesized compounds, the number of steps required, and the respective overall yield starting from **3** or **14**, as well as their biological activity and stability under acidic conditions are summarized in Table 1.

**Table 1:** Synthesized *exo*-cyclopamine derivatives, number of steps, yields, results of their biological testing, and stability towards acid.

Compound	Number of steps from **3** or **14**	Overall yield	Potency in Gli-assay	Stability towards acid (pH ~1, 24 h)

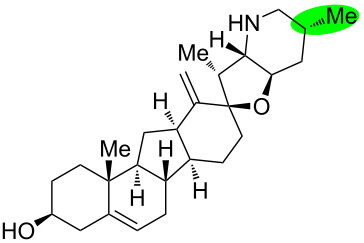 **5**: 25-epi-*exo*-cyclopamine	9 steps from **3**	3%	3.29 ± 0.31 µM	stable
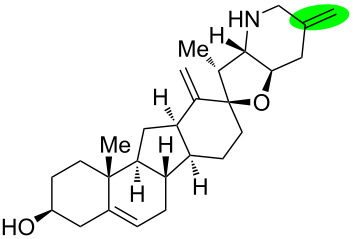 **6**: bis-*exo*-cyclopamine	8 steps from **3**	18%	0.2 ± 0.01 µM	stable
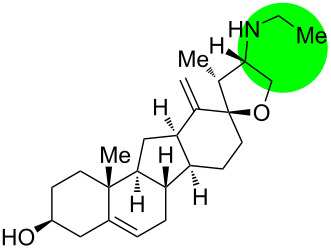 **8**	4 steps from **3**	27%	> 10 µM	stable
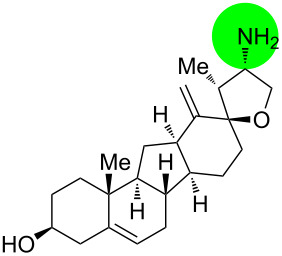 **9**	5 steps from **3**	35%	> 10 µM	stable
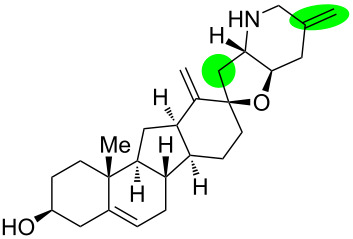 **19**: 20-demethyl-bis-*exo*-cyclopamine	8 steps from **14**	7%	> 10 µM	decomp.
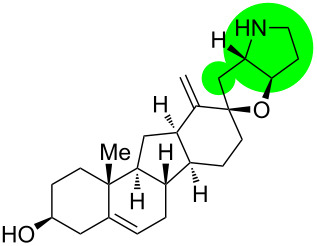 **23**: F-*nor*-20,25-bis-demethyl-*exo*-cyclopamine	7 steps from **14**	9%	6.40 ± 0.90 µM	decomp.
Previously synthesized and biologically evaluated:				
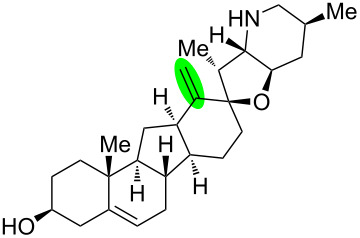 **2**: *exo*-cyclopamine	9 steps from **3**	7%	0.5 µM [34]	stable
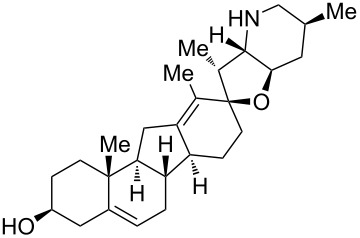 **1**: cyclopamine	10 steps from **3**	5%	5 µM [34]	decomp.

## Conclusion

In conclusion, we succeeded in identifying the new cyclopamine derivative bis-*exo*-cyclopamine **6** which surpasses the biological potency of the parent compound by the 25-fold and is stable at pH 1. Further insights were gained into the structure–activity relationship of F-ring-modified analogs of cyclopamine and the necessity of the C-21 methyl group for bioactivity and acid stability was revealed. Our designed analogs of cyclopamine are accessible in noticeably shorter and higher yielding synthetic routes than the parent compound, a fact that will further contribute to their usefulness in biological and medicinal studies.

## Supporting Information

File 1 Experimental details and analytical data of all synthesized compounds are provided.
