# Supplementary material for: Cyclopamine analogs bearing exocyclic methylenes are highly potent and acid-stable inhibitors of hedgehog signaling
Source: Beilstein J Org Chem. 2013 Oct 31;9:2328–35. doi: 10.3762/bjoc.9.267 (PMC3869244; doi:10.3762/bjoc.9.267)

Supporting Information

for

Cyclopamine analogs bearing exocyclic methylenes are highly potent and acid-stable inhibitors of hedgehog signaling

Johann Moschner[1], Anna Chentsova[1], Nicole Eilert[1], Irene Rovardi[1], Philipp Heretsch*[2] and Athanassios Giannis*[1]

Address: [1]Universität Leipzig, Institut für Organische Chemie, Johannisallee 29, D-04103 Leipzig, Germany and [2]Rice University, Department of Chemistry, BioScience Research Collaborative, 6500 Main St., Houston, Texas 77030

Email: Philipp Heretsch - heretsch@rice.edu; giannis@uni-leipzig.de

*Corresponding author

Experimental details and analytical data of all synthesized compounds are provided

Table of contents

1. General Information S2
2. Synthesis overview S3
3. Experimental Procedures S5
3.1 (+)-25-epi-*exo*-cyclopamine **5** S5
3.2 (+)-*N*-Bs-bis-*exo*-cyclopamine **24** S5
3.3 (−)-Bis-*exo*-cyclopamine **6** S6
3.4 (–)-*O*-Bn-*exo*-C-*nor*-D-*homo*-azidolactol **25** S6
3.5 (−)-*O*-Bn-*exo*-C-*nor*-D-*homo*-azidoacetal **26** S7
3.6 (−)-*O*-Bn-*exo*-C-*nor*-D-*homo*-azidofuran **7** S7
3.7 (–)-*exo*-C-*nor*-D-*homo*-azidofuran **27** S8
3.8 (–)-*exo*-C-*nor*-D-*homo*-aminofuran **8** S8
3.9 (−)-*exo*-C-*nor*-D-*homo*-aminoalcohol **9** S9
3.10 (−)-Alkene **11** and **epi-11** S9
3.11 (–)-Diol **28** and **epi-28** S10
3.12 (−)-Spirolactone **12** and (−)-20-epi-spirolactone **epi-12** S10
3.13 (−)-Hydroxylactone **29** S11
3.14 (−)-*exo*-C-*nor*-D-*homo*-lactone **13** and (−)-*endo* -C-*nor*-D-*homo*-lactone **30** S11
3.15 (–)-*exo*-C-*nor*-D-*homo*-azidolactone **14** and (+)-21-epi-*exo*-C-*nor*-D-*homo*-azidolactone **epi-14** S12
3.16 (–)-*exo*-C-*nor*-D-*homo*-azidolactol **31** S13

3.17 (−)-Furan **15** S13
3.18 (−)-Alkene **16** S14
3.19 (−)-Sulfonamide **17** S15
3.20 (+)-Allylic alcohol **32** S16
3.21 (−)-*N*-Bs-*O*-Bn-20-demethyl-bis-*exo*-cyclopamine **18** S16
3.22 (–)-*N*-Bs-20-demethyl-bis-*exo*-cyclopamine **33** S17
3.23 (−)-20-Demethyl-bis-*exo*-cyclopamine **19** S17
3.24 (−)-Furan **20** S18
3.25 (+)-Sulfonamide **21** S19
3.26 (+)-Alcohol **34** S19
3.27 (+)-*N*-Bs-*O*-Bn-F-*nor*-20,25-bis-demethyl-*exo*-cyclopamine **22** S20
3.28 (+)-*N*-Bs-F-*nor*-20,25-bis-demethyl-*exo*-cyclopamine **35** S20
3.29 (–)-F-*nor*-20,25-bis-demethyl-*exo*-cyclopamine **23** S21
4. Biochemistry S22
5. References S23
6. NMR Spectra S24

1. General Information

General procedures. All reactions were run under an atmosphere of argon unless otherwise indicated. Room temperature refers to 22 °C, ambient pressure to 1013 hPa.

Reagents and anhydrous solvents were transferred via oven-dried syringe or cannula. Flasks were flame-dried under vacuum and cooled under a constant stream of argon.

Tetrahydrofuran was distilled under argon from potassium, dichloromethane from SICAPENT (phosphorus pentoxide on solid support with indicator), ethanol from magnesium ethoxide and diisopropyl amine and triethylamine from calcium hydride. Benzene, toluene, 1,2-dimethoxyethane, 1,2-dichloroethane, acetone, acetonitrile and pyridine were purchased from Acros or Sigma-Aldrich (anhydrous over molecular sieves).

All other chemicals were purchased from ABCR, Acros, Sigma-Aldrich, Alfa Aesar, Fluorochem, Merck and TCI Europe at highest commercially available purity and used as such.

Reactions were monitored by thin-layer chromatography using Merck silica gel 60 F_{254} TLC aluminium sheets and visualized with ceric ammonium molybdate, potassium permanganate or vanillin staining solution. Chromatographic purification was performed as flash chromatography on Acros silica gel 35–70, 60 Å, using a forced flow of eluent (method of Still) or as preparative TLC on Merck silica gel 60 F_{254} glass plates with concentration zone. Concentration under reduced pressure was performed by rotary evaporation at 40 °C at the appropriate pressure.

Yields refer to chromatographically purified and spectroscopically pure compounds.

NMR spectra were recorded on a Bruker Avance 700 (operating at 700 MHz for ^{1}H and 175 MHz for ^{13}C), Varian Mercury plus 400 (operating at 400 MHz for ^{1}H and 100 MHz for ^{13}C) and a Varian Mercury plus 300 (operating at 300 MHz for ^{1}H and 75 MHz for ^{13}C acquisitions). Chemical shifts δ are reported in ppm with the solvent resonance as the internal standard (d_1-chloroform: 7.260 (^{1}H NMR), 77.16 (^{13}C NMR); d_4-methanol: 3.310 (^{1}H NMR), 49.00 (^{13}C NMR). Coupling constants *J* are reported in Hertz (Hz). Multiplicities are classified by the following abbreviations: s = singlet, d = doublet, t = triplet, q = quartet, p = quintet and combinations thereof, or m = multiplet or br = broad signal.

Where 2D-spectra were recorded and allowed complete assignment of all hydrogen and carbon-atoms of a compound, spectral data include this assignment using common steroid numbering. Where this is not the case, all hydrogen signals below 2 ppm are omitted and only methyl groups and isolated signals in this range are listed. All spectra can be found as copies at the end of the experimental section.

High resolution mass spectra were obtained on a Bruker Daltonics ESI-FT-ICR-MS APEX II. IR spectra were obtained on an ATI/MATTSON Genesis FT-IR and JASCO FT/IR-4100typeA as thin film (in CCl_4) or KBr disk. Absorbance frequencies are reported in reciprocal centimetres (cm^{-1}).

Melting points were measured on a Boetius-micro hot stage and are uncorrected.

Optical rotation data was obtained with a Schmidt+Haensch Polartronic MHZ-8 at the sodium-D line (589 nm) using a 50 mm path-length cell and solvents and concentrations as indicated.

2. Synthesis overview

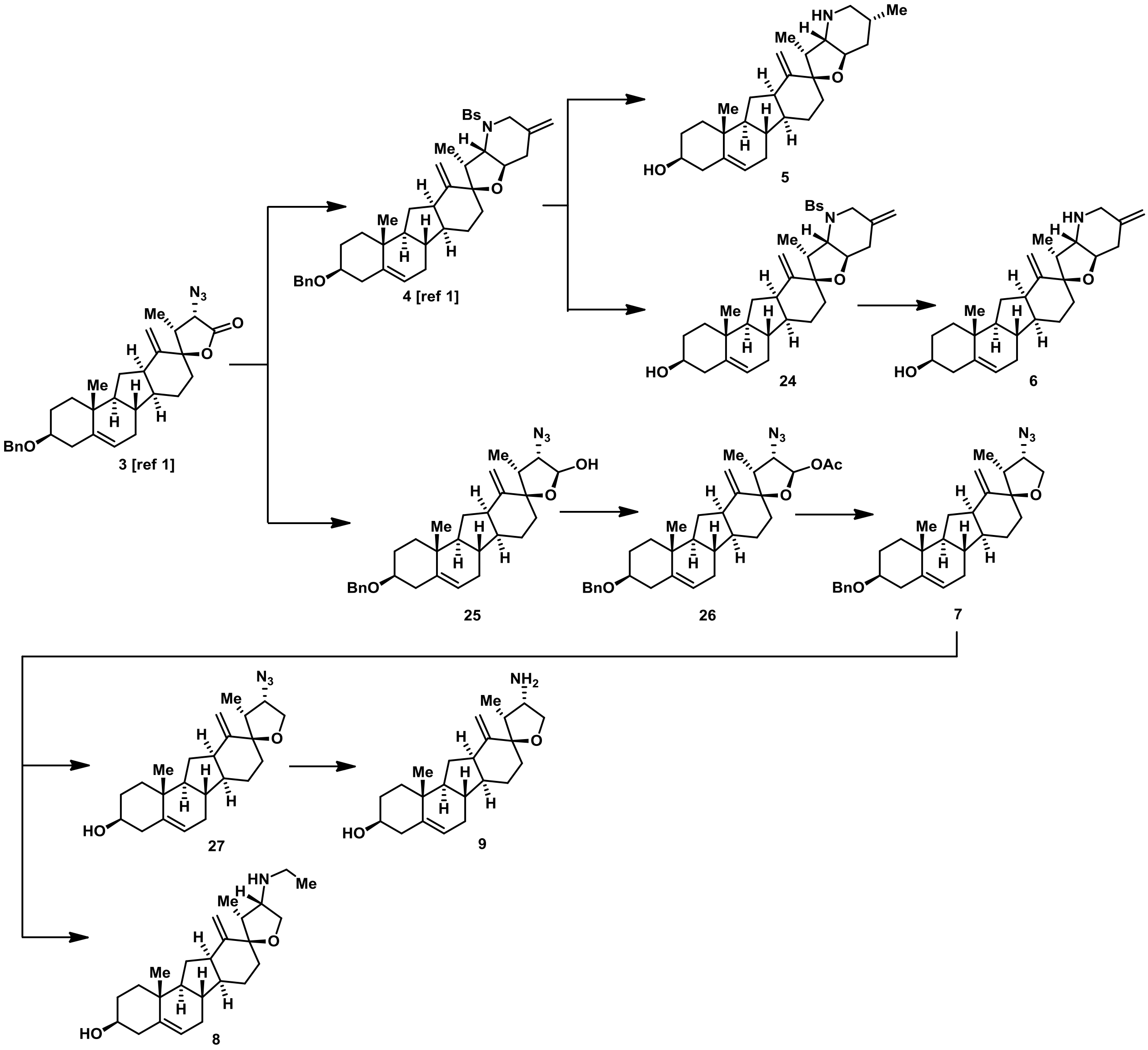

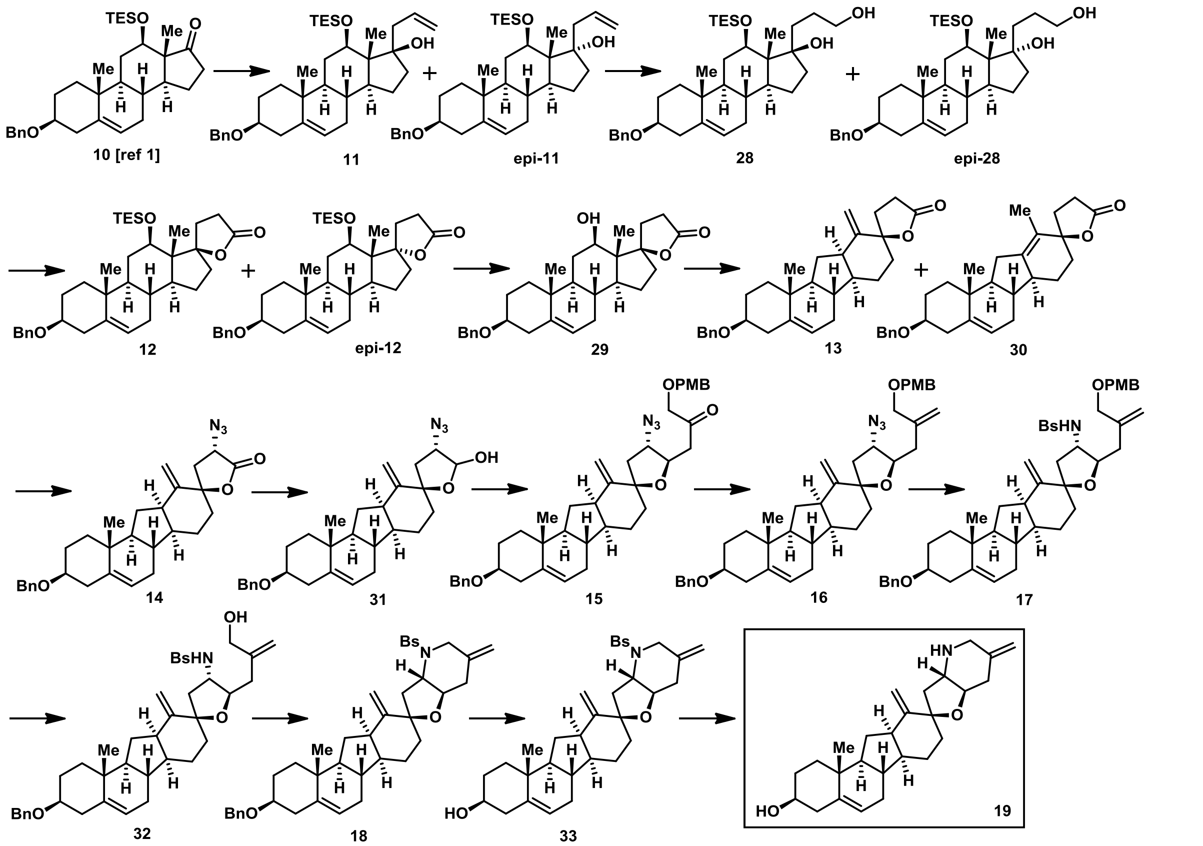
10 [ref 1]
11
epi-11
28
epi-28
12
epi-12
29
13
30
14
31
15
16
17
32
18
33
19
TESO
BnO
HO
OPMB
BsHN
Bs
OH

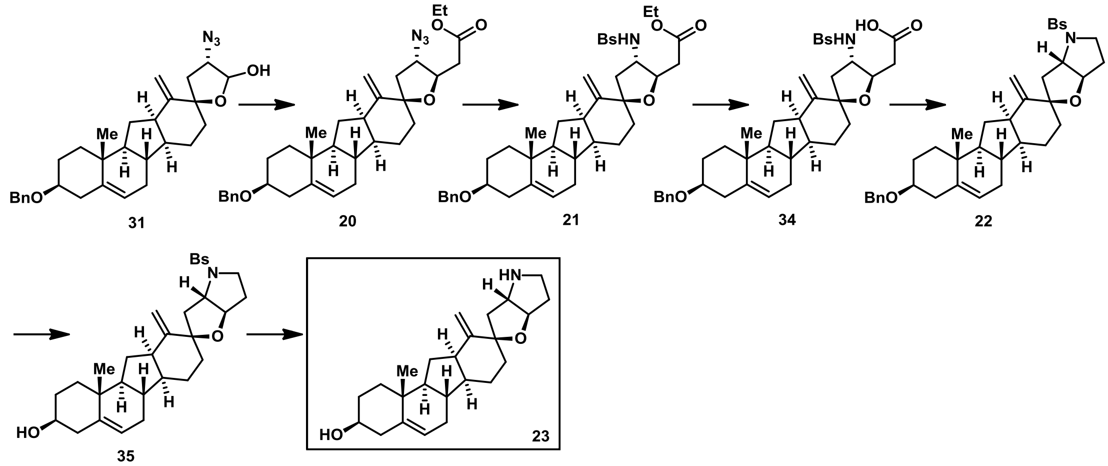
31
20
21
34
22
35
23
BnO
HO
BsHN
Et
Bs

3. Experimental Procedures

3.1 (+)-25-epi-*exo*-cyclopamine **5**

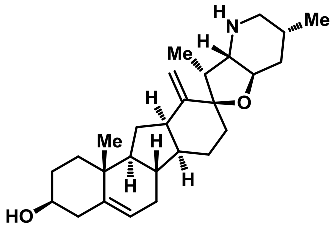

5

To a solution of 25-epi-*N*-Bs-*O*-Bn-*exo*-cyclopamine [1] (10.2 mg, 15.8 µmol) in EtOH (1 mL) freshly prepared Raney-nickel (W2) (app. 0.25 g) in EtOH (2 mL) was added and the suspension was heated to reflux under vigorous stirring for 5 min. The mixture was allowed to cool to room temperature, filtered through Celite and washed with EtOH (4 x 5 mL). The solvent was removed under reduced pressure, the crude material was redissolved in CH_2Cl_2 (2 mL) and filtered (paper), washing several times with CH_2Cl_2. The crude 25-epi-*N*-Bs-*exo*-cyclopamine was dried in vacuum for 12 h and then was redissolved in 1,2-dimethoxyethane (1 mL) and cooled to −78 °C under stirring. To this a solution of freshly prepared sodium naphthalenide (0.5 M in dimethoxyethane, 250 µL, 125 µmol) was added dropwise and stirring at this temperature was continued for 30 min. Then, saturated aqueous $NaHCO_3$ solution (5 mL) was introduced, the suspension was allowed to warm to room temperature and was extracted with CH_2Cl_2 (5 x 5 mL). The combined organic extracts were dried (Na_2SO_4), and all volatiles were removed under reduced pressure. Column chromatography (SiO_2; $CHCl_3$/EtOH/NH_3 (25% aq.), 95:5:0.5 *v*/*v*/*v*) yielded pure compound **5** (2.6 mg, 6.5 µmol, 41% over two steps) as a waxy solid. m.p.: 210-215°C; TLC ($CHCl_3$/EtOH/NH_3(25% aq.), 95:5:0.5, *v*/*v*/*v*): R_F = 0.18; $[\alpha]_D^{22}$ (deg cm^3 g^{-1} dm^{-1}) = +42 (c = 0.0015 g cm^{-3} in CH_2Cl_2); IR (KBr): ν_{max} 3420, 2950, 1648, 1458, 1376, 1350, 1170, 1106, 1060, 1039, 987, 908, 799 cm^{-1}; ^{1}H NMR (400 MHz, $CDCl_3$) δ 5.38 (m, 2 H, H-6, H-18), 4.93 (s, 1 H, H-18), 3.68 (dt, *J* = 11.0, 5.0 Hz, 1 H, H-23), 3.52 (m, 1 H, H-3), 3.51 (m, 1 H, H-26), 2.86 (m, 1 H, H-12), 2.85 (m, 1 H, H-20), 2.70 (m, 1 H, H-22), 2.68 (m, 1 H, H-26), 2.36 (m, 1 H, H-4), 2.34 (m, 1 H, H-4), 2.27 (m, 1 H, H-7), 2.24 (m, 1 H, H-2), 2.17 (m, 1 H, H-16), 2.06 (m, 1 H, H-11), 1.77 (m, 2 H, H-1, H-25), 1.73 (m, 3 H, H-11, H-15, H-24), 1.65 (m, 1 H, H-14), 1.64 (m, 2 H, H-1, H-16), 1.60 (m, 1 H, H-7), 1.55 (m, 1 H, H-2), 1.48 (m, 1 H, H-15), 1.43 (m, 1 H, H-24), 1.36 (m, 1 H, H-9), 1.33 (m, 1 H, H-8), 1.16 (td, *J* = 13.7, 4.1 Hz, 1 H, H-1), 1.08 (d, *J* = 6.8 Hz, 3 H, H-21), 1.04 (d, *J* = 6.8 Hz, 3 H, H-27), 0.99 (s, 3 H, H-19); ^{13}C NMR (75 MHz, $CDCl_3$) δ 154.9 (C-13), 141.9 (C-5), 122.2 (C-6), 108.5 (C-18), 88.0 (C-17), 76.4 (C-3), 72.1 (C-23), 64.1 (C-22), 53.7 (C-26), 52.5 (C-9), 47.5 (C-14), 41.9 (C-12), 40.9 (C-20), 38.7 (C-4), 38.4 (C-1), 37.1 (C-8), 36.5 (C-10), 36.4 (C-24), 32.4 (C-16), 31.6 (C-7), 31.6 (C-2), 29.1 (C-25), 26.3 (C-11), 23.8 (C-15), 19.1 (C-19), 18.7 (C-27), 10.1 (C-21); ESI-HRMS (*m/z*): $[M+H]^+$ calcd for $C_{27}H_{42}NO_2$: 412.32101, found: 412.32132.

3.2 (+)-*N*-Bs-bis-*exo*-cyclopamine **24**

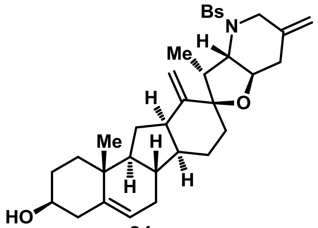

24

To a solution of 25-epi-*N*-Bs-*O*-Bn-bis-*exo*-cyclopamine [1] (11.1 mg, 17.3 µmol) in 1,2-dichloroethane (1.4 mL) and phosphate buffer (0.17 mL, pH 7) was added 2,3-dichloro-5,6-dicyano-1,4-benzoquinone (33.8 mg, 148.9 µmol) at 40 °C and stirring was continued for 95 min. The reaction mixture was quenched with saturated aqueous $NaHCO_3$ solution and cooled down to room temperature. The aqueous phase was extracted with CH_2Cl_2 (3 x 5 mL), the combined organic layers were washed with brine (3 mL), dried ($MgSO_4$) and all volatiles were removed under reduced pressure. Column chromatography (SiO_2; *n*-hexane/EtOAc, 5:1 → 2:1 *v*/*v*) yielded pure **24** (8.2 mg, 14.9 µmol, 86%) as a waxy solid; mp.: 187-190°C; TLC (*n*-hexane/EtOAc, 2:1 *v*/*v*): R_F = 0.26; $[\alpha]_D^{22}$ (deg cm^3 g^{-1} dm^{-1}) = +19 (c = 0.0069 g cm^{-3} in $CHCl_3$); IR (KBr): ν_{max} 3434, 1635, 1356, 1171, 810, 603, 576 cm^{-1};

^{1}H NMR (300 MHz, $CDCl_3$) δ 7.70 (d, *J* = 7.2 Hz, 2 H, ortho-H Bs), 7.62 (t, *J* = 7.2 Hz, 1 H, para-H Bs), 7.51 (t, *J* = 7.2 Hz, 2 H, meta-H Bs), 5.36 (m, 1H), 5.13 (m, 1H), 4.98 (m, 1H), 4.92 (m, 1H), 4.87 (m, 1H), 3.99 (d, *J* = 12.6 Hz, 1H), 3.86 (ddd, *J* = 11.9, 9.9, 4.7 Hz, 1H), 3.53 (m, 1H), 2.88 (m, 1H), 2.85 (m, 1H), 2.73 (dd, *J* = 12.6, 4.7 Hz, 1H), 2.66 (dd, *J* = 9.9, 5.1 Hz, 1H), 2.37 (ddd, *J* = 13.0, 4.9, 2.2 Hz, 1H), 2.23 (m, 1H), 2.11 (m, 1H), 1.33 (d, *J* = 6.7 Hz, 2H), 0.98 (s, 1H); ^{13}C NMR (100 MHz, $CDCl_3$) δ 154.0, 141.8, 137.4, 133.8 (ipso-C Bs), 133.5 (para-C Bs), 129.0 (meta-C Bs), 128.6 (ortho-C Bs), 112.2, 115.9, 108.8, 89.2, 74.6, 72.0, 62.7, 54.2, 53.7, 46.8, 42.1, 41.9, 40.6, 38.4, 37.8, 37.7, 37.2, 32.3, 31.5, 30.9, 26.0, 23.3, 19.2, 9.7; ESI-HRMS (m/z): $[M+Na]^+$ calcd for $C_{33}H_{43}O_4SNa$: 572.28050, found: 572.28063.

3.3 (−)-Bis-*exo*-cyclopamine **6**

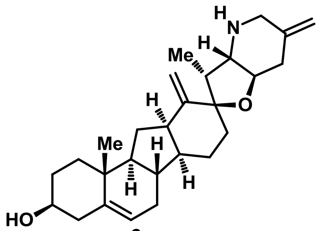

6

N-Bs-bis-*exo*-cyclopamine **24** (6.8 mg, 12.3 µmol) was dissolved in 1,2-dimethoxyethane (1 mL) and cooled to −78 °C under stirring. A solution of freshly prepared sodium naphthalenide (0.5 M in dimethoxyethane, 250 µL, 125 µmol) was added dropwise and stirring at this temperature was continued for 1 h. Then, saturated aqueous $NaHCO_3$ solution (5 mL) was introduced, the suspension was allowed to warm to room temperature, and was extracted with CH_2Cl_2 (5 x 5 mL). The combined organic extracts were dried (Na_2SO_4), and all volatiles were removed under reduced pressure. Column chromatography (SiO_2; $CHCl_3$/EtOH/NH_3 (25% aq.), 100:0:0.5 → 99:1:0.5 *v*/*v*/*v*) yielded pure **6** (4.0 mg, 9.8 µmol, 79%) as a colorless solid; mp.: 230-233°C; TLC ($CHCl_3$/EtOH/NH_3 (25% aq), 95:5:0.5, *v*/*v*/*v*): R_F = 0.35; $[\alpha]_D^{25}$ (deg cm^3 g^{-1} dm^{-1}) = –22 (c = 0.0023 g cm^{-3} in $CHCl_3$); IR (KBr): ν_{max} 3678, 3290, 2925, 2854, 1638, 1458, 1060, 901 cm^{-1}; ^{1}H NMR (300 MHz, $CDCl_3$) δ 5.37 (s, 1 H, H-18), 5.34 (m, 1H, H-6), 4.96 (s, 1H, H-18), 4.88 (s, 1 H, H-27), 4.85 (s, 1H, H-27), 3.49 (m, 1H, H-3), 3.48 (m, 1H, H-26), 3.45 (m, 1H, H-23), 3.25 (d, *J* = 13.8 Hz, 1H, H-26), 2.93 (dd, *J* = 9.9, 6.6 Hz, 1H, H-22), 2.89 (dd, *J* = 12.6, 4.5, 1H, H-24), 2.70 (td, *J* = 8.5, 5.6 Hz, 1H, H-12), 2.35 (m, 1H, H-4), 2.34 (m, 1H, H-24), 2.26 (m, 1H, H-20), 2.22 (m, 1H, H-4), 2.12 (m, 1H, H-7), 1.84 (m, 1H, H-16), 1.83 (m, 1H, H-2), 1.78 (m, 1H, H-11), 1.75 (m, 1H, H-1), 1.74 (m, 1H, H-11), 1.73 (m, 1H, H-15), 1.66 (m, 1H, H-16), 1.62 (m, 1H, H-7), 1.61 (m, 1H, H-14), 1.52 (m, 1H, H-2), 1.39 (m, 1H, H-15), 1.34 (m, 2H, H-9, H-8), 1.17 (m, 1H, H-1), 1.01 (d, *J* = 7.6 Hz, 3H, H-21), 0.99 (s, 3H, H-19); ^{13}C NMR (75 MHz, $CDCl_3$) δ 154.7 (C-13), 143.7 (C-25), 141.9 (C-5), 122.2 (C-6), 112.8 (C-27), 108.6 (C-18), 89.2 (C-17), 77.3 (C-23), 72.0 (C-3), 63.1 (C-22), 53.7 (C-9), 53.4 (C-26), 47.3 (C-14), 41.9 (C-4), 41.3 (C-12), 40.4 (C-24), 40.3 (C-20), 38.6 (C-8), 38.4 (C-1), 37.2 (C-10), 32.4 (C-16), 31.6 (C-2), 31.0 (C-7), 26.3 (C-11), 23.7 (C-15), 19.1 (C-19), 9.8 (C-21); ESI-HRMS (m/z): $[M+Na]^+$ calcd for $C_{27}H_{39}NO_2Na$: 432,28730, found 432.28762.

3.4 (−)-*O*-Bn-*exo*-C-*nor*-D-*homo*-azidolactol **25**

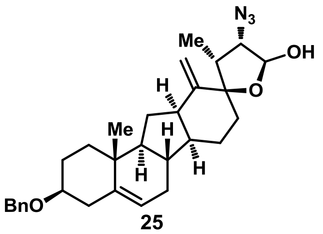

25

To a stirred solution of *exo*-C-*nor*-D-*homo*-azidolactone **3** [1] (500 mg, 0.410 mmol) in THF (5 mL) at −78 °C was dropwise added diisobutylaluminumhydride (1.2 M in toluene; 1.70 mL, 2.05 mmol) and stirring was continued at this temperature for 1 h and then at −65 °C for 4 h. The reaction mixture was quenched with MeOH (0.2 mL) and Rochelle-salt solution (10 wt % in H_2O) (20 mL), diluted with CH_2Cl_2 (20 mL) and warmed to room temperature. After stirring for 1 h the phases were separated and the aqueous layer was extracted with CH_2Cl_2

(2 x 20 mL). The combined organic extracts were dried ($MgSO_4$) and the solvents were evaporated under reduced pressure. Purification by column chromatography (SiO_2; *n*-hexane/EtOAc, 15:1 v/v) yielded pure **25** (190 mg, 0.388 mmol, 95%) as a colorless foam; m.p. 90-95°C; TLC (*n*-hexane/EtOAc, 3:1 *v/v*): R_F = 0.52; $[\alpha]_D^{22}$ (deg cm^3 g^{-1} dm^{-1}) = –32 (c = 0.0050 g cm^{-3} in $CHCl_3$); IR (KBr): ν_{max} 2101, 750, 698 cm^{-1}; ^{1}H NMR (300 MHz, $CDCl_3$) δ 7.36-7.23 (m, 5 H), 5.53 (m, 1 H), 5.36 (m, 2 H), 5.01 (s, 1 H), 4.57 (s, 2 H), 3.98 (dd, *J* = 5.7, 2.6 Hz, 1 H), 3.28 (tt, *J* = 11.3, 4.5 Hz, 1 H), 3.15 (d, *J* = 4.0 Hz, 1 H), 2.68 (m, 1 H), 2.57 (m, 1 H), 2.49 (ddd, *J* = 13.2, 4.6, 2.1 Hz, 1 H), 2.26 (m, 1 H), 2.14 (m, 1 H), 1.11 (d, *J* = 7.2 Hz, 3 H), 1.00 (s, 3 H); ^{13}C NMR (75 MHz, $CDCl_3$) δ 153.7, 142.0, 139.2, 128.5, 127.7, 127.6, 122.1, 110.2, 99.8, 90.0, 78.8, 70.7, 70.1, 53.9, 46.6, 43.8, 42.1, 40.4, 38.7, 38.4, 37.4, 32.5, 31.5, 28.3, 28.3, 24.1, 19.0, 10.2; ESI-HRMS (m/z): $[M+Na]^+$ calcd for $C_{30}H_{39}N_3O_3Na$: 512.28836, found: 512.28800, $[2M+Na]^+$ calcd for $C_{60}H_{78}N_6O_6Na$: 1001.58751, found: 1001.58682.

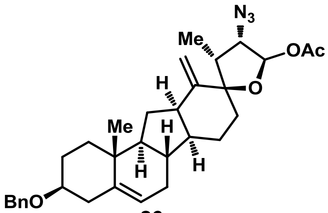

26

3.5 (−)-*O*-Bn-*exo*-C-*nor*-D-*homo*-azidoacetal **26**

To a solution of azidolactol **25** (112.0 mg, 228.9 µmol) and DMAP (3 mg, 24 µmol) in pyridine (1.5 mL) acetic anhydride (26 µL, 287.1 µmol) was added dropwise at room temperature and stirred for 20 h. The reaction mixture was diluted with saturated aqueous NH_4Cl solution (5 mL). The aqueous phase was extracted with EtOAc (3 x 5 mL), the combined organic extracts were washed with brine (10 mL), dried ($MgSO_4$) and all volatiles were removed under reduced pressure. Column chromatography (SiO_2; *n*-hexane/EtOAc, 8:1 *v/v*) yielded pure **26** (121.7 mg, 228.9 µmol, quant.) as colorless crystals; mp.: 103-105°C; TLC (*n*-hexane/EtOAc, 5:1 *v/v*): R_F = 0.46; $[\alpha]_D^{22}$ (deg cm^3 g^{-1} dm^{-1}) = –14 (c = 0.0101 g cm^{-3} in $CHCl_3$); IR (KBr): ν_{max} 2932, 2105, 1734, 1233, 1110, 733, 696 cm^{-1}; ^{1}H NMR (400 MHz, $CDCl_3$) δ 7.36 (m, 2 H, ortho-H Bn), 7.34 (m, 2 H, meta-H Bn), 7.28 (m, 1 H, para-H Bn), 6.19 (d, *J* = 2.1 Hz, 1 H), 5.38 (s, 2H), 5.00 (s, 1 H), 4.60 (s, 2 H), 4.11 (dd, *J* = 5.6, 2.1 Hz, 1 H), 3.31 (tt, *J* = 9.8, 7.9, 3.7 Hz, 1 H), 2.70 (m, 1 H), 2.57 (m, 1 H), 2.52 (m, 1 H), 2.30 (m, 1 H), 2.16 (m, 1 H), 2.13 (s, 3 H), 1.99 (m, 2 H), 1.18 (d, *J* = 7.2 Hz, 4 H), 1.02 (s, 3 H); ^{13}C NMR (100 MHz, $CDCl_3$) δ 170.2, 153.2, 141.9, 139.1 (ipso-C Bn), 128.5 (meta-C Bn), 127.7 (ortho-C Bn), 127.5 (para-C Bn), 122.1, 109.7, 99.1, 91.4, 78.7, 70.0, 69.7, 53.7, 46.6, 44.1, 41.8, 40.6, 38.7, 38.3, 37.3, 32.4, 31.5, 28.3, 28.2, 24.2, 21.4, 19.0, 10.2; ESI-HRMS (m/z): $[M+Na]^+$ calcd for $C_{32}H_{41}N_3O_4Na$: 554.29893, found 554.29878.

3.6 (−)-*O*-Bn-*exo*-C-*nor*-D-*homo*-azidofuran **7**

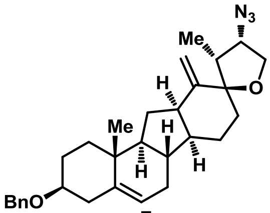

7

To a solution of azidoacetal **26** (74.2 mg, 139.6 µmol) in CH_2Cl_2 (3.7 mL) were added triethylsilane (61.0 µL, 340.7 µmol) and boron trifluoride diethyl etherate (25.5 µL, 163.5 µmol) at −78 °C. The mixture was stirred for 30 min at −78 °C and for 6 h at −20 °C. Saturated aqueous NH_4Cl solution (5 mL) was added and the mixture was allowed to warm to room temperature and was stirred for 15 min. The aqueous phase was extracted with EtOAc (2 x 5 mL), the combined extracts were washed with brine (5 mL), dried ($MgSO_4$) and all volatiles were removed under reduced pressure. Column chromatography (SiO_2; *n*-hexane/EtOAc, 20:1 *v/v*) yielded pure **7** (52.2 mg, 110.3 µmol, 79%) as a waxy solid; mp.: 72-74°C; TLC (*n*-hexane/EtOAc, 20:1 *v/v*): R_F = 0.33, $[\alpha]_D^{22}$ (deg cm^3 g^{-1} dm^{-1}) = –32 (c = 0.0112 g cm^{-3} in $CHCl_3$); IR (CCl_4): ν_{max} 2954, 2111, 1387, 1278, 1095, 789, 698 cm^{-1}, ^{1}H NMR (400 MHz, $CDCl_3$) δ 7.37 (m,

2 H, ortho-H Bn), 7.35 (m, 2 H, meta-H Bn), 7.29 (m, 1 H, para-H Bn), 5.38 (m, 1 H), 5.23 (m, 1 H), 4.96 (m, 1 H), 4.59 (s, 2 H), 4.19 (m, 2 H), 3.78 (m, 1 H), 3.31 (tt, *J* = 11.2, 4.5 Hz, 1 H), 2.71 (m, 1 H), 2.52 (ddd, *J* = 13.2, 4.7, 2.2 Hz, 1 H), 2.39 (p, *J* = 6.9 Hz, 1 H), 2.29 (m, 1 H), 2.17 (m, 1 H), 1.99 (m, 1 H), 1.12 (d, *J* = 7.1 Hz, 3 H), 1.03 (s, 3 H); ^{13}C NMR (100 MHz, $CDCl_3$) δ 153.2, 142.1, 139.2 (ipso-C Bn), 128.5 (meta-C Bn), 127.7 (ortho-C Bn), 127.5 (para-C Bn), 122.1, 108.3, 88.0, 78.8, 70.1, 69.1, 64.7, 53.9, 46.5, 44.6, 42.0, 39.9, 38.7, 37.5, 31.4, 30.6, 28.3, 27.7, 24.2, 19.0, 10.0; ESI-HRMS (m/z): $[M+Na]^+$ calcd for $C_{30}H_{39}N_3O_2Na$: 496,29345, found 496.29377; $[2M+Na]^+$ calcd for $C_{60}H_{78}N_6O_4Na$: 969,59768, found: 969.59837.

3.7 (–)-*exo*-C-*nor*-D-*homo*-azidofuran **27**

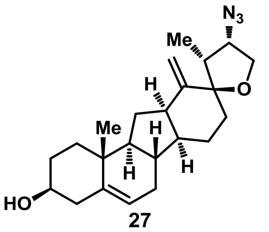

27

To a mixture of azidofuran **7** (34.5 mg, 72.8 µmol) in 1,2-dichloroethane (6 mL) and phosphate buffer (pH 7, 740 µL) was added 2,3-dichloro-5,6-dicyano-1,4-benzoquinone (137.0 mg, 603.5 µmol) in one portion at 45 °C. After stirring for 90 min at 45 °C the reaction mixture was quenched with saturated aqueous $NaHCO_3$ solution and cooled down to room temperature. The aqueous phase was extracted with CH_2Cl_2 (3 x 5 mL), the combined organic layers were dried ($MgSO_4$) and all volatiles have been removed under reduced pressure. Column chromatography (SiO_2; *n*-hexane/EtOAc, 5:1 → 3:1) yielded pure **27** (21.8 mg, 56.8 µmol, 78%) as a colorless solid; mp 86-89°C; TLC (*n*-hexane/EtOAc, 3:1 *v*/*v*): R_F = 0.17; $[\alpha]_D^{24}$ (deg cm^3 g^{-1} dm^{-1}) = –54 (c = 0.0102 g cm^{-3} in $CHCl_3$); IR (CCl_4): ν_{max} 3408, 2927, 2105, 1637, 1463, 1383, 1053, 788 cm^{-1}; ^{1}H NMR (300 MHz, $CDCl_3$) δ 5.35 (m, 1 H, H-6), 5.20 (s, 1 H, H-18), 4.93 (s, 1 H, H-18), 4.19 (m, 1 H, H-22), 4.15 (m, 1 H, H-23), 3.74 (m, 1 H, H-23), 3.52 (m, 1 H, H-3), 2.68 (m, 1H, H -12), 2.37 (m, 1 H, H-4), 2.36 (m, 1 H, H-20), 2.21 (m, 1 H, H-4), 2.13 (m, 1 H, H-7), 1.83 (m, 1 H, H-2), 1.79 (m, 1 H, H-11), 1.74 (m, 1 H, H-1), 1.69 (m, 2 H, H-16), 1.68 (m, 1 H, H-11), 1.67 (m, 1 H, H-15), 1.63 (m, 1 H, H-14), 1.62 (m, 1 H, H-7), 1.54 (m, 1 H, H-2), 1.36 (m, 1 H, H-15), 1.35 (m, 1 H, H-8), 1.33 (m, 1 H, H-8), 1.16 (dt, *J* = 13.6, 3.6 Hz, 1 H, H-1), 1.10 (d, *J* = 7.2 Hz, 3 H, H-21), 0.99 (s, 3 H, H-19); ^{13}C NMR (75 MHz, $CDCl_3$) δ 153.2 (C-13), 141.9 (C-5), 122.3 (C-6), 108.3 (C-18), 88.0 (C-17), 72.0 (C-3), 69.1 (C-23), 64.7 (C-22), 53.8 (C-9), 46.5 (C-14), 44.6 (C-20), 42.0 (C-12), 41.9 (C-4), 39.9 (C-8), 38.4 (C-1), 37.1 (C-10), 31.6 (C-2), 31.4 (C-7), 30.6 (C-16), 27.8 (C-11), 24.2 (C-15), 19.1 (C-19), 10.0 (C-21); ESI-HRMS (m/z): $[M+Na]^+$ calcd for $C_{23}H_{34}N_3O_2Na$: 406.24650, found 406.24614; $[2M+Na]^+$ calcd for $C_{46}H_{68}N_3O_4Na$: 789.50378, found: 789.50336.

3.8 (–)-*exo*-C-*nor*-D-*homo*-aminofuran **8**

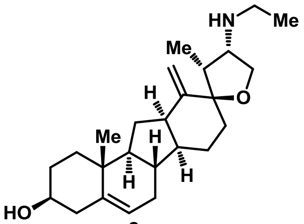

8

To a solution of azidofuran **7** (34.5 mg, 75.1 µmol) in EtOH was added freshly prepared Raney-nickel (W2) and stirred for 4 h. The reaction mixture was filtered through Celite and washed with EtOH (4 x 5 mL). The crude was dissolved in CH_2Cl_2 and filtered (paper) and washed with CH_2Cl_2 (4 x 5 mL). All volatiles were removed under reduced pressure. Column chromatography (SiO_2; $CHCl_3$/EtOH/NH_3 (25% aq.), 95:5:0.5 *v*/*v*/*v*) yielded pure **8** (10.7 mg, 27.8 µmol, 37%) as colorless crystals; m.p.: 125-130 °C; TLC ($CHCl_3$/EtOH/NH_3 (25% aq.), 95:5:0.5 *v*/*v*/*v*): R_F = 0.15; $[\alpha]_D^{24}$ (deg cm^3 g^{-1} dm^{-1}) = –10 (c = 0.0047 g cm^{-3} in CH_2Cl_2); IR (KBr): ν_{max} 3408, 1730, 1384, 1095, 712 cm^{-1}; ^{1}H NMR (300 MHz, $CDCl_3$) δ 5.35 (m, 1 H, H-6), 5.17 (s, 1 H, H-18), 4.89 (s, 1 H, H-18), 4.11 (t, *J* = 6.9 Hz, 1 H, H-23), 3.52 (m, 2 H, H-22, H-3), 3.45 (m, 1 H, H-23), 2.71 (m, 1 H, H-12), 2.66 (m, 1 H, H-24), 2.55 (m, 1 H, H-24), 2.36 (m, 1 H, H-4), 2.25 (m, 1 H, H-20), 2.21 (m, 1 H, H-4), 2.08 (m, 1 H, H-7), 1.80 (m, 1 H,

H-2), 1.78 (m, 1 H, H-10), 1.77 (m, 1 H, H-16), 1.74 (m, 1 H, H-16), 1.73 (m, 1 H, H-1), 1.67 (m, 1 H, H-15), 1.59 (m, 1 H, H-14), 1.55 (m, 1 H, H-11), 1.53 (m, 1 H, H-15), 1.51 (m, 1 H, H-2), 1.38 (m, 1 H, H-8), 1.31 (m, 1 H, H-9), 1.14 (m, 1 H, H-1), 1.10 (t, *J* = 7.2 Hz, 3 H, H-25), 0.99 (s, 3 H, H-19), 0.93 (d, *J* = 6.9 Hz, 3 H, H-21); ^{13}C NMR (75 MHz, $CDCl_3$) 153.2 (C-13), 142.1 (C-5), 122.2 (C-6), 107.5 (C-18), 89.4 (C-17), 72.0 (C-3), 69.8 (C-23), 59.7 (C-22), 53.8 (C-9), 46.4 (C-14), 42.9 (C-24), 42.1 (C-12), 41.9 (C-4), 38.4 (C-1), 38.0 (C-20), 37.6 (C-8), 37.3 (C-10), 31.6 (C-2), 31.4 (C-11), 31.0 (C-7), 26.0 (C-16), 23.3 (C-15), 19.2 (C-19), 15.7 (C-25), 8.0 (C-21); ESI-HRMS (m/z): $[M+H]^+$ calcd for $C_{25}H_{40}NO_2$: 386.30536, found 386.30560.

3.9 (−)-*exo*-C-*nor*-D-*homo*-aminoalcohol **9**

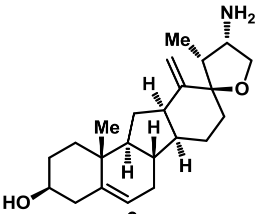

9

To a solution of alcohol **27** (4.4 mg, 11.6 µmol) in EtOH (1 mL) sodiumborohydride (2.4 mg, 63.8 µmol) was added in one portion. The reaction mixture was stirred for 5 d at 65 °C and was allowed to cool to room temperature. Saturated aqueous NH_4Cl solution (2 mL) was added, the aqueous phase was extracted with CH_2Cl_2 (5 x 2 mL), the combined organic layers were dried (Na_2SO_4) and all volatiles were removed under reduced pressure. Column chromatography (SiO_2; $CHCl_3$/EtOH/NH_3 (25% aq.), 95:5:0.5 *v*/*v*/*v*) yielded pure aminoalcohol **9** (2.6 mg, 7.2 µmol, 62%) as a colorless solid; mp: 166-168°C; TLC ($CHCl_3$/EtOH/NH_3 (25% aq.), 90:10:0.5 *v*/*v*/*v*): R_F = 0.21; $[\alpha]_D^{24}$ (deg cm^3 g^{-1} dm^{-1}) = –6 (c = 0.0012 g cm^{-3} in CH_2Cl_2); IR (KBr) ν_{max}: 3445, 2927, 1634, 1384, 1050, 697 cm^{-1}; ^{1}H NMR (300 MHz, CD_3OD) δ 5.35 (s, 1 H), 5.18 (s, 1 H), 4.98 (s, 1 H), 4.15 (q, *J* = 9 Hz, 1 H), 3.85 (m, 1 H), 3.72 (m, 1 H), 3.37 (m, 2 H), 3.24 (m, 1 H), 2.79 (m, 1 H), 2.54 (m, 1 H), 2.31 (m, 1 H), 2.19 (m, 1 H), 2.11 (m, 1 H), 1.86 (m, 1 H), 1.81 (m, 1 H), 1.78 (m, 1 H), 1.75 (m, 1 H), 1.71 (m, 1 H), 1.66 (m, 1 H), 1.63 (m, 1 H), 1.58 (m, 1 H), 1.55 (m, 1 H), 1.50 (m, 1 H), 1.46 (m, 1 H), 1.40 (m, 1 H), 1.38 (m, 1 H), 1.36 (m, 1 H), 1.21 (m, 1 H), 1.18 (m, 1 H), 1.13 (m, 1 H), 1.08 (d, *J* = 7.2 Hz, 3 H), 1.00 (s, 3 H); ESI-HRMS (m/z): $[M+H]^+$ calcd for $C_{23}H_{36}NO_2$: 358.27406, found 358.27391.

3.10 (−)-Alkene **11** and **epi-11**

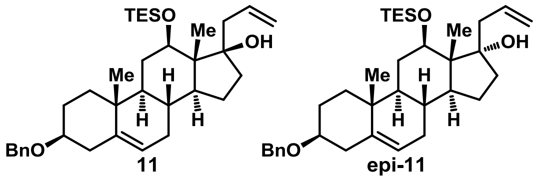

11 epi-11

Ceriumchloride heptahydrate (35.2 g, 94.5 mmol) was heated under vacuum (0.5 mbar) at 150 °C for 3 h then at 0.05 mbar and 300 °C overnight. After cooling to room temperature THF (144 mL) was added and the suspension was stirred for 2 h at room temperature and then cooled to 0 °C. A solution of allylmagnesium bromide (1 M in THF; 94.5 mL, 94.5 mmol,) was added dropwise and the reaction mixture was stirred at this temperature for 1.5 h. Then, 3-benzyl-12β-(triethylsilyloxy)-dehydroandrosterone [1] (4.79 g, 9.41 mmol) in THF (17 mL) was added at once and stirring was continued for another 30 min. The reaction was quenched with saturated aqueous NH_4Cl (200 mL), extracted with EtOAc (2 x 70 mL) and the combined organic extracts were washed with brine (130 mL) and dried ($MgSO_4$). The solvents were removed under reduced pressure and the crude product was purified by column chromatography (SiO_2; *n*-hexane/EtOAc, 22:1 *v*/*v*) to yield a mixture of the epimeric alcohols **11** and **epi-11** in a ration of 15:1 (4.84 g, 8.78 mmol, 93%) as a colorless oil and recovered **10** (335 mg, 0.66 mmol); TLC (*n*-hexane/EtOAc, 10:1 *v*/*v*): 0.38; $[\alpha]^D_{22}$ (deg cm^3 g^{-1} dm^{-1}) = −50 (c = 0.0100 g cm^{-3} in $CHCl_3$); IR (CCl_4): ν_{max} 3574, 732, 697 cm^{-1}; NMR data of the main isomer: ^{1}H NMR (400 MHz, $CDCl_3$) δ 7.30 (m, 5 H), 6.05 (dddd, *J* = 17.1, 10.1, 8.4, 5.5 Hz, 1

H), 5.36 (m, 1 H), 5.15 (d, J = 9.6 Hz, 1 H), 5.10 (d, J = 17.2 Hz, 1 H), 4.57 (d, J = 2.6 Hz, 2 H), 3.85 (dd, J = 11.0, 4.7 Hz, 1 H), 3.28 (tt, J = 11.2, 4.5 Hz, 1 H), 2.46 (m, 2 H), 2.26 (m, 2 H), 1.05 (s, 3 H), 0.99 (t, J = 7.9 Hz, 9 H), 0.95 (s, 3 H), 0.62 (m, 6 H); ^{13}C NMR (100 MHz, $CDCl_3$) δ 140.8, 139.1, 135.6, 128.5, 127.6, 127.5, 121.5, 117.5, 83.2, 78.5, 74.9, 70.1, 50.7, 49.8, 49.6, 41.9, 39.1, 37.5, 37.1, 32.4, 32.0, 31.5, 30.4, 28.6, 23.7, 19.5, 9.6, 7.2, 5.9; ESI-HRMS (m/z): $[M+Na]^+$ calcd for $C_{35}H_{54}O_3SiNa$: 537.37344, found: 537.37288, $[2M+Na]^+$ calcd for $C_{70}H_{108}O_6Si_2Na$: 1123.75767, found: 1123.75776.

3.11 (−)-Diol **28** and **epi-28**

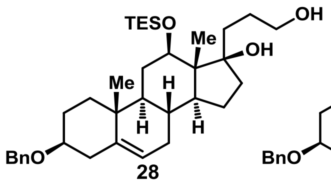

28

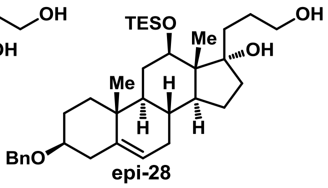

epi-28

To a stirred solution of **11** and **epi-11** (5.07 g, 9.20 mmol) in THF (40 mL) was added a solution of 9-borabicyclo[3.3.1]nonane (0.5 M in THF; 73.6 mL, 36.8 mmol) dropwise at room temperature. After stirring for 6 h at 70 °C the reaction mixture was cooled to 0 °C and H_2O (45 mL) was added carefully. Then sodium perborate (21.23 g, 138.0 mmol) was added sequentially and stirring was continued at 50 °C for 12 h. The reaction was diluted with H_2O (43 mL), extracted with EtOAc (3 x 90 mL) and the combined organic extracts were washed with brine (60 mL) and dried ($MgSO_4$). All volatiles were removed under reduced pressure and the crude product was purified by column chromatography (SiO_2; *n*-hexane/EtOAc, 4:1 *v*/*v*) to yield a mixture of the epimeric alcohols **28** and **epi-28** in a ratio of 15:1 as a colorless oil (4.78 g, 8.41 mmol, 91%); TLC (*n*-hexane/EtOAc, 3:2 *v*/*v*): R_F = 0.42; $[\alpha]_D^{22}$ (deg cm^3 g^{-1} dm^{-1}) = –56 (c = 0.0101 g cm^{-3} in $CHCl_3$); IR (CCl_4): ν_{max} 3573, 3424, 744, 697 cm^{-1}; NMR data of the main isomer: ^{1}H NMR (300 MHz, $CDCl_3$) δ 7.30 (m, 5 H), 5.34 (m, 1 H), 4.56 (d, J = 1.5 Hz, 2 H), 3.87 (dd, J = 10.9, 4.8 Hz, 1 H), 3.67 (t, J = 5.8 Hz, 2 H), 3.27 (m, 1 H), 2.44 (m, 1 H), 2.25 (m, 1 H), 1.04 (s, 3 H), 0.97 (m, 9 H), 0.93 (s, 3 H), 0.61 (m, 6 H); ^{13}C NMR (75 MHz, $CDCl_3$) δ 140.8, 139.1, 128.5, 127.7, 127.6, 121.5, 83.6, 78.5, 74.8, 70.1, 63.9, 50.9, 49.6, 49.6, 39.1, 37.5, 37.2, 33.6, 32.1, 32.1, 31.6, 30.3, 28.6, 27.4, 23.8, 19.5, 9.6, 7.2, 5.9; ESI-HRMS (m/z): $[M+Na]^+$ calcd for $C_{35}H_{56}O_4SiNa$: 591.38401, found: 591.38366.

3.12 (−)-Spirolactone **12** and (−)-20-epi-spirolactone **epi-12**

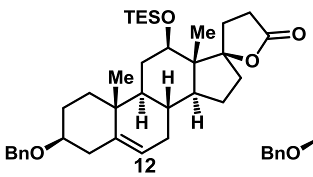

12

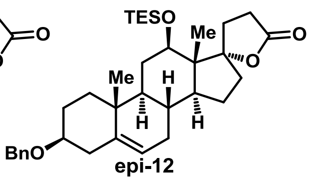

epi-12

To a solution of the alcohols **28** and **epi-28** (4.60 g, 8.07 mmol) in CH_2Cl_2 (60 mL) was added with iodobenzene diacetate (12.8 g, 39.7 mmol) and 2,2,6,6-tetramethylpiperidine-1-oxyl (252 mg, 1.61 mmol) at room temperature and the mixture was stirred for 3 h. The reaction mixture was diluted with CH_2Cl_2 (30 mL) and treated with saturated aqueous $Na_2S_2O_3$ solution (30 mL). The phases were separated and the organic phase was washed with saturated aqueous $NaHCO_3$ solution (20 mL), brine (20 mL), and then dried ($MgSO_4$). All volatiles were removed under reduced pressure and the crude product was purified by column chromatography (SiO_2; *n*-hexane/EtOAc, 9:1 *v*/*v*) to yield pure **12** (3.36 g, 5.94 mmol, 73%) and **epi-12** (250 mg, 0,442 mmol, 5%) both as a colorless foam; **12**: TLC (*n*-hexane/EtOAc, 4:1 v/v): R_F = 0.18; $[\alpha]_D^{22}$ (deg cm^3 g^{-1} dm^{-1}) = –52 (c = 0.0102 g cm^{-3} in $CHCl_3$); IR (CCl_4): ν_{max} 1773, 735, 697 cm^{-1}; ^{1}H NMR (300 MHz, $CDCl_3$) δ 7.34 (m, 4 H, ortho-, meta-H Bn), 7.27 (m, 1 H, para-H Bn), 5.35 (m, 1 H, H-6), 4.56 (s, 2 H, benzyl. H), 3.84 (dd, J = 10.9, 4.7 Hz, 1 H, H-12), 3.28 (tt, J = 11.2, 4.5 Hz, 1 H, H-3), 2.58 (m, 1 H, H-20), 2.49 (m, 1 H, H-20), 2.45 (m, 2 H, H-4, H-21), 2.28 (m, 1 H, H-4), 2.23 (m, 1 H, H-16),

2.03 (m, 1 H, H-7), 1,99 (m, 1 H, H-2), 1.87 (m, 1 H, H-21), 1.81 (m, 1 H, H-1), 1.80 (m, 1 H, H-16), 1.75 (m, 1 H, H-11), 1.66 (m, 1 H, H-15), 1.55 (m, 2 H, H-2, H-11), 1.49 (m, 1 H, H-8), 1.47 (m, 1 H, H-7), 1.45 (m, 1 H, H-15), 1.05 (m, 1 H, H-1), 1.04 (s, 3 H, H-19), 1.02 (m, 1 H, H-9), 1.01 (m, 1 H, H-14), 1.00 (s, 3 H, H-18), 0.97 (t, J = 7.9 Hz, 9 H, CH_3 TES), 0.58 (q, J = 7.9 Hz, 6H, CH_2 TES); ^{13}C NMR (75 MHz, $CDCl_3$) δ 176.3 (C-22), 140.5 (C-5), 138.7 (ipso-C Bn), 128.1 (meta-C Bn), 127.3 (ortho-C Bn), 127.2 (para-C Bn), 120.8 (C-6), 95.5 (C-17), 78.1 (C-3), 74.2 (C-12), 69.7 (benzyl. C), 49.6 (C-14), 49.1 (C-13), 48.9 (C-9), 38.8 (C-4), 37.1 (C-1), 36.6 (C-10), 36.2 (C-16), 31.2 (C-8), 30.9 (C-7), 30.6 (C-11), 30.3 (C-21), 29.2 (C-20), 28.1 (C-2), 22.3 (C-15), 19.2 (C-19), 9.3 (C-18), 6.8 (CH_3 TES), 5.7 (CH_2 TES); ESI-HRMS (m/z): $[M+Na]^+$ calcd for $C_{35}H_{52}O_4SiNa$: 587.35271, found: 587.35270, $[2M+Na]^+$ calcd for $C_{70}H_{104}O_8Si_2Na$: 1151.71619, found: 1151.71706; **epi-12**: TLC (*n*-hexane/EtOAc, 4:1 *v*/*v*): R_F = 0.29; $[\alpha]_D^{22}$ (deg cm^3 g^{-1} dm^{-1}) = –68 (c = 0.0100 g cm^{-3} in $CHCl_3$); IR (CCl_4): ν_{max} 1773, 735, 697 cm^{-1}; ^{1}H NMR (400 MHz, $CDCl_3$) δ 7.30 (m, 5 H), 5.35 (m, 1 H), 4.56 (s, 2 H), 4.05 (dd, J = 11.1, 4.8 Hz, 1 H), 3.29 (tt, J = 11.1, 4.5 Hz, 1 H), 2.77 (m, 1 H), 2.54 (m, 2 H), 2.45 (ddd, J = 13.2, 4.6, 2.2 Hz, 1 H), 2.27 (m, 1 H), 1.03 (s, 3 H), 0.95 (t, J = 7.9 Hz, 9 H), 0.81 (s, 3 H), 0.55 (m, 6 H); ^{13}C NMR (100 MHz, $CDCl_3$) δ 177.2, 140.8, 139.1, 128.5, 127.7, 127.5, 121.5, 97.8, 78.5, 72.8, 70.1, 50.5, 50.3, 49.2, 39.2, 37.9, 37.4, 36.9, 31.5, 30.9, 29.6, 29.0, 28.5, 23.9, 19.5, 9.2, 7.1, 5.7; ESI-HRMS (m/z): $[M+Na]^+$ calcd for $C_{35}H_{52}O_4SiNa$: 587.35271, found: 587.35208, $[2M+Na]^+$ calcd for $C_{70}H_{104}O_8Si_2Na$: 1151.71619, found: 1151.71518.

3.13 (−)-Hydroxylactone **29**

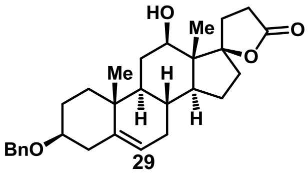

29

To a solution of the lactone **12** (2.80 g, 4.96 mmol) in MeCN (100 mL) was added a solution of hydrofluoric acid (50 wt % in H_2O; 15 mL) in MeCN (100 mL) under stirring at room temperature. After stirring for 20 min the reaction was diluted with EtOAc (100 mL) and saturated aqueous $NaHCO_3$ solution (200 mL) was introduced carefully under vigorous stirring. The phases were separated, the aqueous phase was extracted with EtOAc (4 x 150 mL) and the combined organic extracts were washed with brine (150 mL) and dried ($MgSO_4$). All volatiles were removed under reduced pressure and the crude product was purified by column chromatography (SiO_2; *n*-hexane/EtOAc, 2:1 *v*/*v*) to yield pure **29** (1.95 g, 4.34 mmol, 87%) as a colorless solid; m.p. 215-219°C; TLC (*n*-hexane/EtOAc, 2:1 *v*/*v*): R_F = 0.50; $[\alpha]_D^{22}$ (deg cm^3 g^{-1} dm^{-1}) = –54 (c = 0.0101 g cm^{-3} in $CHCl_3$); IR (KBr): ν_{max} 3489, 1759, 735, 698 cm^{-1}; ^{1}H NMR (400 MHz, $CDCl_3$) δ 7.31 (m, 5 H), 5.34 (m, 1 H), 4.56 (s, 2 H), 3.69 (td, J = 10.7, 5.2 Hz, 1 H), 3.27 (tt, J = 11.2, 4.4 Hz, 1 H), 2.77 (m, 1 H), 2.52 (m, 2 H), 2.44 (ddd, J = 6.7, 5.0, 2.6 Hz, 1 H), 2.28 (m, 2 H), 1.04 (s, 3 H), 0.97 (s, 3 H); ^{13}C NMR (100 MHz, $CDCl_3$) δ 177.3, 141.0, 139.1, 128.5, 127.6, 127.5, 121.0, 95.9, 78.4, 73.2, 70.1, 49.5, 49.5, 49.3, 39.1, 37.3, 37.1, 36.5, 31.5, 31.4, 31.2, 31.0, 29.7, 28.4, 22.6, 19.5, 9.0; ESI-HRMS (m/z): $[M+Na]^+$ calcd for $C_{29}H_{38}O_4Na$: 473.26623, found: 473.26637, $[2M+Na]^+$ calcd for $C_{58}H_{76}O_8Na$: 923,54324, found: 923.54277.

3.14 (−)-*exo*-C-*nor*-D-*homo*-lactone **13** and (−)-*endo* -C-*nor*-D-*homo*-lactone **30**

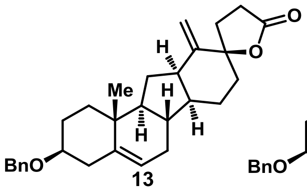

13

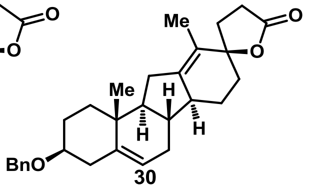

30

To a stirred solution of the alcohol **29** (1.95 g, 4.34 mmol) in pyridine (60 mL) was added triflic anhydride (1.44 mL, 8.68 mmol) at 0 °C. After stirring at this temperature for 10 min the reaction was warmed to 50 °C and kept there for 90 min. The reaction was cooled again to 0 °C,

additional triflic anhydride (1.44 mL, 8.68 mmol) was added and stirring was continued at this temperature for 10 min and thereafter at 50 °C for 2 h. After allowing the dark brown mixture to cool to room temperature, it was diluted with CH_2Cl_2 (200 mL) and washed with hydrochloric acid (0.5 M, 3 x 50 mL) and finally with saturated aqueous $NaHCO_3$ solution (60 mL) and brine (60 mL). The organic phase was dried ($MgSO_4$), all volatiles were removed under reduced pressure and the crude product was purified by column chromatography (SiO_2; *n*-hexane/EtOAc, 8.5:1.5 *v*/*v*) to yield pure *exo*-C-*nor*-D-*homo*-lactone **13** (0.86 g, 2.0 mmol, 46%) and pure *endo*-C-*nor*-D-*homo*-lactone **30** (0.28 g, 0.65 mmol, 15%) both as colorless needles; **13**: m.p. 94-102°C; TLC (*n*-hexane/EtOAc, 2:1 *v*/*v*): R_F = 0.46; $[\alpha]_D^{22}$ (deg cm^3 g^{-1} dm^{-1}) = –32 (c = 0.0100 g cm^{-3} in $CHCl_3$); IR (KBr): ν_{max} 1767, 736, 697 cm^{-1}; ^{1}H NMR (300 MHz, $CDCl_3$) δ 7.30 (m, 5 H), 5.35 (m, 1 H), 5.18 (d, *J* = 2.1 Hz, 1 H), 4.93 (d, *J* = 1.8 Hz, 1 H), 4.56 (s, 2 H), 3.28 (tt, *J* = 11.4, 4.4 Hz, 1 H), 2.74 (m, 1 H), 2.50 (m, 3 H), 2.27 (m, 2 H), 2.09 (m, 4 H), 1.00 (s, 3 H); ^{13}C NMR (75 MHz, $CDCl_3$) δ 177.0, 149.3, 142.2, 139.1, 128.5, 127.6, 127.5, 121.8, 108.2, 88.3, 78.7, 70.0, 53.7, 45.8, 41.2, 38.7, 38.6, 38.3, 37.5, 35.8, 33.1, 31.1, 28.3, 28.3, 26.8, 23.1, 19.0; ESI-HRMS (m/z): $[M+Na]^+$ calcd for $C_{29}H_{36}O_3Na$: 455.25567, found: 455.25541, $[2M+Na]^+$ calcd for $C_{58}H_{72}O_6Na$: 887.52211, found: 887.52285; **30**: m.p. 181-185°C; TLC (*n*-hexane/EtOAc, 2:1 *v*/*v*): R_F = 0.46; $[\alpha]_D^{22}$ (deg cm^3 g^{-1} dm^{-1}) = –110 (c = 0.0098 g cm^{-3} in $CHCl_3$); IR (KBr): ν_{max} 1759, 737, 696 cm^{-1}; ^{1}H NMR (300 MHz, $CDCl_3$) δ 7.30 (m, 5H), 5.36 (m, 1H), 4.57 (s, 2H), 3.29 (tt, *J* = 11.1, 4.3 Hz, 1H), 2.67 (m, 2H), 2.51 (ddd, *J* = 13.3, 4.5, 2.0 Hz, 1H), 2.39 (td, *J* = 13.2, 9.2 Hz, 1H), 2.20 (m, 5H), 1.60 (s, 3H), 0.98 (s, 3H); ^{13}C NMR (75 MHz, $CDCl_3$) δ 177.0, 146.3, 141.9, 139.1, 128.5, 127.7, 127.5, 123.5, 121.7, 86.9, 78.6, 70.1, 52.2, 49.2, 41.9, 38.8, 38.5, 38.3, 37.0, 31.8, 31.1, 30.0, 28.9, 28.2, 24.1, 18.7, 13.2; ESI-HRMS (m/z): $[M+Na]^+$ calcd for $C_{29}H_{36}O_3Na$: 455.25567, found: 455.25609, $[2M+Na]^+$ calcd for $C_{58}H_{72}O_6Na$: 887.52211, found: 887.52275.

3.15 (–)-*exo*-C-*nor*-D-*homo*-azidolactone **14** and (+)-21-epi-*exo*-C-*nor*-D-*homo*-azidolactone **epi-14**

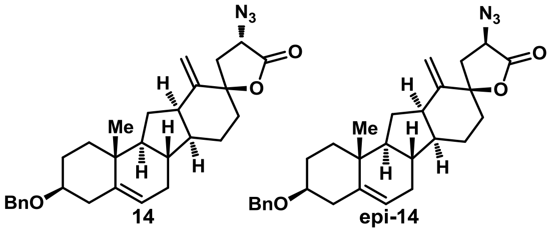

14 epi-14

To a stirred solution of diisopropylamine (938 µL, 6.65 mmol) in THF (6.6 mL) was added *n*-butyllithium (1.6 M solution in hexanes; 4.04 mL, 6.46 mmol) at −78 °C. After stirring for 40 min at this temperature a solution of the lactone **13** (1.60 g, 3.69 mmol) in THF (9 mL) was added dropwise and the temperature was raised to −30 °C over the course of 1 h, kept at this temperature for 10 min and then lowered again to −78 °C. Freshly prepared trisylazide (2.5 g, 8.08 mmol) in THF (13.2 mL) was added in one portion and stirring was continued for 1.5 h. The reaction mixture was quenched with glacial acetic acid (0.66 mL), warmed to room temperature and stirred there for another 30 min. After dilution with EtOAc (75 mL) and saturated aqueous NH_4Cl solution (40 mL), the phases were separated and the aqueous layer was extracted with EtOAc (2 x 75 mL). The combined organic extracts were washed with brine (40 mL), dried ($MgSO_4$) and all volatiles were removed under reduced pressure. Purification by column chromatography (SiO_2; *n*-hexane/EtOAc, 12:1 *v*/*v*) yielded pure **14** (754 mg, 1.59 mmol, 43%) as colorless needles and the epimeric azide **epi-14** (178 mg, 376 µmol, 10%) as a colorless solid; **14**: m.p. 144-146°C; TLC (*n*-hexane/EtOAc, 5:1 *v*/*v*): R_F = 0.46; $[\alpha]_D^{22}$ (deg cm^3 g^{-1} dm^{-1}) = –109 (c = 0.0100 g cm^{-3} in $CHCl_3$); IR (KBr): ν_{max} 2127, 1774, 737, 695 cm^{-1}; ^{1}H NMR (400 MHz, $CDCl_3$) δ 7.31 (m, 5 H), 5.35 (m, 1 H), 5.14 (d, *J* = 2.4 Hz, 1 H), 4.98 (d, *J* = 2.2 Hz, 1 H), 4.57 (s, 2 H), 4.29 (dd, *J* = 10.9, 8.4 Hz, 1 H), 3.28 (tt, *J* = 11.3, 4.4 Hz, 1 H), 2.77 (m, 1 H), 2.71 (dd, *J* = 12.6, 8.4 Hz, 1

H), 2.50 (ddd, J = 13.3, 4.6, 2.1 Hz, 1 H), 2.26 (m, 1 H), 2.10 (m, 2 H), 0.99 (s, 3 H); ^{13}C NMR (100 MHz, $CDCl_3$) δ 173.1, 148.7, 142.1, 139.1, 128.5, 127.6, 127.5, 121.8, 108.8, 86.3, 78.6, 70.1, 57.2, 53.6, 45.9, 41.3, 38.8, 38.7, 38.5, 38.3, 37.5, 36.3, 31.0, 28.2, 26.7, 22.7, 19.0; ESI-HRMS (m/z): $[M+Na]^+$ calcd for $C_{29}H_{35}N_3O_3Na$: 496.25767, found: 496.25726, $[2M+Na]^+$ calcd for $C_{58}H_{70}N_6O_6Na$: 969.52401, found: 969.52406; **epi-14**: m.p. 144-146°C; TLC (*n*-hexane/EtOAc, 5:1 *v*/*v*): R_F = 0.36; $[\alpha]_D^{22}$ (deg cm^3 g^{-1} dm^{-1}) = +50 (c = 0.0102 g cm^{-3} in $CHCl_3$); IR (CCl_4): ν_{max} 2113, 1780, 736, 687 cm^{-1}; ^{1}H NMR (300 MHz, $CDCl_3$) δ 7.31 (m, 5 H), 5.35 (m, 1 H), 5.31 (d, J = 2.3 Hz, 1 H), 4.98 (d, J = 2.2 Hz, 1 H), 4.57 (s, 2 H), 4.39 (t, J = 8.9 Hz, 1 H), 3.28 (tt, J = 11.2, 4.4 Hz, 1 H), 2.72 (m, 1 H), 2.62 (dd, J = 13.1, 8.8 Hz, 1 H), 2.50 (ddd, J = 13.2, 4.6, 2.2 Hz, 1 H), 2.27 (m, 2 H), 2.10 (m, 3 H), 1.00 (s, 3 H); ^{13}C NMR (100 MHz, $CDCl_3$) δ 172.4, 149.1, 142.3, 139.1, 128.5, 127.7, 127.6, 121.8, 108.7, 86.4, 78.7, 70.1, 57.6, 53.7, 45.2, 41.3, 40.0, 39.2, 38.7, 38.3, 37.5, 35.4, 31.2, 28.3, 27.1, 23.6, 19.1; ESI-HRMS (m/z): $[M+Na]^+$ calcd for $C_{29}H_{35}N_3O_3Na$: 496.25732, found: 496.25726, $[2M+Na]^+$ calcd for $C_{58}H_{70}N_6O_6Na$: 969.52449, found: 969.52454.

3.16 (−)-*exo*-C-*nor*-D-*homo*-azidolactol **31**

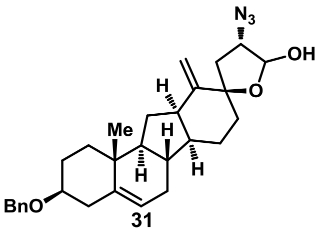

31

To a stirred solution of azidolactone **14** (377 mg, 0.791 mmol) in THF (6.3 mL) at −78 °C was added diisobutylaluminumhydride (1.2 M in toluene; 3.32 mL, 3.98 mmol) dropwise and stirring was continued at this temperature for 1 h and then at −65 °C for 1 h. The reaction mixture was quenched with MeOH (1.6 mL), diluted with CH_2Cl_2 (40 mL) and Rochelle-salt solution (10 wt % in H_2O) (40 mL) and warmed to room temperature. After stirring for 1 h the phases were separated and the aqueous layer was extracted with CH_2Cl_2 (2 x 40 mL). The combined organic extracts were dried ($MgSO_4$) and the solvents removed under reduced pressure. Purification by column chromatography (SiO_2; *n*-hexane/EtOAc, 5:1 *v*/*v*) yielded a mixture of the two epimers of **31** in a 1:1.3 (A:B) ratio (333 mg, 700 µmol, 88%) as a colorless oil: TLC (*n*-hexane/EtOAc, 3:1 *v*/*v*): R_F = 0.46; $[\alpha]_D^{22}$ (deg cm^3 g^{-1} dm^{-1}) = –64 (c = 0.0100 g cm^{-3} in $CHCl_3$); IR (CCl_4): ν_{max} 2105, 734, 697 cm^{-1}; ^{1}H NMR: (400 MHz, $CDCl_3$) δ (ppm) 7.34 (m, 4H, ortho-, meta-H Bn), 7.28 (m, 1H, para-H Bn), 5.46 (m, 1H), 5.36 (m, 1H), 5.34 (m, 1H, H_A), 5.17 (m, 1H, H_B), 4.87 (m, 1H), 4.57 (s, 2H, benzyl. H Bn), 3.99 (ddd, J = 6.5, 4.9, 2.5 Hz, 1H, H_A), 3.76 (m, 1H), 3.67 (ddd, J = 11.2, 7.1, 4.2 Hz, 1H, H_B), 3.56 (m, 1H), 3.29 (m, 1H), 2.72 (m, 1H), 2.50 (m, 1H), 2.28 (m, 2H), 2.11 (m, 2H), 1.00 (s, 3H, H_A-19), 0.98 (s, 3H, H_B-19); ^{13}C NMR: (100 MHz, $CDCl_3$) δ (ppm) 153.2, 151.9, 142.2(A), 142.1(B), 139.0, 128.5, 127.7(A), 127.5(B), 122.0(A), 121.9(B), 107.7(A), 107.4(B), 100.8, 96.2, 88.0, 86.2, 78.72(A), 78.69(B), 70.0, 68.1, 66.3, 61.0, 53.7(A), 53.6(B), 45.7(A), 45.6(B), 42.2(A), 41.6(B), 39.4, 38.7(A), 38.6(B), 38.31(A), 38.27(B), 38.1, 37.6, 37.5, 36.9, 31.2, 30.9, 28.2, 26.8(A), 26.6(B), 25.7, 24.1, 23.4, 19.08(A), 19.06(B); ESI-HRMS (m/z): $[M+Na]^+$ calcd for $C_{29}H_{37}N_3O_3Na$: 498.27271, found: 498.27272, $[2M+Na]^+$ calcd for $C_{58}H_{74}N_6O_6Na$: 973.55621, found: 973.55694.

3.17 (−)-Furan **15**

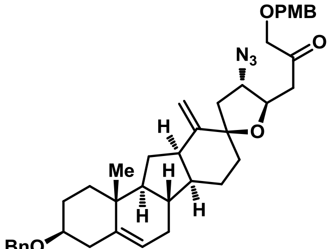

15

Finely ground bariumhydroxide octahydrate (3.83 g, 12.1 mmol) was activated in a constant stream of dry argon at 140 °C while stirring for 2 h. After cooling to room temperature, a solution of dimethyl (3-((4-methoxybenzyl)oxy)-2-oxopropyl)phosphonate [1] (8.24 g, 27.2 mmol) in THF (78 mL) was added and the suspension was warmed to 65 °C under vigorous stirring until a yellow

solution was formed (2 h). The reaction mixture was allowed to cool to room temperature and the lactol **31** (1.44 g, 3.03 mmol) in THF/H_2O (78 mL, 40:1) was introduced in one portion. The reaction mixture was heated to reflux and THF was evaporated slowly overnight (by inserting a fine cannula through a septum, oil bath temperature 80 °C). The remaining viscous oil was dissolved in EtOAc (100 mL), washed with saturated aqueous NH_4Cl (40 mL) and the phases were separated. The aqueous phase was extracted with EtOAc (50 mL) and the combined organic phases were washed with brine (50 mL), dried ($MgSO_4$) and the solvent was removed under reduced pressure. Purification by column chromatography (SiO_2; *n*-hexane/EtOAc, 7:1 *v*/*v*) yielded pure **15** (995 mg, 1.53 mmol, 50%) as a yellow oil; TLC (*n*-hexane/EtOAc, 5:1 *v*/*v*): R_F = 0.17; $[\alpha]_D^{23}$ (deg cm^3 g^{-1} dm^{-1}) = –6 (c = 0.0100 g cm^{-3} in $CHCl_3$); IR (CCl_4): ν_{max} = 2932, 2857, 2103, 1727, 1513, 1250, 1095, 788 cm^{-1}; ^{1}H NMR (300 MHz, $CDCl_3$) δ 7.35 (m, 4 H), 7.27 (m, 3 H), 6.88 (m, 2 H), 5.35 (m, 1 H), 5.25 (s, 1 H), 4.84 (s, 1 H), 4.57 (s, 2 H), 4.53 (s, 2 H), 4.18 (m, 1 H), 4.10 (s, 2 H), 3.80 (s, 3 H), 3.70 (m, 1 H), 3.28 (tt, *J* = 11.2, 4.4 Hz, 1 H), 2.85 (m, 1 H), 2.70 (m, 2 H), 2.49 (ddd, *J* = 13.4, 4.9, 2.4 Hz, 1 H), 2.28 (m, 2 H), 2.12 (m, 1 H), 0.98 (s, 3 H); ^{13}C NMR (100 MHz, $CDCl_3$) δ 206.4, 159.6 (para-C PMB), 152.9, 142.1, 139.1 (ipso-C Bn), 129.8 (ortho-C PMB), 129.3 (ipso-C PMB), 128.5 (meta-C Bn), 127.7 (ortho-C Bn), 127.5 (para-C Bn), 122.0, 114.0 (meta-C PMB), 107.4, 85.6, 78.8, 75.3, 73.2, 70.1, 64.2, 55.4, 53.7, 46.1, 43.0, 42.0, 41.9, 38.7, 38.4, 38.3, 37.5, 36.7, 31.1, 28.3, 26.8, 23.7, 19.1; ESI-HRMS (*m/z*): $[M+H]^+$ calcd for $C_{40}H_{50}N_3O_5$: 652.37505, found: 652.37476, $[M+Na]^+$ calcd for $C_{40}H_{49}N_3O_5Na$: 674,35644, found: 674.35639, $[2M+Na]^+$ calcd for $C_{80}H_{98}N_6O_{10}Na$: 1325.72366, found: 1325.72395.

3.18 (−)-Alkene **16**

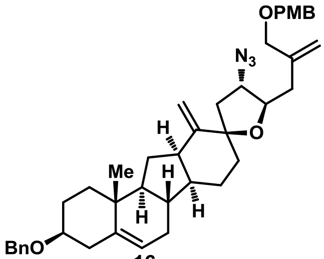

16

Ceriumchloride heptahydrate (2.28 g, 6.00 mmol) was heated under vacuum (0.5 mbar) at 150 °C for 1 h, and then at 0.05 mbar and 300 °C for 12 h. After cooling to room temperature THF (30 mL) was added and the suspension was stirred for 2 h. The thick suspension was cooled to −78 °C and (trimethylsilyl)methyl lithium (1 M in *n*-pentane; 6.00 mL, 6.00 mmol) was added dropwise. The resulting brown suspension was stirred for another 30 min. To this suspension azide **15** (50.0 mg, 76.7 µmol) in THF (1.00 mL) was added dropwise, and after stirring for 50 min TMEDA (0.91 ml, 6.00 mmol) was added. After another 15 min saturated aqueous $NaHCO_3$ (50 mL) was introduced and the mixture was allowed to warm to room temperature and diluted with EtOAc (50 mL). The phases were separated and the aqueous layer was extracted with EtOAc (50 mL). The combined organic extracts were washed with brine (30 mL), dried ($MgSO_4$) and the solvents were removed under reduced pressure. Purification by column chromatography (SiO_2; *n*-hexane/EtOAc, 7:1 *v*/*v*) yielded a mixture of epimeric alcohols (50.0 mg, 67.6 µmol) as colourless oil. These alcohols were dissolved in MeCN (2.0 mL) and under stirring a solution of hydrofluoric acid (50% in water, 1 drop) in MeCN (1.0 mL) was added in one portion. After stirring for 7 min, the reaction was quenched with saturated aqueous $NaHCO_3$ solution (10 mL) and diluted with EtOAc (20 mL). The phases were separated and the aqueous layer was extracted with EtOAc (20 mL). The combined organic extracts were washed with brine (10 mL), dried ($MgSO_4$) and the solvents were removed under reduced pressure. Purification by column chromatography (SiO_2; *n*-hexane/EtOAc, 10:1 *v*/*v*) yielded alkene **16** (32.0 mg, 49.2 µmol, 64%) as a yellow oil; TLC (*n*-hexane/EtOAc, 5:1 *v*/*v*): R_F = 0.41; $[\alpha]_D^{23}$ (deg cm^3 g^{-1} dm^{-1}) = –7 (c = 0.0100 g cm^{-3} in $CHCl_3$); IR (CCl_4): ν_{max} = 2930, 2854, 2101, 1613, 1513 cm^{-1}; ^{1}H NMR (400 MHz, $CDCl_3$) δ 7.34 (m, 4 H, ortho-H Bn, meta-H Bn), 7.26 (m, 3 H,

ortho-H PMB, para-H Bn), 6.87 (m, 2 H, meta-H PMB), 5.33 (m, 2 H), 5.18 (s, 1 H), 5.09 (s, 1 H), 4.84 (s, 1 H), 4.57 (s, 2 H, benzyl. H Bn), 4.44 (s, 2 H, benzyl. H PMB), 4.01 (s, 2 H), 3.95 (m, 1 H), 3.80 (s, 3 H), 3.64 (m, 1 H), 3.28 (m, 1 H), 2.70 (m, 1 H), 2.49 (m, 1 H), 2.44 (m, 2 H), 2.30 (m, 1 H), 2.24 (m, 1 H), 2.11 (m, 1H), 0.99 (s, 3H); ^{13}C NMR (100 MHz, $CDCl_3$) δ 159.3 (para-C PMB), 153.2, 142.6, 142.2, 139.2 (ipso-C Bn), 130.5 (ipso-C PMB), 129.5 (ortho-C PMB), 128.5 (meta-C Bn), 127.7 (ortho-C Bn), 127.5 (para-C Bn), 122.0, 114.6, 113.9 (meta-C PMB), 107.3, 85.2, 80.1, 78.8, 73.0, 71.8 (benzyl. C PMB), 70.1 (benzyl. C Bn), 64.8, 55.4, 53.8, 46.1, 42.2, 42.0, 38.7, 38.5, 38.4, 37.9, 37.6, 36.8, 31.2, 28.3, 26.9, 23.8, 19.1; ESI-HRMS (*m/z*): $[M+Na]^+$ calcd for $C_{41}H_{51}N_3O_4Na$: 672.37718, found: 672.37747.

3.19 (−)-Sulfonamide **17**

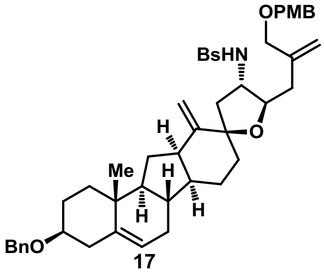

17

To a suspension of lithium aluminumhydride (7.50 mg, 198 µmol) in THF (2.0 mL) a solution of alkene **16** (40.0 mg, 61.6 µmol) in THF (1.0 mL) was added dropwise at 0 °C. The reaction mixture was stirred overnight at room temperature. The reaction was diluted with Et_2O (5 mL) and pH 7 phosphate buffer (5 mL). The phases were separated and the aqueous layer was extracted with Et_2O (2 x 10 mL). The combined organic extracts were dried ($MgSO_4$) and the solvent was removed under reduced pressure. Purification by column chromatography (SiO_2; CH_2Cl_2/MeOH, 3:0.1 *v*/*v*) yielded the corresponding amine (37.0 mg, 59.3 µmol, 97%) as a colorless oil; TLC (CH_2Cl_2/MeOH, 3:0.2 *v*/*v*): R_F = 0.42; ESI-HRMS (*m/z*): $[M+H]^+$ calcd for $C_{41}H_{54}NO_4S$: 624.40474, found: 624.40494. The amine was dissolved in DMF (6.0 mL) and cooled to 0 °C. Triethylamine (132 µL, 948 µmol) and benzenesulfonyl chloride (90.0 µL, 711 µmol) were added sequentially. The yellow solution was stirred at 0 °C for 25 min. The reaction mixture was quenched with saturated aqueous NH_4Cl (60 mL) and diluted with CH_2Cl_2 (10 mL). The phases were separated and the aqueous layer was extracted with CH_2Cl_2 (4 x 10 mL). The combined organic extracts were washed with brine (10 mL), dried ($MgSO_4$) and the solvent was removed under reduced pressure. Purification by column chromatography (SiO_2; *n*-hexane/EtOAc, 10:1 → 2:1 *v*/*v*) yielded sulfonamide **17** (31.0 mg, 40.5 µmol, 69%) as a yellow oil; TLC (*n*-hexane/EtOAc, 2:1 *v*/*v*) R_F = 0.42; $[\alpha]_D^{25}$ (deg cm^3 g^{-1} dm^{-1}) = –3 (c = 0.0100 g cm^{-3} in MeOH);IR (CCl_4): ν_{max} = 3387, 3279, 2931, 2858, 1723, 1650, 1585, 1513, 1448 1163, 1092, 904, 755, 593 cm^{-1}; ^{1}H NMR (400 MHz, $CDCl_3$) δ 7.78 (m, 2 H), 7.55 (m, 1 H), 7.47 (m, 2 H), 7.34 (m, 4 H, ortho-H Bn, meta-H Bn), 7.27 (m, 3 H, ortho-H PMB, para-H Bn), 6.91 (m, 2 H, meta-H PMB), 5.34 (m, 2 H), 5.22 (s, 1 H), 4.83 (s, 1 H), 4.79 (s, 1 H), 4.71 (s, 1 H), 4.56 (s, 2 H, benzyl. H Bn), 4.45 (m, 2 H, benzyl. H PMB), 3.84 (m, 2 H), 3.82 (s, 3 H), 3.31-3.25 (m, 2 H), 2.63 (m, 1 H), 2.47 (m, 1 H), 2.27 (m, 4 H), 2.07 (m, 1 H), 0.96 (s, 3 H), 0.86 (m, 4 H); ^{13}C NMR (75 MHz, $CDCl_3$) δ 159.5 (para-C PMB), 153.2, 142.2, 141.5, 140.4, 139.2 (ipso-C Bn), 132.8, 132.7, 129.9, 129.1, 128.5, 127.7, 127.5, 127.3, 122.0, 114.0, 113.9, 107.1, 85.2, 79.1, 78.8, 73.2, 72.4, 70.1, 57.4, 55.4, 53.7, 45.9, 44.6, 41.9, 38.8, 38.5, 38.4, 38.1, 37.6, 37.2, 31.2, 28.3, 26.9, 23.7, 19.1; ESI-HRMS (*m/z*): $[M+Na]^+$ calcd for $C_{47}H_{57}NO_6SNa$: 786.37988, found: 786.37998.

3.20 (+)-Allylic alcohol **32**

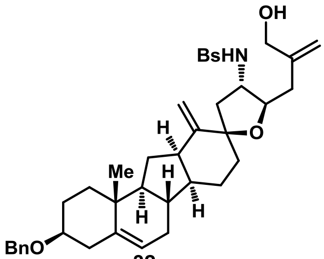

32

To a solution of the sulfonamide **17** (56.0 mg, 73.3 µmol) in CH_2Cl_2 (2.6 mL) was added pH 7 phosphate buffer (0.26 mL) and 2,3-dichloro-5,6-dicyano-1,4-benzoquinone (16.0 mg, 73.3 µmol), after stirring at room temperature for 1 h, another portion of 2,3-dichloro-5,6-dicyano-1,4-benzoquinone (16.0 mg, 73.3 µmol) was added and stirring was continued for 1 h. The reaction mixture was then diluted with CH_2Cl_2 (10 mL), washed with saturated aqueous $NaHCO_3$ (10 mL) and the aqueous phase was extracted with CH_2Cl_2 (2 x 10 mL). After drying ($MgSO_4$) all volatiles were removed under reduced pressure and the crude product was purified by column chromatography (SiO_2; *n*-hexane/EtOAc, 1:1 *v*/*v*) to yield **32** (28.0 mg, 43.5 µmol, 59%) as a colorless oil; TLC (*n*-hexane/EtOAc, 1:1 *v*/*v*) R_F = 0.44; $[\alpha]_D^{23}$ (deg cm^3 g^{-1} dm^{-1}) = +3 (c = 0.0100 g cm^{-3} in $CHCl_3$); IR (CCl_4): ν_{max} = 3445, 3276, 2928, 2358, 1652, 1447, 1330, 1162, 1093, 903, 755, 593 cm^{-1}; ^1H NMR (400 MHz, $CDCl_3$) δ 7.88 (m, 1 H, ortho-H Bs), 7.85 (m, 1 H, ortho-H Bs), 7.61 (m, 1 H, para-H Bs), 7.53 (m, 2 H, meta-H Bs), 7.34 (m, 4 H, ortho-H Bn, meta-H Bn), 7.28 (m, 1 H, para-H Bn), 5.32 (m, 1 H), 5.22 (s, 1 H), 5.02 (m, 1 H), 4.97 (s, 1 H), 4.83 (s, 1 H), 4.78 (s, 1 H), 4.56 (s, 2 H), 4.04 (s, 2 H), 3.77 (td, *J* = 8.3, 4.2 Hz, 1 H), 3.41 (m, 1 H), 3.26 (tt, *J* = 11.2, 4.5 Hz, 1 H), 2.68 (br, 1 H), 2.56 (m, 2 H), 2.48 (ddd, *J* = 13.3, 4.8, 2.2 Hz, 1 H), 2.26 (m, 4 H), 2.08 (m, 1 H), 0.97 (s, 3 H); ^{13}C NMR (75 MHz, $CDCl_3$) δ 152.8, 145.3, 142.2, 140.5, 139.2 (ipso-C Bn), 133.0, 129.3, 128.5 (meta-C Bn), 127.7 (ortho-C Bn), 127.5, 127.2, 121.9, 114.9, 107.7, 85.4, 80.6, 78.8, 70.1, 66.6, 57.3, 53.7, 46.1, 44.2, 41.7, 38.7, 38.5, 38.4, 37.9, 37.5, 37.3, 31.1, 28.3, 26.9, 23.6, 19.1; ESI-HRMS (*m/z*): $[M+Na]^+$ calcd for $C_{39}H_{49}NO_5SNa$: 666.32237, found: 666.32295.

3.21 (−)-*N*-Bs-*O*-Bn-20-demethyl-bis-*exo*-cyclopamine **18**

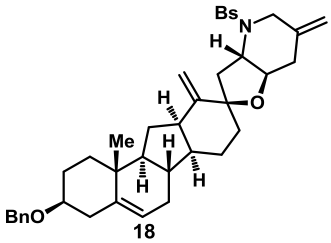

18

To a solution of the allylic alcohol **32** (28.0 mg, 43.5 µmol) in toluene (3.0 mL) was added tributylphosphine (28.0 µL, 113 µmol) and 1,1'-(azodicarbonyl)-dipiperidine (28.0 mg, 104 µmol) at 0 °C. After warming to room temperature, stirring was continued for another 12 h. The reaction mixture was evaporated to dryness and the crude product was purified by column chromatography (SiO_2; *n*-hexane/EtOAc, 7:1 *v*/*v*) to yield pure **18** (25.0 mg, 39.9 µmol, 93%) as a colorless oil; TLC (*n*-hexane/EtOAc, 3:1 *v*/*v*) R_F = 0.55; $[\alpha]_D^{24}$ (deg cm^3 g^{-1} dm^{-1}) = –3 (c = 0.0094 g cm^{-3} in $CHCl_3$); IR (KBr): ν_{max} = 3442, 2927, 2857, 2666, 2359, 1637, 1449, 1354 cm^{-1}; ^1H NMR (300 MHz, $CDCl_3$) δ 7.76 (m, 2 H), 7.58 (m,3 H), 7.35 (m, 5 H), 5.35 (m, 1 H, H-6), 5.17 (s, 1 H), 5.09 (s, 1 H), 5.02 (s, 1 H), 4.86 (s, 1 H), 4.57 (s, 2 H), 4.16 (d, *J* = 12.3 Hz, 1 H), 3.53 (m, 1 H), 3.29 (m, 1 H, H-3), 2.78 (m, 2 H), 2.58 (m, 1 H), 2.50 (m, 1 H), 2.26 (m, 3 H), 0.90 (s, 3 H); ^{13}C NMR (100 MHz, $CDCl_3$) δ 153.6 (C-13), 142.1 (C-5), 139.2 (ipso-C Bn), 137.6, 135.2 (ipso-C Bs), 133.3 (para-C Bs), 129.3 (meta-C Bs), 128.5 (meta-C Bn), 128.1 (ortho-C Bs), 127.7 (ortho-C Bn), 127.6 (para-C Bn), 122.1 (C-6), 116.1 (C-27), 108.3 (C-18), 85.7 (C-17), 78.8 (C-3), 78.6 (C-23), 70.1 (benzyl. C Bn), 61.0 (C-22), 55.9, 53.6, 46.7 (C-14), 42.6, 41.1 (C-12), 38.8 (C-4), 38.4 (C-1), 38.2 (C-8), 37.5, 37.4, 31.0 (C-7), 29.9, 28.4 (C-2), 27.0 (C-11), 23.1 (C-15), 19.1 (C-19); HRMS (*m/z*): $[M+Na]^+$ calcd for $C_{39}H_{47}NO_4SNa$: 648.31180, found: 648.31193.

3.22 (–)-*N*-Bs-20-demethyl-bis-*exo*-cyclopamine **33**

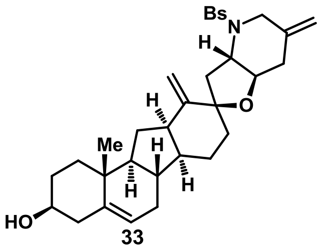

To a solution of *N*-Bs-*O*-Bn-20-demethyl-bis-*exo*-cyclopamine **18** (18.0 mg, 28.8 µmol) in 1,2-dichloroethane (2.3 ml) and pH 7 phosphate buffer (0.24 mL) 2,3-dichloro-5,6-dicyano-1,4-benzoquinone (52.0 mg, 229 µmol) was added in one portion at 44 °C. After stirring at this temperature for 50 min the reaction mixture was quenched with saturated aqueous $NaHCO_3$ solution (5 mL) and the aqueous phase was extracted with CH_2Cl_2 (2 x 5 mL). After drying ($MgSO_4$) all volatiles were removed under reduced pressure and the crude product was purified by column chromatography (SiO_2; *n*-hexane/EtOAc, 7:1 → 1:1 *v*/*v*) yielding pure **33** (6.1 mg, 9.9 µmol, 34%) as a yellow oil and starting material **18** (8.5 mg, 12.1 µmol). The reisolated starting material was subjected twice to the above procedure for an overall yield of **33** of 10.8 mg, 20.2 µmol, 70%; TLC (*n*-hexane/EtOAc, 1:1 *v*/*v*) R_F = 0.32; $[\alpha]_D^{26}$ (deg cm^3 g^{-1} dm^{-1}) = –13 (c = 0.0104 g cm^{-3} in $CHCl_3$); IR (CCl_4): ν_{max} = 3388, 2925, 2855, 2360, 2341, 1732, 1635, 1445, 1385, 1171, 908, 787, 600 cm^{-1}; ^1H NMR (400 MHz, $CDCl_3$) δ 7.76 (m, 2 H), 7.58 (m, 3 H), 5.36 (m, 1 H), 5.17 (s, 1 H), 5.09 (s, 1 H), 5.02 (s, 1 H), 4.86 (s, 1 H), 4.16 (d, *J* = 12.2 Hz, 1 H), 3.55 (m, 2 H), 2.78 (m, 3 H), 2.57 (m, 1 H), 2.36 (m, 1 H), 2.24 (m, 3 H), 2.07 (m, 2 H), 0.96 (s, 3 H, H-19); ^{13}C NMR (100 MHz, $CDCl_3$) δ 153.7 (C-13), 142.0 (C-5), 137.7, 133.4 (para-C Bs), 129.4 (meta-C Bs), 128.1 (ortho-C Bs), 127.3, 122.3 (C-6), 116.2 (C-27), 108.3 (C-18), 85.7 (C-17), 78.7 (C-23), 72.1 (C-3), 61.1 (C-22), 54.7, 53.7, 46.8 (C-14), 42.6, 42.0, 41.2 (C-12), 38.4 (C-1), 38.3 (C-8), 37.5, 37.2, 31.6, 31.0 (C-7), 29.9, 27.1 (C-11), 23.1 (C-15), 19.1 (C-19); ESI-HRMS (*m/z*): $[M+H]^+$ calcd for $C_{32}H_{42}NO_4S$: 536.28291, found: 536.28294, $[M+Na]^+$ calcd for $C_{32}H_{41}NO_4SNa$: 558.26485, found: 558.26470, $[2M+Na]^+$ calcd for $C_{64}H_{82}N_2O_8S_2Na$: 1093.54048 found: 1093.54091.

3.23 (−)-20-Demethyl-bis-*exo*-cyclopamine **19**

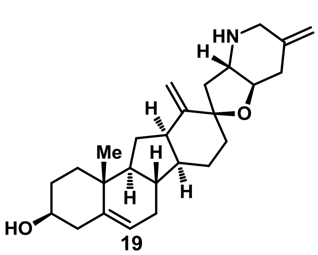

O-Bn-20-demethyl-bis-*exo*-cyclopamine **33** (10.0 mg, 18.7 µmol) was dissolved in 1,2-dimethoxyethane (2.0 ml) and the solution was cooled to −78 °C. Under stirring sodium naphthalenide (0.5 M in 1,2-dimethoxyethane) (125 µL, 62.5 µmol) (freshly prepared by adding sodium (5.70 mg, 0.25 mmol) to a solution of naphthalene (38.5 mg, 0.30 mmol) in 1,2-dimethoxyethane (0.50 mL) and stirring for 1 h until a deep green solution had formed) was added in one portion. After stirring for 30 min saturated aqueous $NaHCO_3$ solution (5 ml) was added and after warming to room temperature the mixture was extracted with CH_2Cl_2 (5 x 5 mL) and dried (Na_2SO_4). The solvents were removed under reduced pressure and the resulting crude was purified by column chromatography ($CHCl_3$/EtOH/NH_3 (25% aq.), 99:1:0.5, *v*/*v*/*v*) to yield **19** (7.00 mg, 9.59 µmol, 95%); TLC ($CHCl_3$/EtOH/NH_3 (25% aq.), 99:1:0.5, *v*/*v*/*v*), R_F = 0.26; $[\alpha]_D^{23}$ (deg cm^3 g^{-1} dm^{-1}) = –17 (c = 0.0079 g cm^{-3} in $CHCl_3$); IR (CCl_4): ν_{max} = 3433, 2923, 2852, 1742, 1646, 1384 cm^{-1}; ^1H NMR (400 MHz, $CDCl_3$) δ 5.35 (m, 2 H, H-18, H-6), 5.02 (m, 2 H, H- 27, H-27), 4.93 (s, 1 H, H-18), 3.62 (d, *J* = 13.8 Hz, 1 H, H-26), 3.52 (tt, *J* = 10.8, 4.4 Hz, 1H), 3.44 (ddd, *J* = 11.5, 9.4, 4.2 Hz, 1H, H-23), 3.38 (d, *J* = 13.8 Hz, 1 H, H-26), 2.89 (dd, *J* = 12.2, 4.3 Hz, 1 H, H-24), 2.80 (m, 1 H, H-22), 2.74 (m, 1 H, H-12), 2.37 (m, 2 H, H-24, H-4), 2.30 (dd, *J* = 11.4, 6.5 Hz, 1H, H-20), 2.20 (m, 1 H, H-4), 2.11 (m, 1 H, H-7), 1.85 (m, 3 H, H-2, H-11, H-16), 1.73 (m, 5 H, H-20, H-16, H-11, H-15, H-1), 1.61 (m, 2 H, H-14, H-7), 1.51 (m, 2 H, H-15, H-2), 1.39 (m, 1 H, H-8), 1.33 (m, 1 H, H-9), 1.16 (m, 1 H, H-1), 0.97 (s, 3H, H-19); ^{13}C NMR (100 MHz, $CDCl_3$) δ 153.4 (C-13), 142.1 (C-5), 140.2 (C-25), 122.1 (C-6), 115.4

(C-27), 108.6 (C-18), 85.9 (C-17), 78.9 (C-23), 72.0 (C-3), 60.6 (C-22), 53.7 (C-9), 52.5 (C-26), 46.7 (C-14), 42.0 (C-4), 41.3 (C-12), 40.9 (C-20), 39.4 (C-24), 38.4 (C-1), 38.2 (C-8), 37.4 (C-10), 37.2 (C-16), 31.6 (C-2), 31.0 (C-7), 26.9 (C-11), 23.1 (C-15), 19.1 (C-19); ESI-HRMS (*m/z*): $[M+H]^+$ calcd for $C_{26}H_{38}NO_2$: 396.28971, found: 396.28950.

3.24 (−)-Furan **20**

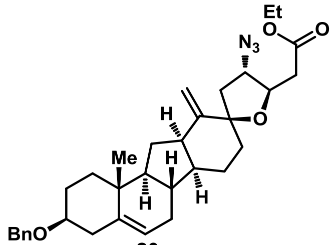

20

Finely ground bariumhydroxide octahydrate (336 mg, 1.06 mmol) was activated in a constant stream of dry argon at 140 °C. The powder was allowed to cool to room temperature and after adding a solution of triethyl phosphonoacetate (537 mg, 2.40 mmol) in THF (7.0 mL), the suspension was warmed to 60 °C under vigorous stirring for 45 min and thereafter sonicated for 10 min at room temperature. A solution of lactol **31** (126 mg, 0.266 mmol) in THF/H_2O (7.0 mL, 40:1) was introduced in one portion and after sonicating for 10 min, the reaction mixture was brought to reflux and THF was evaporated slowly over the course of 12 h (by inserting a fine cannula through a septum, oil bath temperature 80 °C). The remaining viscous orange oil was dissolved in EtOAc (30 mL), washed with saturated aqueous NH_4Cl solution (15 mL) and the phases were separated. The aqueous phase was extracted with EtOAc (15 mL) and the combined organic phases were washed with brine (15 mL), dried ($MgSO_4$) and all volatiles were removed under reduced pressure. Column chromatography (SiO_2; *n*-hexane/EtOAc, 7:1 *v*/*v*) yielded pure **20** (80.3 mg, 0.147 mmol, 55%) as a colorless oil; TLC (*n*-hexane/EtOAc, 6:1 *v*/*v*): R_F = 0.43; $[\alpha]_D^{22}$ (deg cm^3 g^{-1} dm^{-1}) = –15 (c = 0.0100 g cm^{-3} in $CHCl_3$); IR (CCl_4): ν_{max} 2103, 1736, 735, 697 cm^{-1}; ^{1}H NMR (400 MHz, $CDCl_3$) δ 7.35 (m, 2 H, ortho-H Bn), 7.33 (m, 2 H, meta-H Bn), 7.28 (m, 1 H, para-H Bn), 5.35 (m, 1 H, H-6), 5.28 (m, 1 H, H-18), 4.85 (m, 1 H, H-18), 4.56 (s, 2 H, benzyl. H), 4.18 (m, 1 H, H-22), 4.17 (q, *J* = 7.1 Hz, 2 H, CH_2 Et), 3.82 (q, *J* = 7.2 Hz, 1 H, H-21), 3.28 (tt, *J* = 11.1, 4.5 Hz, 1 H, H-3), 2.68 (m, 1 H, H-12), 2.65 (m, 2 H, H-23), 2.49 (ddd, *J* = 13.1, 4.6, 2.0 Hz, 1 H, H-4), 2.31 (m, 1 H, H-20), 2.25 (m, 1 H, H-4), 2.11 (m, 1 H, H-7), 1.99 (m, 1 H, H-20), 1.95 (m, 1 H, H-2), 1.81 (m, 1 H, H-11), 1.77 (m, 1 H, H-16), 1.76 (m, 1 H, H-1), 1.74 (m, 1 H, H-16), 1.72 (m, 1 H, H-11), 1.65 (m, 1 H, H-15), 1.60 (m, 2 H, H-7, H-14), 1.56 (m, 1 H, H-2), 1.51 (m, 1 H, H-15), 1.36 (m, 1 H, H-8), 1.33 (m, 1 H, H-9), 1.28 (t, *J* = 7.1 Hz, 3 H, CH_3 Et), 1.12 (dt, *J* = 13.6, 3.6 Hz, 1 H, H-1), 0.98 (s, 3 H, H-19); ^{13}C NMR (175 MHz, $CDCl_3$) δ 170.5 (C-24), 152.9 (C-13), 142.2 (C-5), 139.2 (ipso-C Bn), 128.5 (meta-C Bn), 127.7 (ortho-C Bn), 127.5 (para-C Bn), 122.0 (C-6), 107.4 (C-18), 85.6 (C-17), 78.8 (C-3), 78.0 (C-22), 70.1 (benzyl. C Bn), 64.1 (C-21), 60.9 (CH_2 Et), 53.7 (C-9), 46.1 (C-14), 42.1 (C-20), 41.9 (C-12), 38.8 (C-23), 38.7 (C-4), 38.4 (C-8), 38.3 (C-1), 37.5 (C-10), 36.7 (C-16), 31.1 (C-7), 28.3 (C-2), 26.8 (C-11), 23.7 (C-15), 19.1 (C-19), 14.3 (CH_3 Et); HRMS (m/z): $[M+Na]^+$ calcd for $C_{33}H_{43}N_3O_4Na$: 568.31567, found: 568.31524, $[2M+Na]^+$ calcd for $C_{66}H_{86}N_6O_8Na$: 1113.63698, found: 1113.63753.

3.25 (+)-Sulfonamide **21**

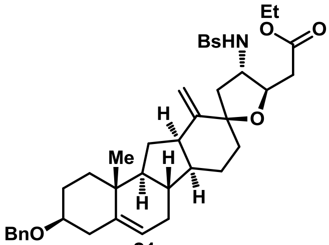

21

A solution of the furan **20** (103.0 mg, 155.0 µmol) in THF (7 mL) was treated sequentially with H_2O (34 µL, 0.189 mmol) and triphenylphosphine (28.8 mg, 110.0 µmol) under stirring at room temperature. The solution was warmed to 50 °C and kept there for 24 h. After allowing it to cool to room temperature, all volatiles were evaporated and the crude amine was azeotroped with THF (10 mL) two times. After drying at 0.01 mbar pressure for 10 h, the crude amine was dissolved in CH_2Cl_2 (6 mL) and cooled to 0 °C. Triethylamine (126 µL, 786 µmol) and benzenesulfonyl chloride (97 µL, 755 µmol) were added sequentially and the mixture was heated to reflux for 5 h. The solution was cooled to room temperature, diluted with CH_2Cl_2 (20 mL) and washed with saturated aqueous NH_4Cl solution (10 mL). The phases were separated and the aqueous layer was extracted with CH_2Cl_2 (10 mL). The combined organic extracts were washed with brine (10 mL), dried ($MgSO_4$) and the volatiles were removed under reduced pressure. Column chromatography (SiO_2; *n*-hexane/EtOAc, 3:1 *v*/*v*) yielded pure **21** (86 mg, 111 µmol, 97%) as a colorless oil; TLC (*n*-hexane/EtOAc, 3:1 *v*/*v*): $R_F = 0.25$; $[\alpha]_D^{26}$ (deg cm^3 g^{-1} dm^{-1}) = +2 (c = 0.0134 g cm^{-3} in $CHCl_3$); IR (CCl_4): ν_{max} 2924, 2359, 2340, 1632, 1453, 1384, 1164, 1093, 689 cm^{-1}; ^{1}H NMR (300 MHz, $CDCl_3$) δ 7.86 (d, *J* = 6.9 Hz, 2 H), 7.61 (t, *J* = 7.2 Hz, 1 H), 7.51 (t, *J* = 7.2 Hz, 2 H), δ 7.35 (m, 2 H), 7.33 (m, 2 H), 7.28 (m, 1 H), 5.39 (d, *J* = 5.4 Hz, 1 H), 5.34 (m, 1 H), 5.16 (s, 1 H), 4.81 (s, 1 H), 4.56 (s, 2 H), 4.14 (m, 1 H), 4.08 (q, *J* = 7.1 Hz, 2 H), 3.40 (q, *J* = 7.2 Hz, 1 H), 3.28 (tt, *J* = 11.1, 4.5 Hz, 1 H), 2.60 (m, 1 H), 2.65 (m, 2 H), 2.49 (ddd, *J* = 13.1, 4.6, 2.0 Hz, 1 H), 2.31 (m, 1 H), 2.25 (m, 1 H), 2.11 (m, 1 H), 1.12 (dt, *J* = 13.6, 3.6 Hz, 1 H), 0.96 (s, 3 H); ^{13}C NMR (75 MHz, $CDCl_3$) δ 171.7, 152.9, 139.2, 135.2, 133.2, 129.3, 128.5, 128.1, 127.7, 127.6, 122.0, 107.5, 85.4, 78.8, 78.0, 70.1, 61.1, 57.8, 53.7, 46.0, 44.2, 41.8, 38.7, 38.4, 38.3, 37.6, 37.1, 31.1, 28.3, 26.9, 23.6, 19.1, 14.3. ESI-HRMS (m/z): $[M+Na]^+$ calcd for $C_{39}H_{49}NO_6SNa$: 682.31783, found: 682.31728; $[2M+Na]^+$ calcd for $C_{78}H_{98}NO_{12}S_2$: 1341.64589, found: 1341.64534.

3.26 (+)-Alcohol **34**

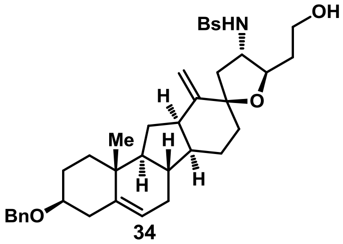

34

To a stirred solution of the sulfonamide **21** (50 mg, 76 µmol) in THF (7 mL) was added diisobutylaluminumhydrid (1.2 M in toluene, 330 µL, 312.8 µmol) dropwise at −78 °C and stirring was continued at this temperature for 1 h and then at −40 °C for 2 h. The reaction mixture was quenched with MeOH (31 µL), diluted with CH_2Cl_2 (20 mL) and Rochelle-salt solution (10 wt % in H_2O, 20 mL) and warmed to room temperature. After stirring for 1 h the phases were separated and the aqueous layer was extracted with CH_2Cl_2 (2 x 20 mL). The combined organic extracts were dried ($MgSO_4$) and the volatiles were removed under reduced pressure. Column chromatography (SiO_2; *n*-hexane/EtOAc, 2:1 *v*/*v*) yielded pure **34** (45.5 mg, 74 µmol, 97%) as a colorless film; TLC (*n*-hexane/EtOAc, 1:1 *v*/*v*): $R_F = 0.27$; $[\alpha]_D^{22}$ (deg cm^3 g^{-1} dm^{-1}) = +4 (c = 0.0099 g cm^{-3} in $CHCl_3$); IR (CCl_4): ν_{max} 3440, 2929, 2102, 1631, 1447, 1343, 1162, 1093, 788, 756 cm^{-1}; ^{1}H NMR (300 MHz, $CDCl_3$): δ 7.86 (d, *J* = 6.9 Hz, 2 H), 7.59 (t, *J* = 7.2 Hz, 1 H), 7.51 (t, *J* = 7.2 Hz, 2 H), 7.34 (m, 2 H), 7.33 (m, 2 H), 7.27 (m, 1 H), 5.50 (d, *J* = 7.4 Hz, 1 H), 5.32 (dd, *J* = 5.3, 2.6 Hz, 1 H), 5.19 (d, *J* = 2.3 Hz, 1 H), 4.81 (s, 1 H), 4.56 (s, 2 H), 3.79 (m, 1 H), 3.72 (t, *J* = 5.4 Hz, 2 H), 3.35 (m, 1 H), 3.27 (ddd, *J* = 11.0, 6.7, 4.4 Hz, 1 H), 2.57 (m, 1 H), 2.48 (ddd, *J* = 13.4, 4.8, 2.2 Hz, 1 H), 2.24 (m, 1 H), 2.20 (dd, *J* = 12.7, 7.4 Hz, 1 H), 1.96 (dt, *J* = 12.9, 3.4 Hz, 1 H), 1.11 (td, *J* = 13.5, 3.7 Hz, 1 H), 0.96 (s, 3 H); ^{13}C NMR (100 MHz, $CDCl_3$) δ 152.9, 142.2, 140.3, 135.2, 133.2, 129.4,

128.5, 128.0, 127.7, 127.6, 122.0, 107.8, 85.5, 81.0, 78.8, 70.1, 67.8, 61.1, 57.6, 53.7, 46.1, 43.8, 41.7, 38.7, 38.4, 37.5, 37.4, 35.0, 31.1, 28.3, 26.9, 23.5, 19.1; ESI-HRMS (m/z): $[M+Na]^+$ calcd for $C_{37}H_{47}NO_5Na$: 640.30726, found: 640.30703.

3.27 (+)-*N*-Bs-*O*-Bn-F-*nor*-20,25-bis-demethyl-*exo*-cyclopamine **22**

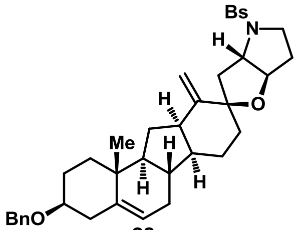

22

To a solution of alcohol **34** (20 mg, 32 µmol) in toluene (1.6 mL) were added sequentially tributylphosphine (34.0 µL, 134 µmol) and diethyl azodicarboxylate (31.4 mg, 123 µmol) at 0 °C. After warming to room temperature, stirring was continued for 24 h. Afterwards all volatiles were removed under reduced pressure. Column chromatography (SiO_2; *n*-hexane/EtOAc, 6:1 v/v) yielded pure **22** (15.4 mg, 25.9 µmol, 81%) as colorless plates; mp.: 133-135°C; TLC (*n*-hexane/EtOAc, 3:1 v/v): R_F = 0.36; $[\alpha]_D^{24}$ (deg cm^3 g^{-1} dm^{-1}) = +50 (c = 0.0099 g cm^{-3} in $CHCl_3$); IR (KBr): ν_{max} 2925, 2884, 1718, 1446, 1347, 1175, 1092, 905, 798, 694, 598 cm^{-1}; ^1H NMR (400 MHz, $CDCl_3$): δ 7.78 (d, *J* = 6.9 Hz, 2 H, ortho-H Bs), 7.64 (t, *J* = 7.2 Hz, 1 H, para-H Bs), 7.56 (t, *J* = 7.2 Hz, 2 H, meta-H Bs), 7.35 (m, 2 H, ortho-H Bn), 7.32 (m, 2 H, meta-H Bn), 7.28 (m, 1 H, para-H Bn), 5.34 (m, 1 H, H-6), 5.20 (s, 1 H, H-18), 4.89 (s, 1 H, H-18), 4.57 (s, 2 H, benzyl. H), 4.09 (m, 1 H, H-22), 3.85 (q, J = 9 Hz, 1 H, H-23), 3.55 (t, *J* = 9 Hz, 1 H, H-23), 3.28 (tt, *J* = 11.1, 4.5 Hz, 1 H, H-3), 2.92 (m, 1H, H-21), 2.76 (m, 1 H, H-12), 2.49 (ddd, *J* = 13.1, 4.6, 2.0 Hz, 1 H, H-4), 2.34 (m, 1 H, H-19), 2.25 (m, 1 H, H-4), 2.08 (m, 1 H, H-7), 2.06 (m, 1 H, H-23), 1.95 (m, 1 H, H-2), 1.93 (m, 1 H, H-19), 1.81 (m, 1 H, H-11), 1.77 (m, 1 H, H-16), 1.76 (m, 1 H, H-1), 1.74 (m, 1 H, H-16), 1.72 (m, 1 H, H-11), 1.68 (m, 1 H, H-22), 1.65 (m, 1 H, H-15), 1.60 (m, 2 H, H-7, H-14), 1.58 (m, 1 H, H-2), 1.51 (m, 1 H, H-15), 1.36 (m, 1 H, H-8), 1.33 (m, 1 H, H-9), 1.12 (dt, *J* = 13.6, 3.6 Hz, 1 H, H-1), 0.96 (s, 3 H, H-19); ^{13}C NMR (100 MHz, $CDCl_3$): δ 154.9 (C-13), 142.2 (C-5), 139.2 (ipso-C Bn), 135.2(ipso-C Bs), 133.2 (para-C Bs), 129.3 (meta-C Bs), 128.1 (ortho-C Bs), 128.5 (meta-C Bn), 127.7 (ortho-C Bn), 127.6 (para-C Bn), 122.0 (C-6), 108.5 (C-18), 96.6 (C-17), 82.9 (C-22), 78.8 (C-3), 70.1 (benzyl. C), 66.3 (C-21), 53.6 (C-9), 53.6 (C-23), 46.5 (C-14), 41.0 (C-12), 40.2 (C-20), 38.8 (C-4), 38.3 (C-19), 38.2 (C-8), 37.7 (C-1), 37.5 (C-10), 31.0 (C-16), 31.0 (C-7), 28.3 (C-2), 26.8 (C-13) ,26.4 (C-11), 22.7 (C-15), 19.2 (C-19); HRMS (m/z): $[M+Na]^+$ calcd for $C_{37}H_{45}NO_4SNa$: 662.29670, found: 622.29614; $[2M+Na]^+$ calcd for $C_{74}H_{90}N_2O_8S_2Na$: 1221.60163, found: 1221.60240.

3.28 (+)-*N*-Bs-F-*nor*-20,25-bis-demethyl-*exo*-cyclopamine **35**

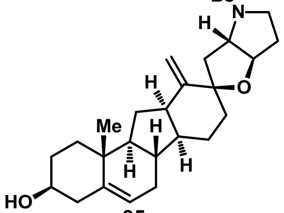

35

To a solution of *N*-Bs-*O*-Bn-F-*nor*-20,25-bis-demethyl-*exo*-cyclopamine **22** (14.4 mg, 24.0 µmol) in 1.9 mL 1,2-dichloroethane and phosphate-buffer (0.2 mL, pH 7) was added 2,3-dichloro-5,6-dicyano-1,4-benzoquinone (43.7 mg, 192.5 µmol) at 40 °C and the reaction was stirred for 30 min and then quenched with saturated aqueous $NaHCO_3$ solution. The aqueous phase was extracted with CH_2Cl_2 (3 x 5 mL), the combined organic layers were dried ($MgSO_4$) and all volatiles were removed under reduced pressure. Column chromatography (SiO_2; *n*-hexane/EtOAc, 6:1 → 3:1 *v/v*) gave pure **35** (5.4 mg, 9.2 µmol, 38%) as colorless crystals and starting material **22** (7.1 mg, 10.4 µmol). The reisolated starting material was resubjected two more times to this reaction to give an overall yield of **35** of 8.9 mg, 14.9 µmol, 62%; mp.: 186-189°C; TLC (*n*-hexane/EtOAc, 2:1 *v/v*): R_F = 0.18; $[\alpha]_D^{22}$(deg cm^3 g^{-1} dm^{-1}) = +164 (c = 0.0082 g cm^{-3} in $CHCl_3$); IR (KBr): ν_{max} 3444, 1635, 1350, 1170, 720, 601 cm^{-1}; ^1H NMR (400

MHz, $CDCl_3$) δ 7.78 (d, *J* = 6.9 Hz, 2 H, ortho-H Bs), 7.63 (t, *J* = 7.2 Hz, 1 H, para-H Bs), 7.56 (t, *J* = 7.2 Hz, 2 H, meta-H Bs), 5.36 (m, 1 H), 5.20 (s, 1 H), 4.89 (s, 1 H), 4.09 (m, 1 H), 3.84 (m, 1 H), 3.55 (m, 1 H), 3.52 (m, 1 H), 2.92 (m, 1H), 2.77 (m, 1 H), 2.37 (ddd, *J* = 13.1, 4.6, 2.0 Hz, 1 H), 2.32 (dd, *J* = 5.6, 11.2 Hz, 1 H), 2.20 (m, 1 H), 2.11 (m, 1 H), 2.04 (m, 1 H), 1.12 (dt, *J* = 13.6, 3.6 Hz, 1 H), 0.97 (s, 3 H); ^{13}C NMR (100 MHz, $CDCl_3$): δ 152.9, 141.9, 135.3 (ipso-C Bs), 133.2 (para-C Bs), 129.3 (meta-C Bs), 128.1 (ortho-C Bs), 122.2, 108.6, 96.6, 82.9, 72.0, 66.3, 53.6, 53.6, 46.6, 41.9, 41.0, 40.2, 38.3, 38.2, 37.7, 37.1, 31.5, 31.0, 26.8 ,26.4, 22.7, 19.2; ESI-HRMS (m/z): $[M+Na]^+$ calcd for $C_{30}H_{39}NO_4SNa$: 532.24920, found: 532.24930.

3.29 (−)-F-*nor*-20,25-bis-demethyl-*exo*-cyclopamine **23**

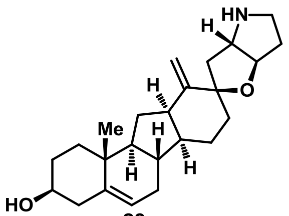

23

To a solution of *N*-Bs-F-*nor*-20,25-bis-demethyl-*exo*-cyclopamine **35** (5.9 mg, 16.1 µmol) in 1,2-dimethoxyethane (1 mL) a freshly prepared sodium naphthalenide solution (0.5 M in 1,2-dimethoxyethane, 250 µL, 125 µmol) was added dropwise at −78 °C. After stirring for 40 min the reaction was quenched with saturated aqueous $NaHCO_3$ solution and the mixture was allowed to warm to room temperature. The aqueous phase was extracted with CH_2Cl_2 (5 x 5 mL). The combined organic extracts were dried (Na_2SO_4), and all volatiles were removed under reduced pressure. Column chromatography (SiO_2; $CHCl_3$/EtOH/NH_3 (25% aq.), 95:2.5:0.5 *v*/*v*/*v*) yielded pure **23** (3.6 mg, 8.2 µmol, 74%) as a colorless solid; m.p.: 205-215°C; TLC ($CHCl_3$/MeOH/NH_3(25% aq.), 95:5:0.5, v/v/v): R_F = 0.14; $[\alpha]_D^{22}$ = –12 (c = 0.0023 g cm^{-3} in $CHCl_3$); IR (KBr) ν_{max}: 3433, 3255, 2925, 1631, 1454, 1399, 1261, 1094, 804, 544 cm^{-1}; ^1H NMR (400 MHz, $CDCl_3$) δ 5.39 (s, 1 H, H-18), 5.34 (m, 1 H, H-6), 4.93 (s, 1 H, H-18), 3.94 (ddd, *J* = 11.3, 9.5, 6.4 Hz, 1 H, H-22), 3.65 (m, 1 H, H-24), 3.55 (m, 1 H, H-24), 3.52 (m, 1 H, H-3), 3.16 (m, 1 H, H-21), 2.72 (m, 1 H, H-12), 2.36 (ddd, *J* = 12.9, 4.9, 2.3 Hz, 1 H, H-4), 2.21 (m, 1 H, H-4), 2.13 (m, 1 H, H-7), 2.10 (m, 1 H, H-20), 1.97 (m, 1 H, H-16), 1.93 (m, 1 H, H-23), 1.88 (m, 1 H, H-11), 1.86 (m, 2 H, H-16, H-2), 1.84 (m, 1 H, H-23), 1.75 (m, 1 H, H-11), 1.74 (m, 1 H, H-1), 1.72 (m, 1 H, H-15), 1.65 (m, 1 H, H-7), 1.63 (m, 1 H, H-20), 1.62 (m, 1 H, H-14), 1.55 (m, 1 H, H-2), 1.47 (m, 1 H, H-15), 1.37 (m, 1 H, H-8), 1.33 (m, 1 H, H-9), 1.17 (dt, *J* = 13.6, 3.7 Hz, 1 H, H-1), 0.98 (s, 3 H, H-19); ^{13}C NMR (100 MHz, $CDCl_3$) δ 153.3 (C-13), 142.0 (C-5), 122.2 (C-6), 108.6 (C-18), 96.7 (C-17), 85.9 (C-22), 72.0 (C-3), 66.4 (C-21), 53.6 (C-9), 51.6 (C-24), 46.8 (C-14), 41.9 (C-4), 41.0 (C-12), 38.3 (C-1), 38.1 (C-8), 37.9 (C-23), 37.1 (C-10), 31.6 (C-2), 31.0 (C-7), 26.7 (C-11), 26.2 (C-16), 22.8 (C-15), 19.0 (C-19); ESI-HRMS (m/z): $[M+H]^+$ calcd for $C_{24}H_{36}NO_2$: 370.27406, found: 370.27396.

4. Biochemistry

The interference of the *exo*-cyclopamine derivatives with the hedgehog signaling pathway was tested in an established reporter gene assay [2] based on the inhibition of the target gene Gli1. Shh-LIGHTII cells (ATCC CRL-2795, LGC, Wesel, Germany) represent a clonal mouse fibroblast cell line (NIH 3T3), which stably incorporates a Gli-dependent firefly luciferase reporter and a constitutive Renilla luciferase reporter. They were grown in 75 cm^3 cell culture flasks at 37 °C in a humid atmosphere with 5% CO_2. Cell growth medium DIMETHOXYETHANEM (Dulbecco's Modified Eagle's Medium, high glucose, sodium pyruvate, w/o glutamine), ZeocinTM Selection Reagent and Geneticin® Selection Antibiotic (G418 sulfate) were obtained from Invitrogen. Additive L-glutamin and trypsin were obtained from PAA. VerseneTM chelating agent was obtained from Gibco, bovine fetal serum (FBS) from Sigma. The cell freeze medium contained 5% DMSO in complete growth medium. The cell number was counted using a Neubauer-Zählkammer (Hemocytometer). DIMETHOXYETHANEM (high glucose, sodium pyruvate, w/o glutamine) supplemented with 0.5% FBS, 4 mM L-glutamin and 50 mM HEPES buffer (4-(2-hydroxyethyl)-1-piperazine ethanesulfonic acid) (pH 7.4) was used as incubation medium. The measurement of reporter gene and constitutive Renilla luminescence was performed with the Dual Luciferase® reporter system according to the manufacturer’s instructions (Promega, Mannheim, Germany) using a GENios reader (TECAN, Crailsheim, Germany).

Luciferase reporter assay.

Incubation of cells.

For performing the assay, Shh-LIGHTII cells were grown to reach 80% confluence, washed twice with Versene, detached with 2 mL trypsin for not longer than 2 min and resuspended in 8 mL of growth medium. After centrifugation for 3 min, the supernatant was removed and the cell pellet dissolved in approximately 10 mL of growth medium. Finally, 20,000 to 100,000 cells per well were cultured in a 24-well plate for 48 h. For exposure to the derivatives the growth medium was removed and the cells were exposed to the compound in 500 µL/well incubation medium for 48 h. Stock solutions were prepared in EtOH and introduced in different concentrations into the incubation medium to reach a final solvent concentration of 0.05%. Due to the assay principle, Shh-LIGHTII cells had to be co-exposed to 100 nM of SAG (Smoothened agonist) for determining the inhibitory activity of the compounds. A SAG stock solution was prepared in EtOH and introduced into the medium. The final EtOH concentration – introduced by SAG and the test compound – was 0.1%. As a positive control a 100 nM SAG solution in incubation medium (0.1% EtOH) was used, negative controls were treated with EtOH only.

Performing the luciferase measurement.

After 48 h of incubation cells were washed with 500 µL PBS (phosphate buffered saline, w Mg/Ca, Invitrogen) and afterwards incubated for 15 min with 100 µL 1 x passive lysis buffer on a shaker. The lysed cells together with lysis solution were transferred into 1.5 mL reaction tubes, centrifuged for 1 min at 4 °C and kept on ice until further use. At first, the blank of luminescence of a 96-well plate (flat bottom, white, Greiner bio-one) was recorded. Then, 100 µL luciferase assay reagent and 10 µL cell supernatant were mixed per well and the luminescence of firefly luciferase was recorded. Addition of 100 µL Stop&Glo® reagent permitted the recording of Renilla luminescence. The measurement was performed per row of a well, i.e. assay reagent was added to

8 wells and luminesescence recorded, before the next row was proceeded. Analysis of constitutive Renilla luminescence was used to normalize for any potential unspecific Gli1-reporter gene luminescence.

Evaluation of IC_{50}.

For determination of IC_{50} values, the relative luminescence units per second RLU/s (relative luminescence units per second, quotient of firefly and Renilla luminescence) were plotted against the inhibitor concentrations on a log_{10} scale. Each test was performed three times with three replicates per concentration.

5. References

[1] Giannis, A.; Heretsch, P.; Sarli, V.; Stößel, A.; *Angew. Chem. Int. Ed.* **2009**, *48 (42)*, 7911–7914.

doi: 10.1002/anie.200902520

[2] Taipale, J.; Chen, J. K.; Cooper, M. K.; Wang, B.; Mann, R. K.; Milenkovic, L.; Scott M. P.; Beachy, P. A. *Nature*, **2000**, *406 (6799)*, 1005–1009.

doi:10.1038/35023008

6. NMR Spectra

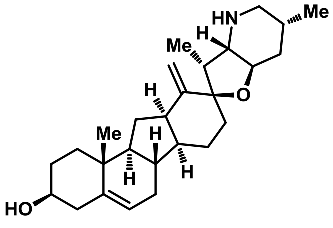

5

^{1}H NMR (300 MHz, $CDCl_3$)

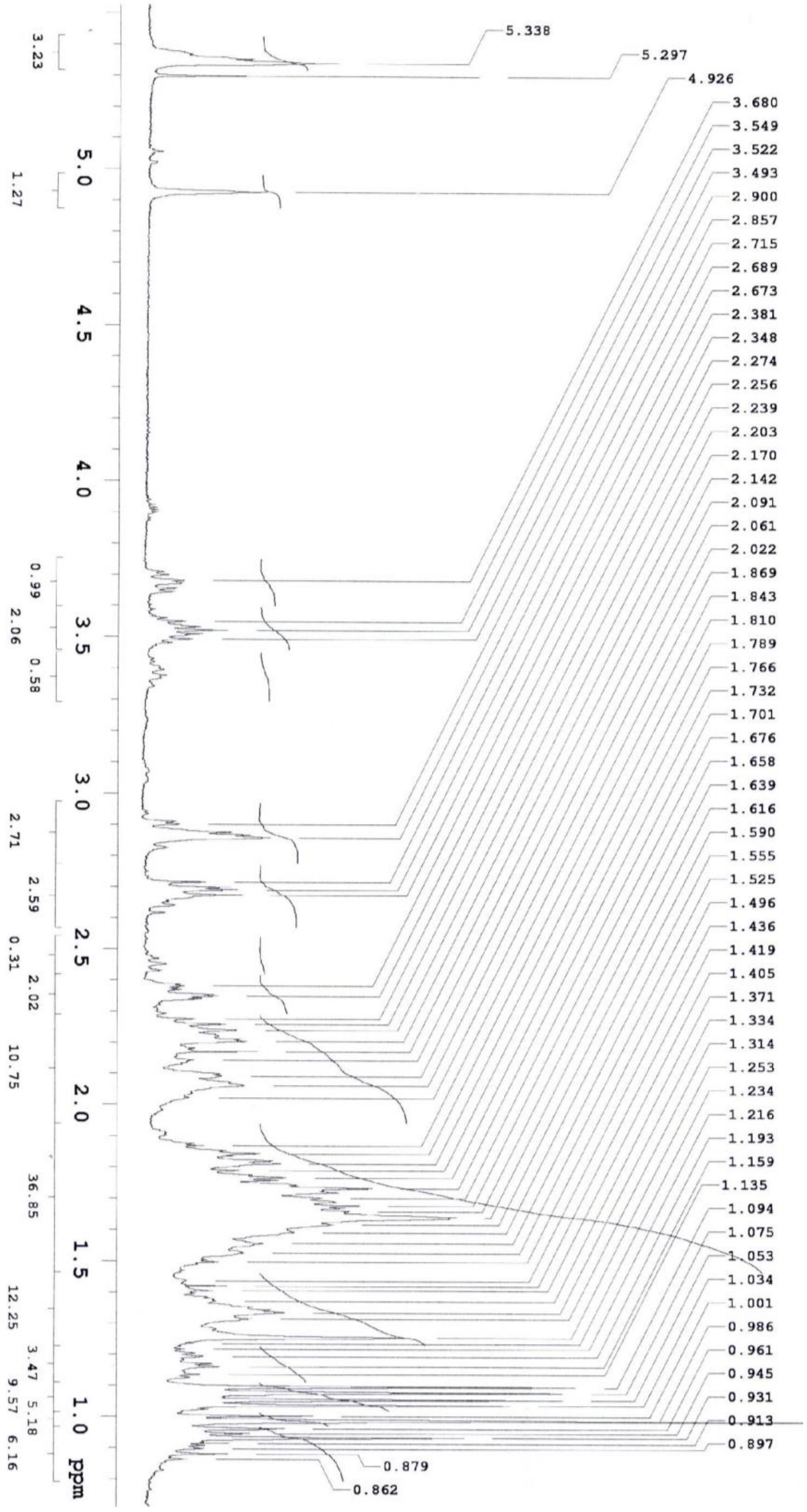

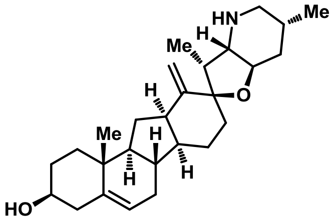

5

^{13}C NMR (100 MHz, $CDCl_3$)

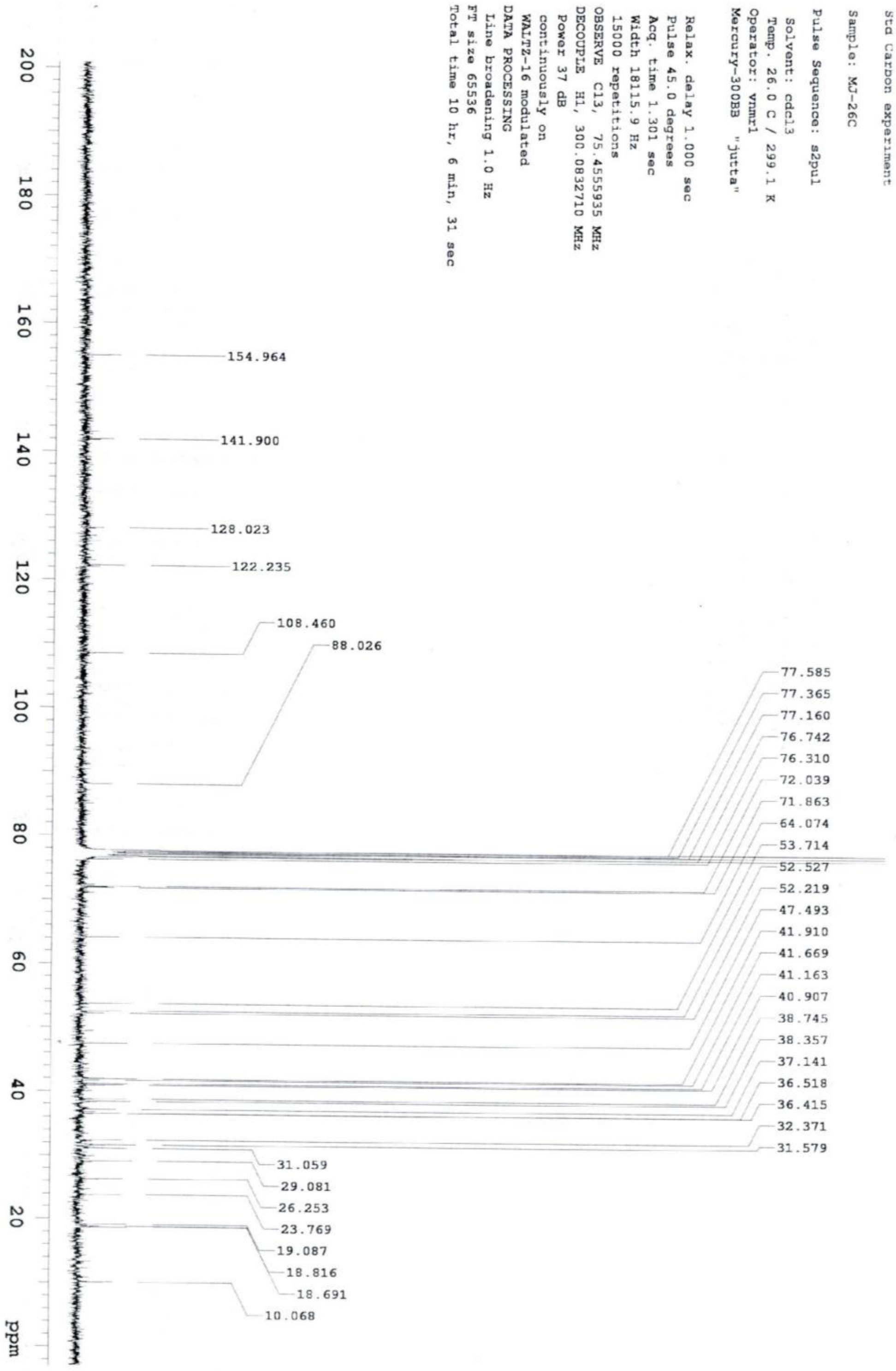

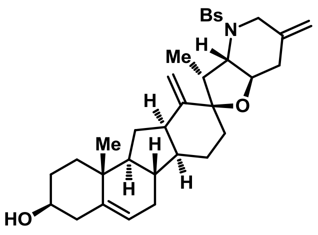

24

^{1}H NMR (300 MHz, $CDCl_3$)

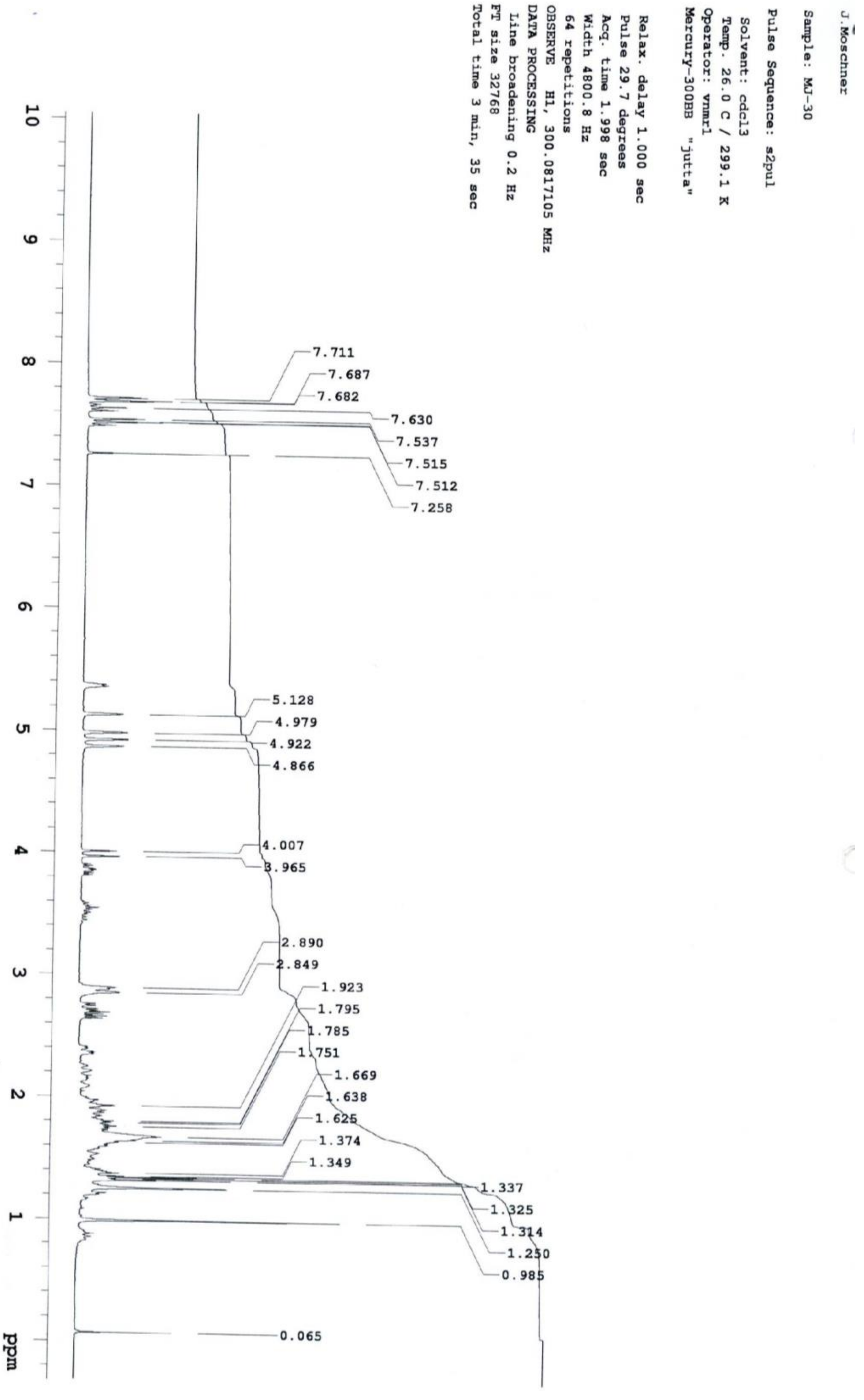

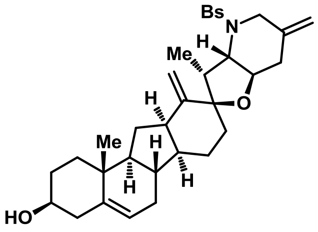

24

^{13}C NMR (100 MHz, CDCl$_3$)

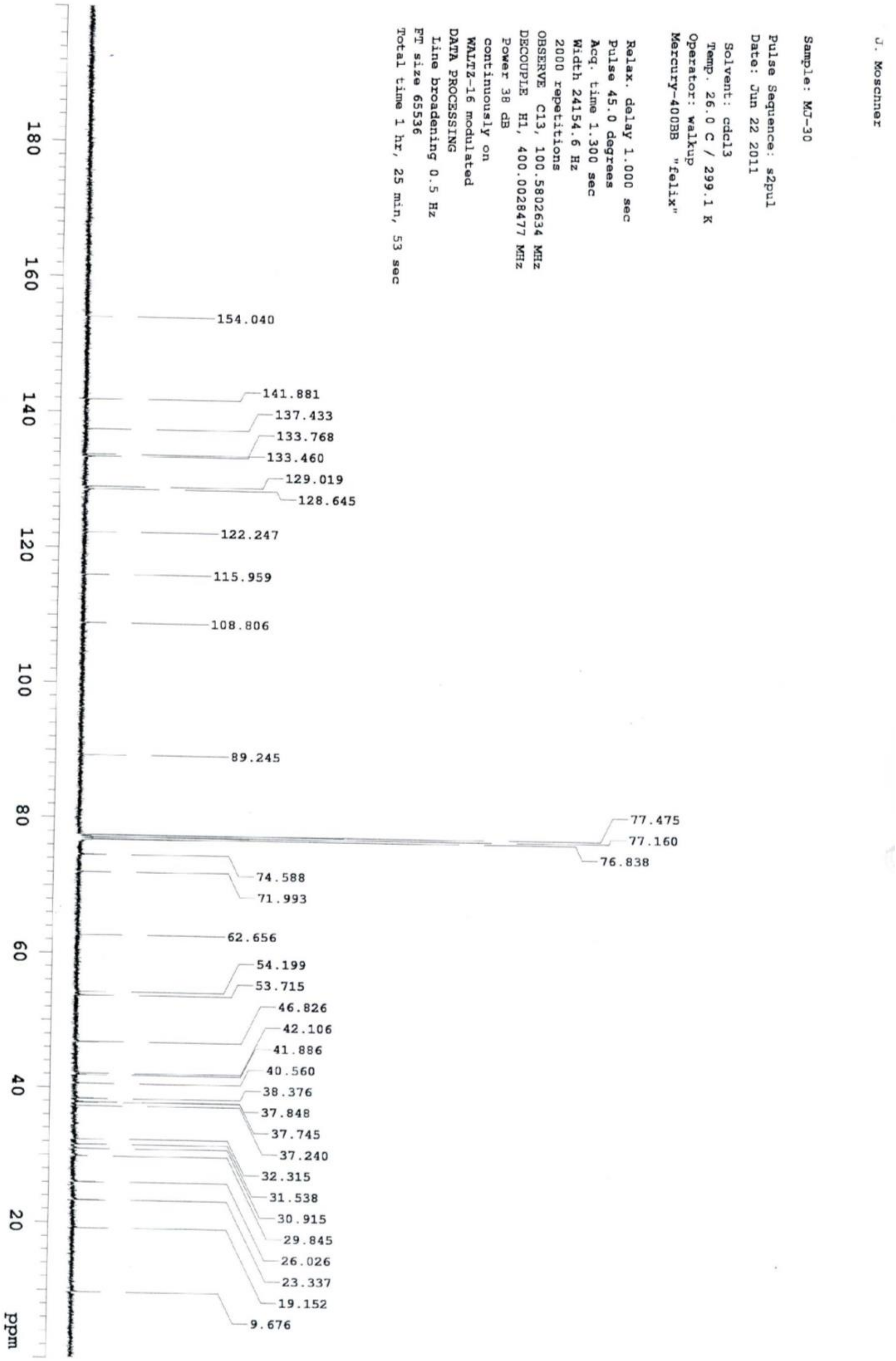

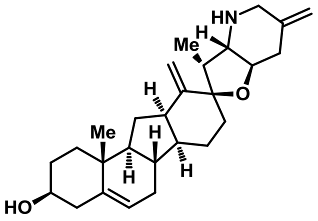

6

^{1}H NMR (300 MHz, $CDCl_3$)

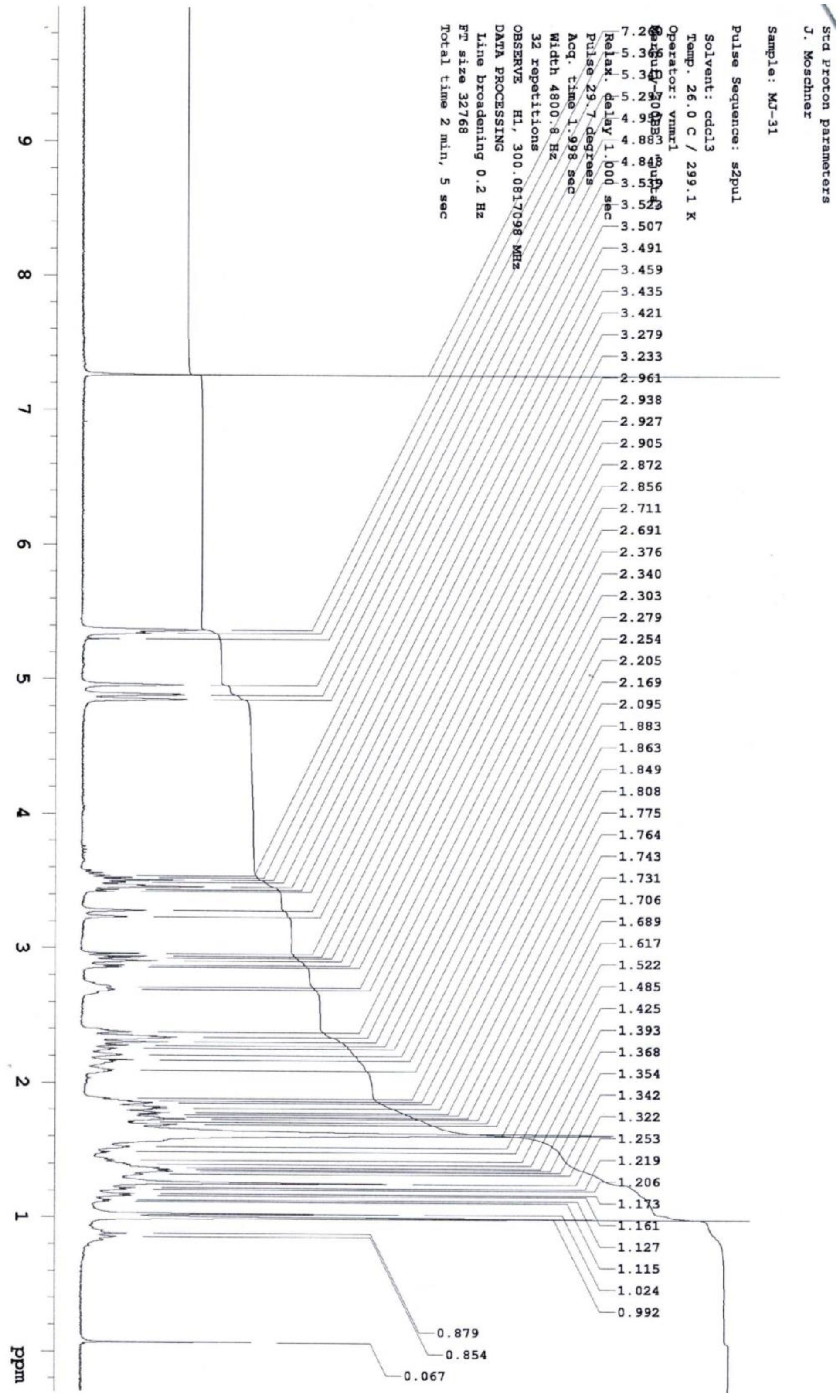

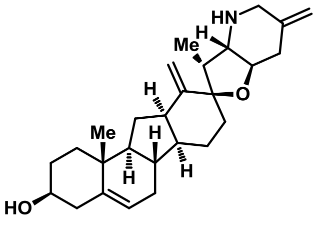

6

APT (100 MHz, $CDCl_3$)

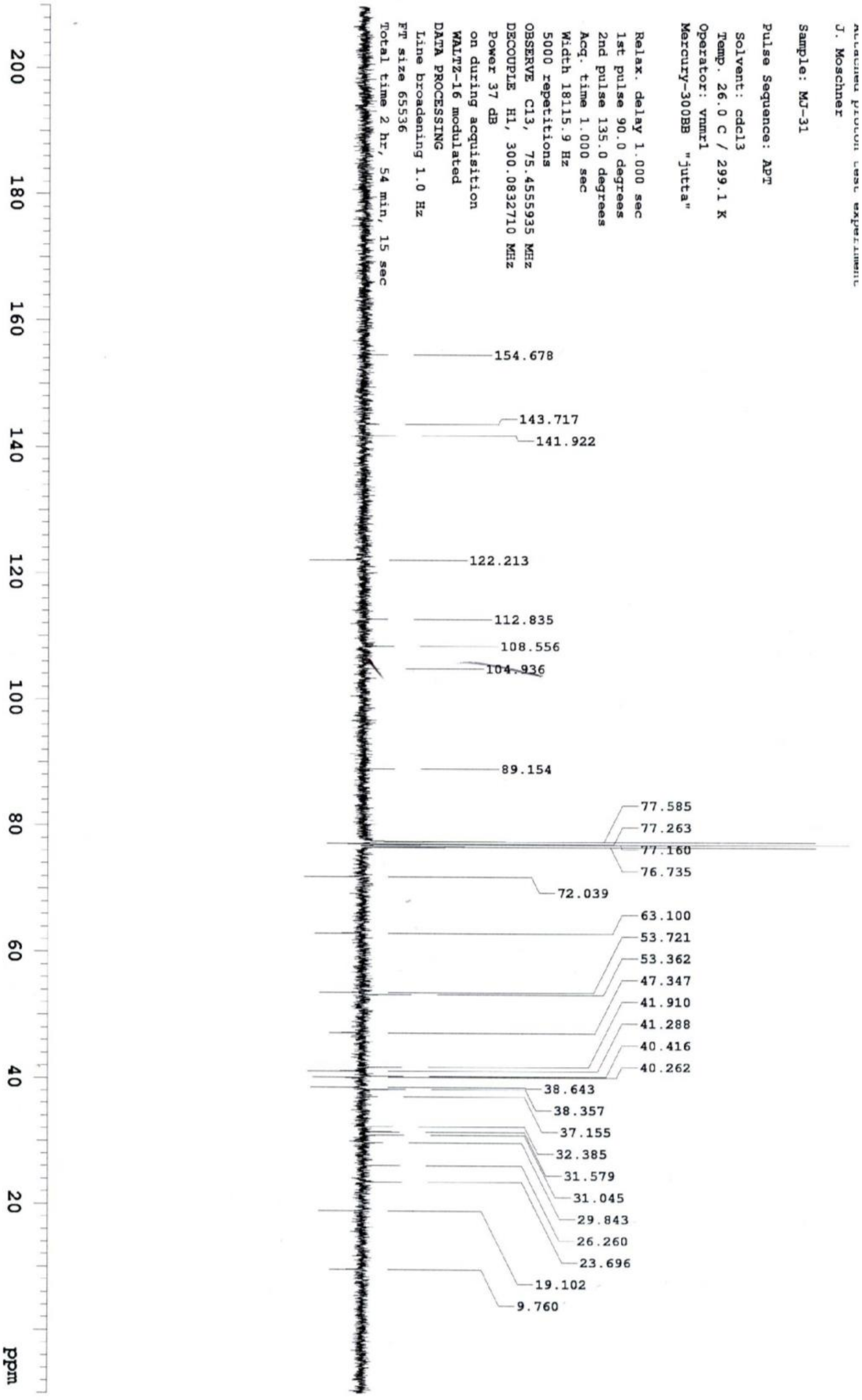

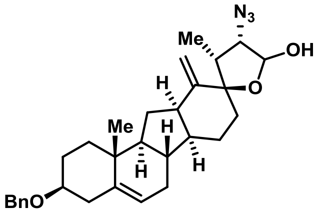

25

^{1}H NMR (300 MHz, $CDCl_3$)

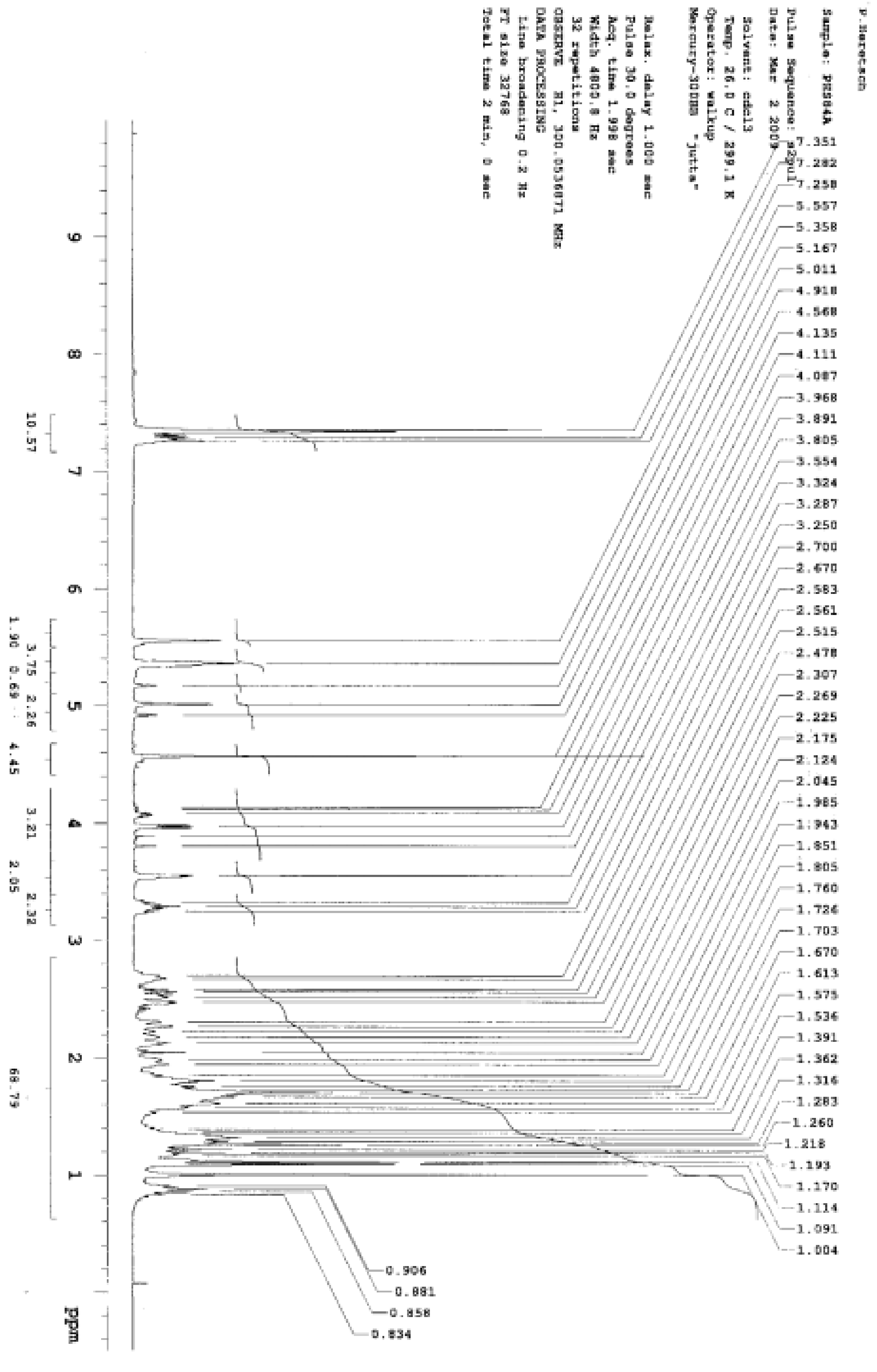

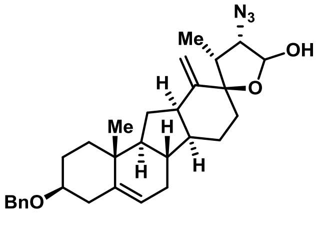

25

^{13}C NMR (75 MHz, $CDCl_3$)

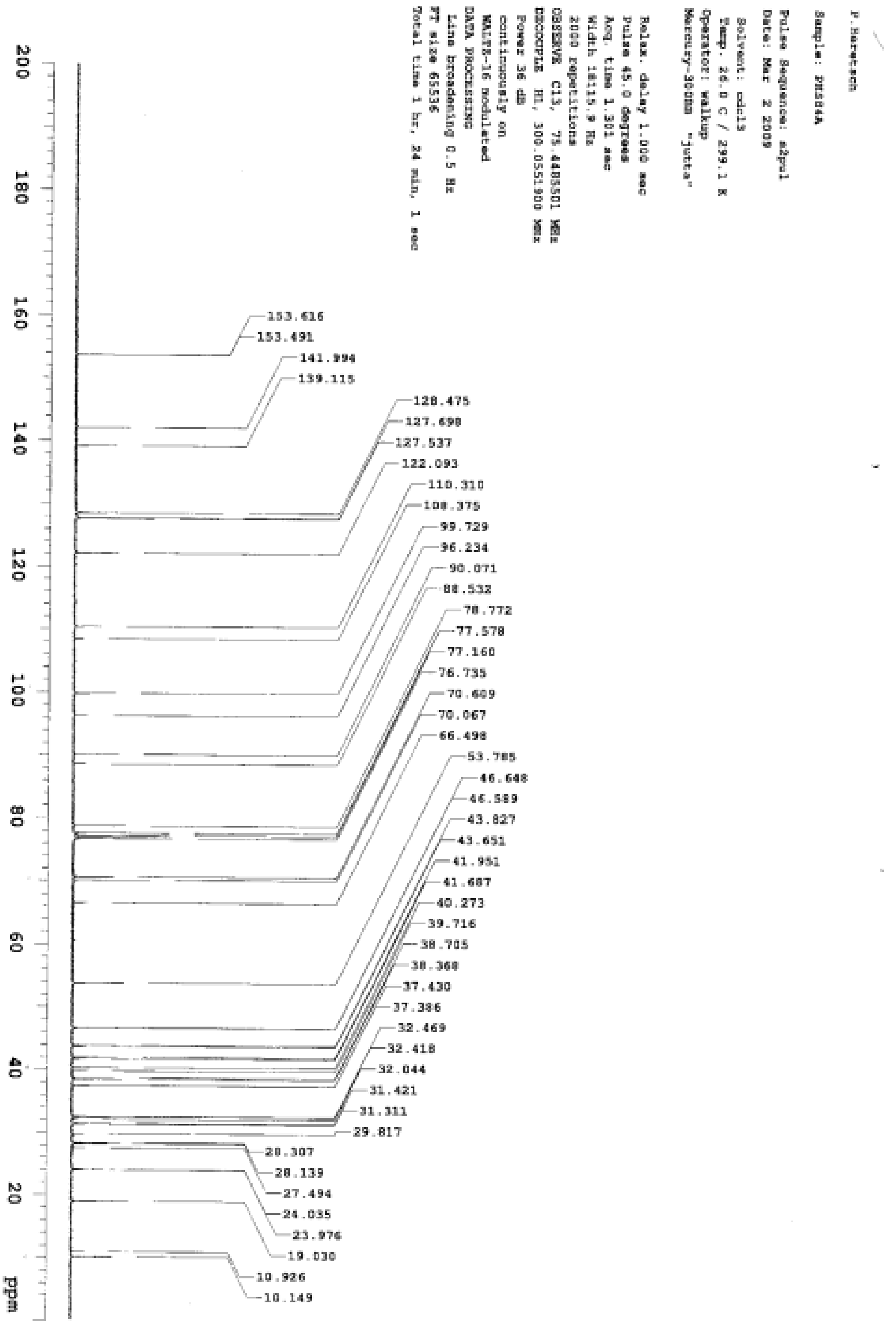

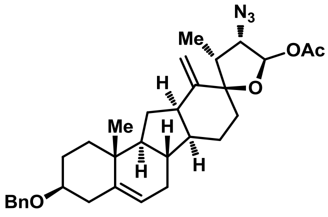

26

^{1}H NMR (400 MHz, $CDCl_3$)

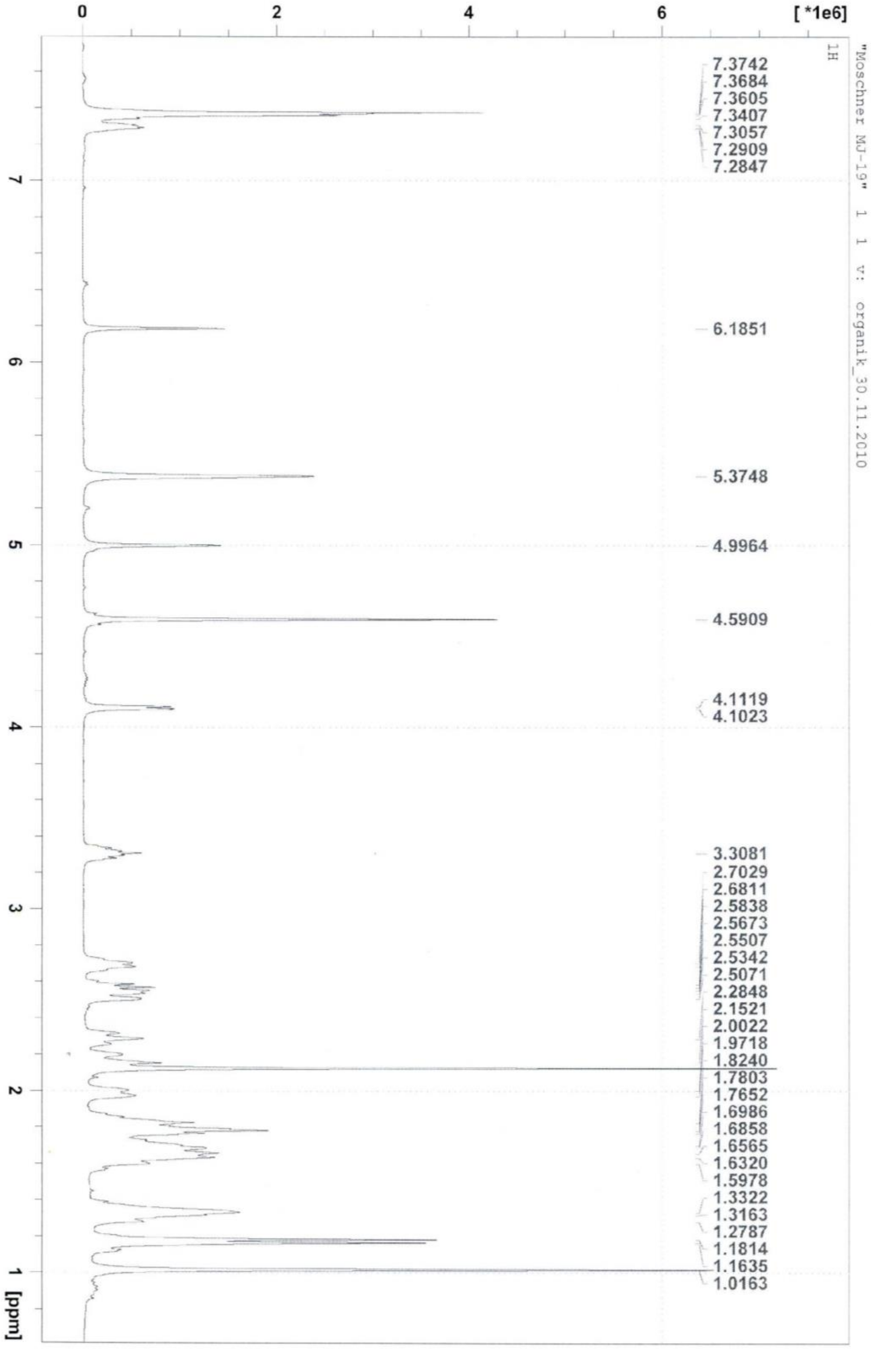

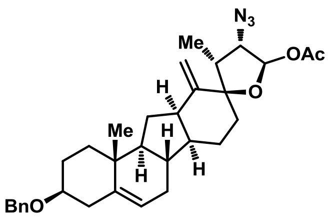

26

^{13}C NMR (100 MHz, $CDCl_3$)

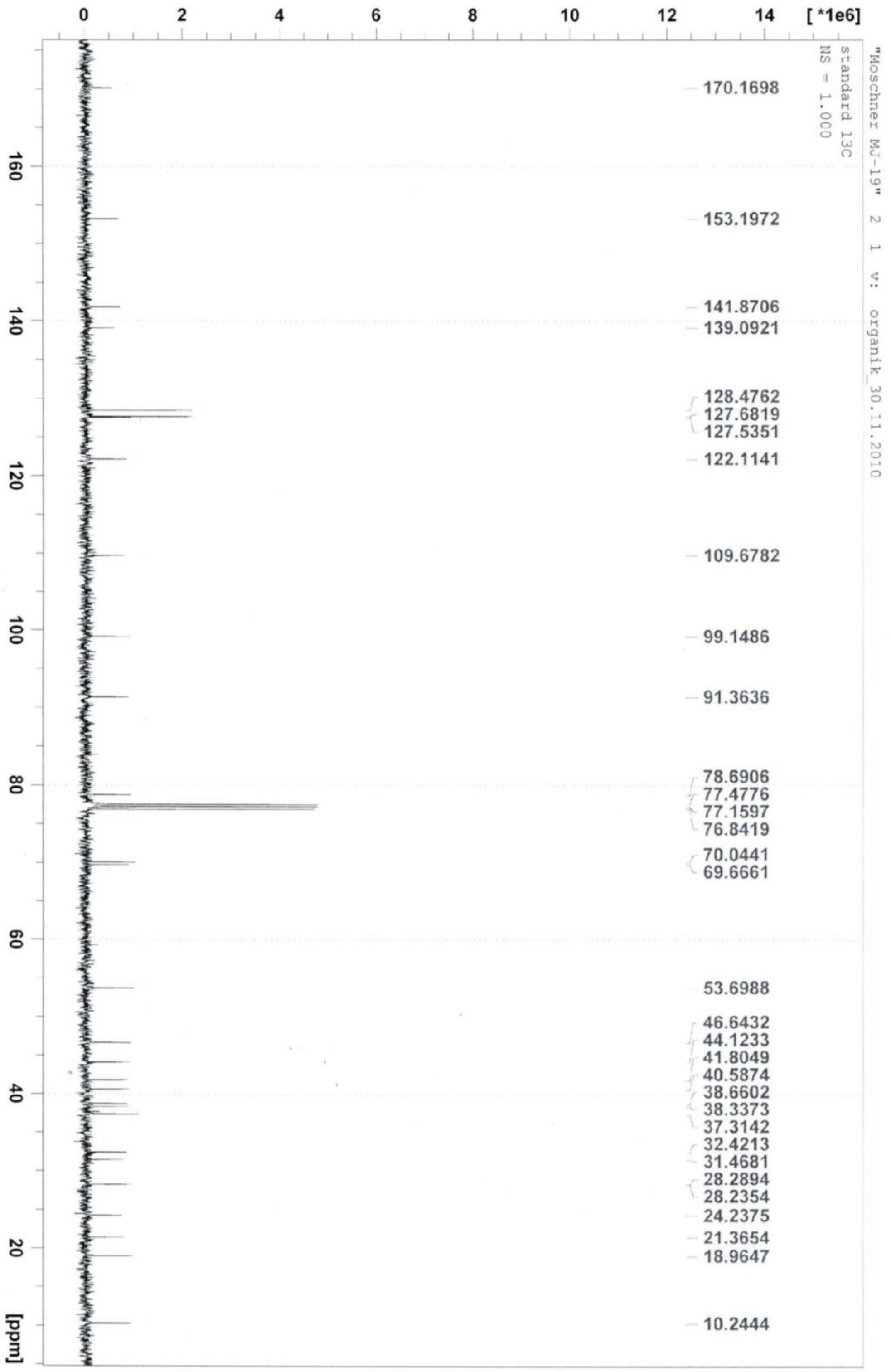

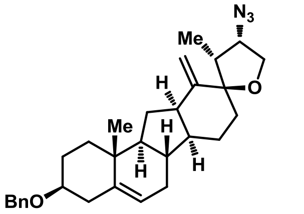

7

^{1}H NMR (400 MHz, $CDCl_3$)

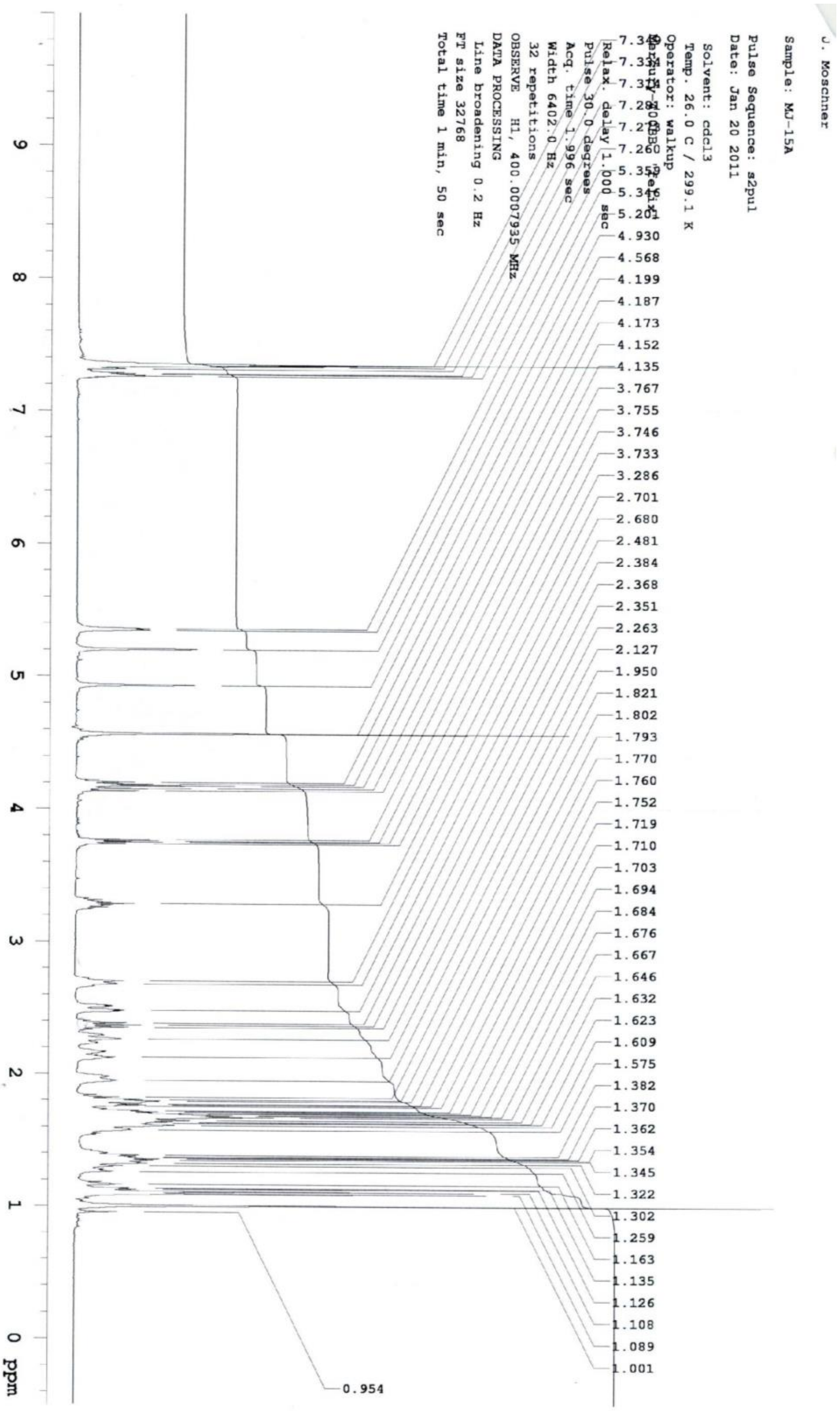

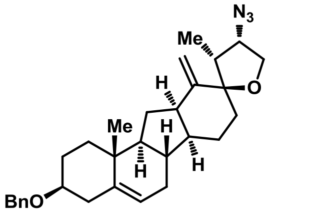

7

^{13}C NMR (100 MHz, $CDCl_3$)

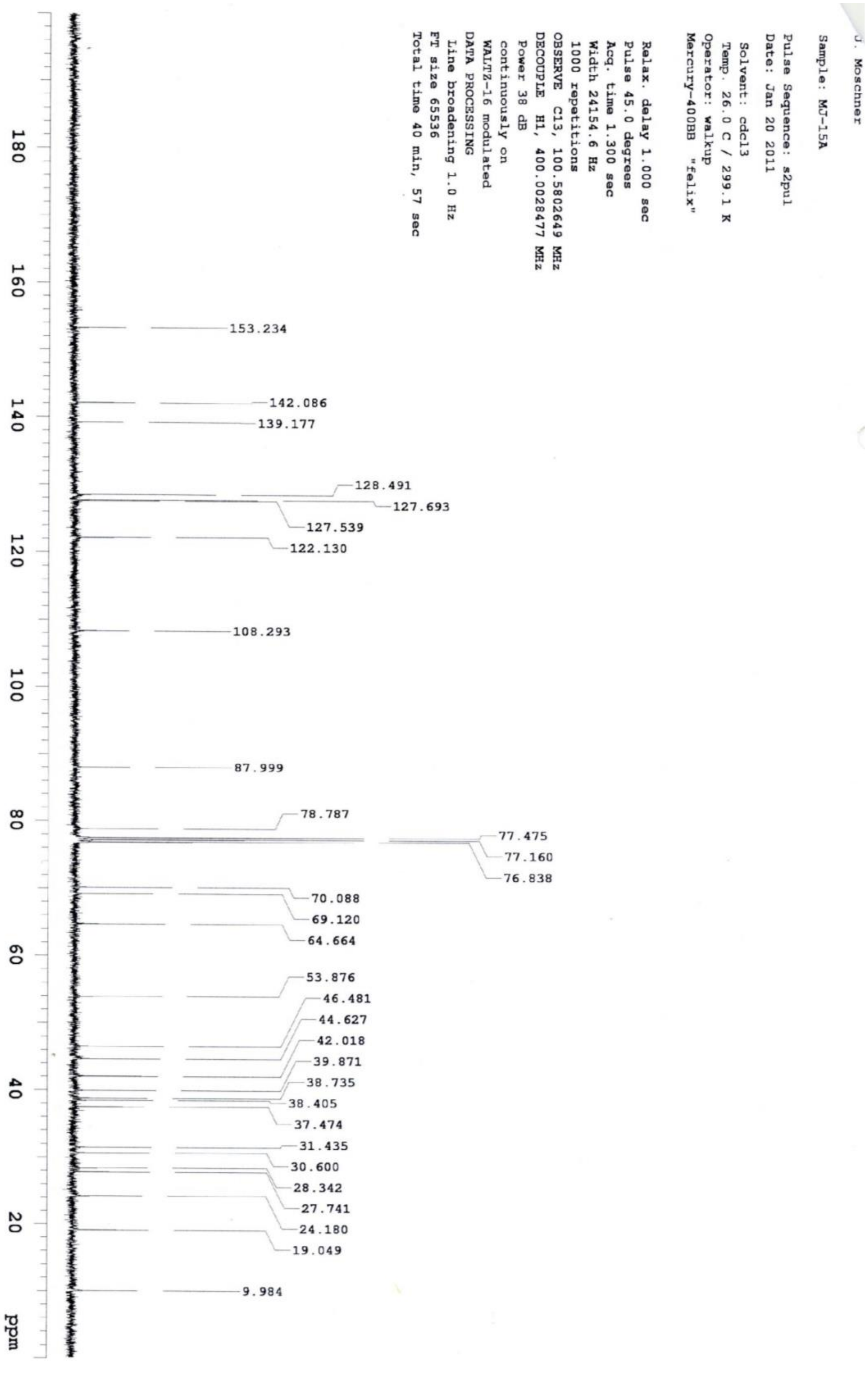

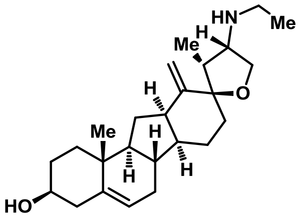

8

^{1}H NMR (300 MHz, $CDCl_3$)

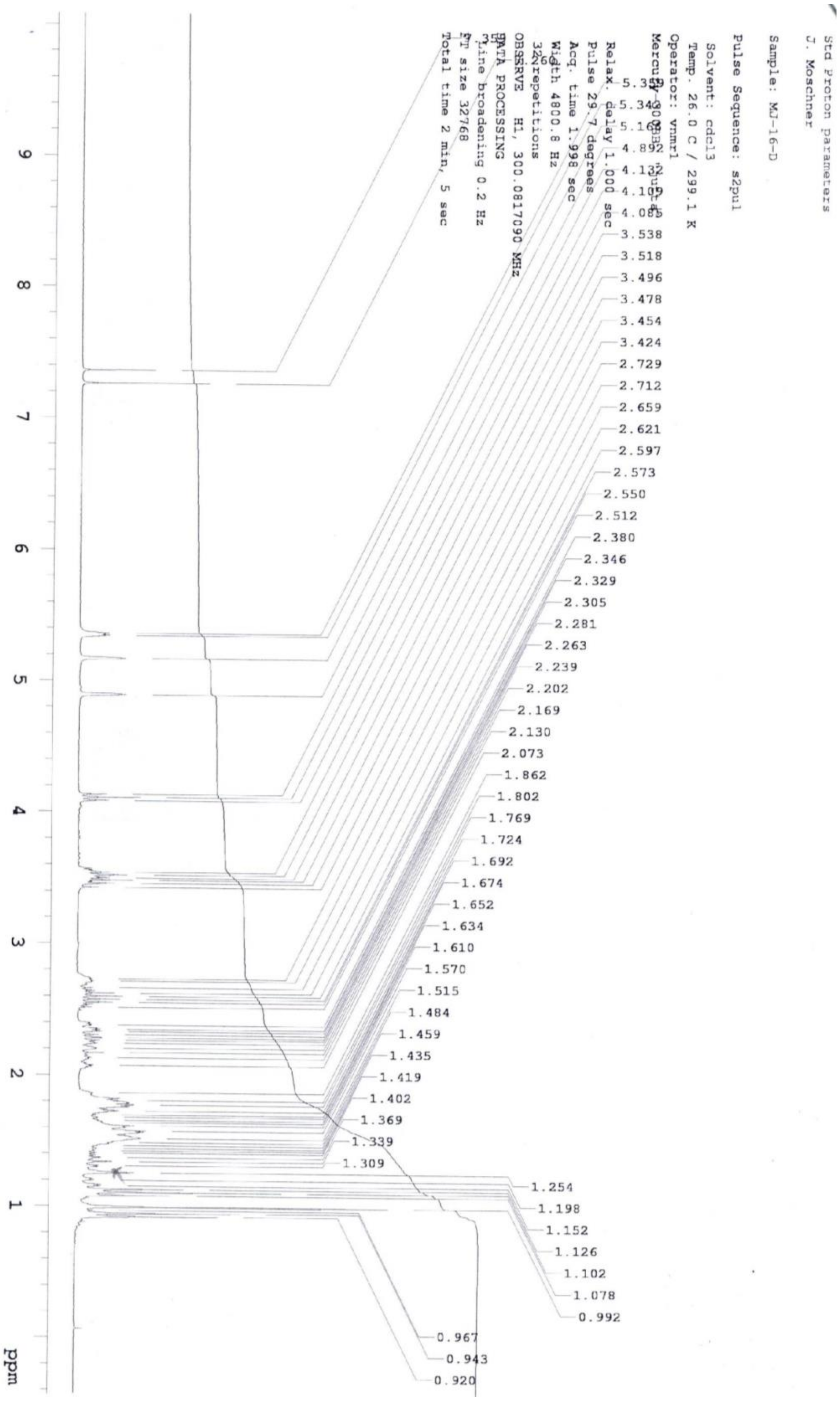

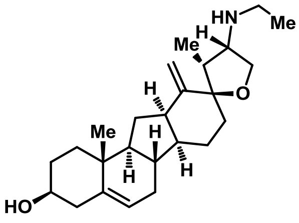

8

APT (75 MHz, $CDCl_3$)

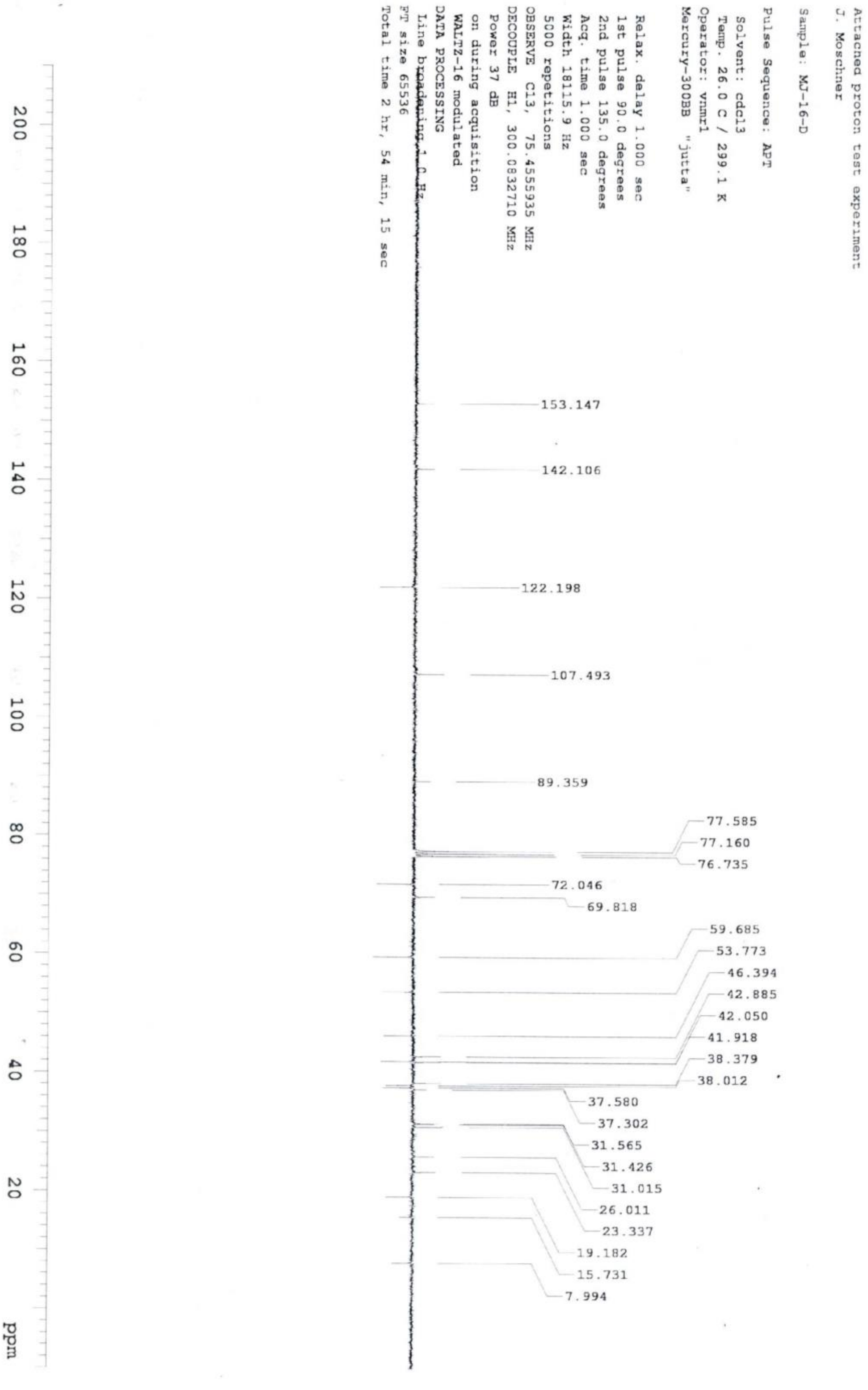

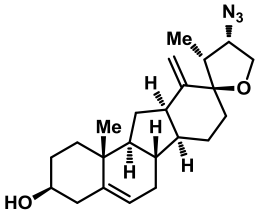

27

^{1}H NMR (400 MHz, $CDCl_3$)

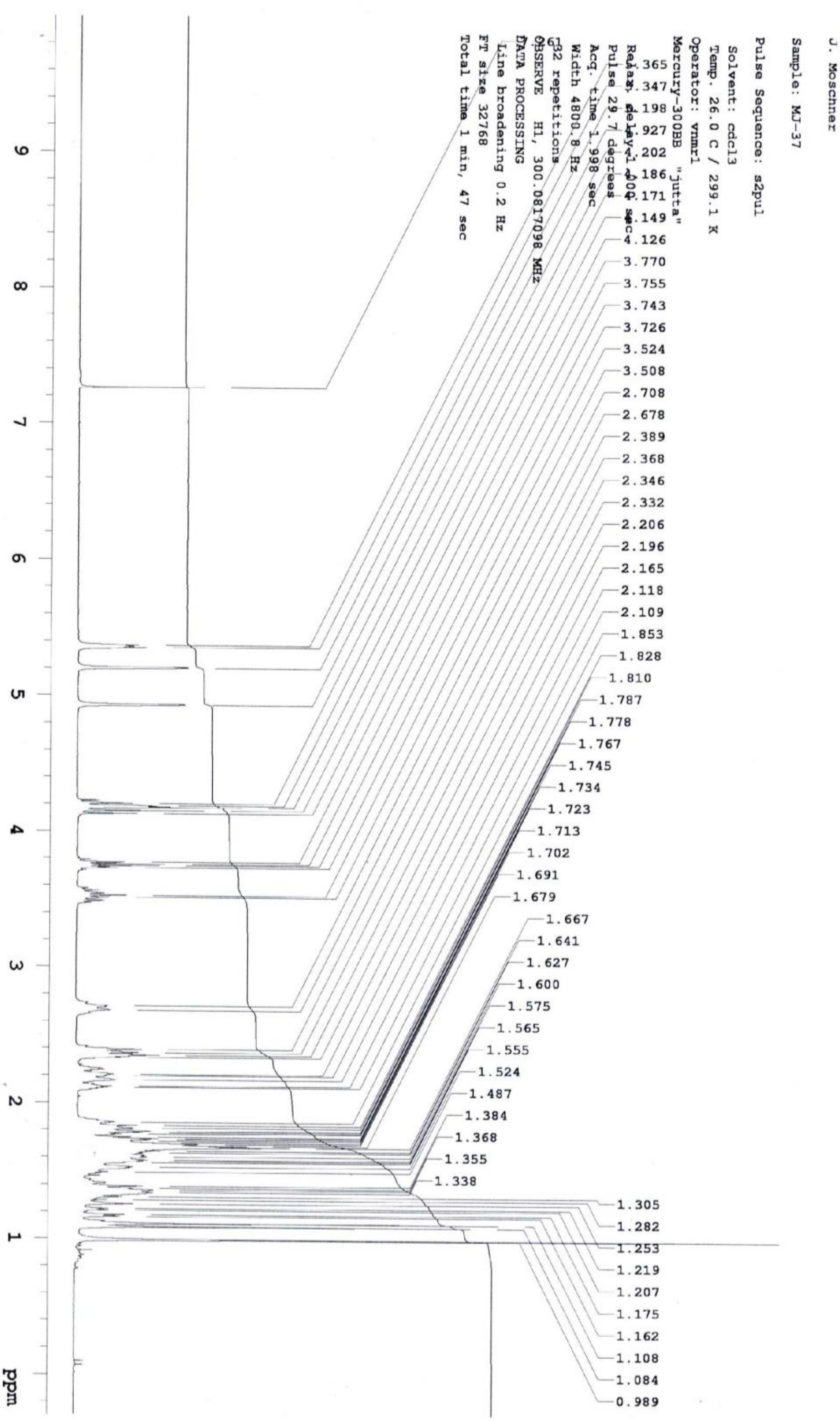

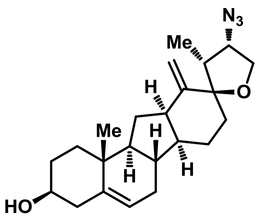

27

^{13}C NMR (75 MHz, $CDCl_3$)

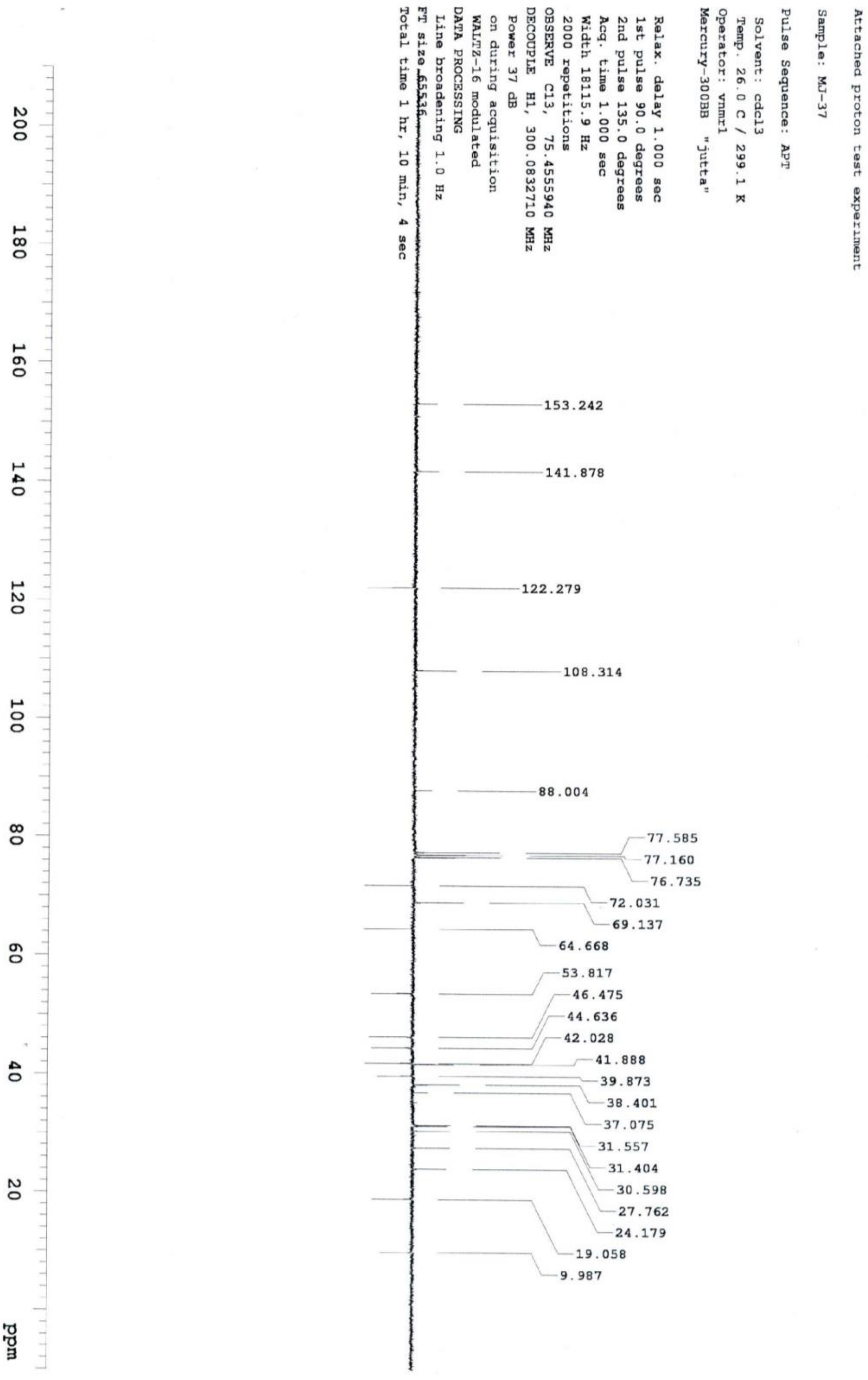

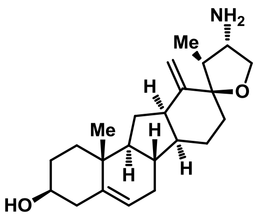

9

^{1}H NMR (300 MHz, CD_3OD)

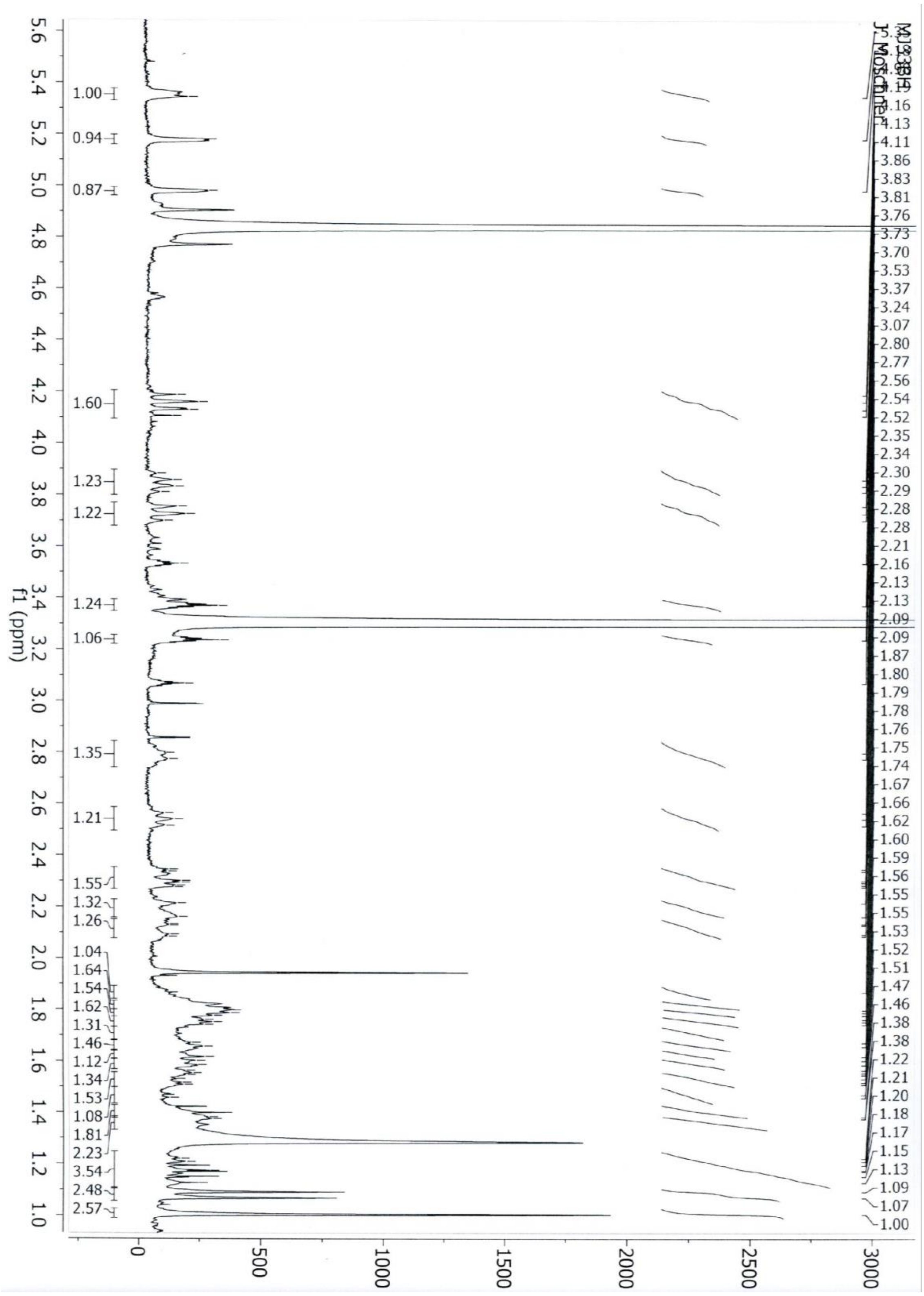

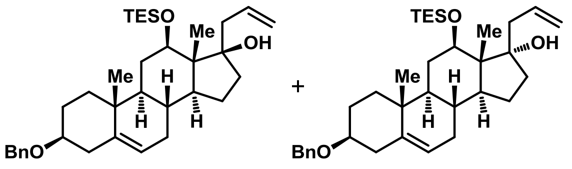

11 **epi-11**

^{1}H NMR (400 MHz, $CDCl_3$)

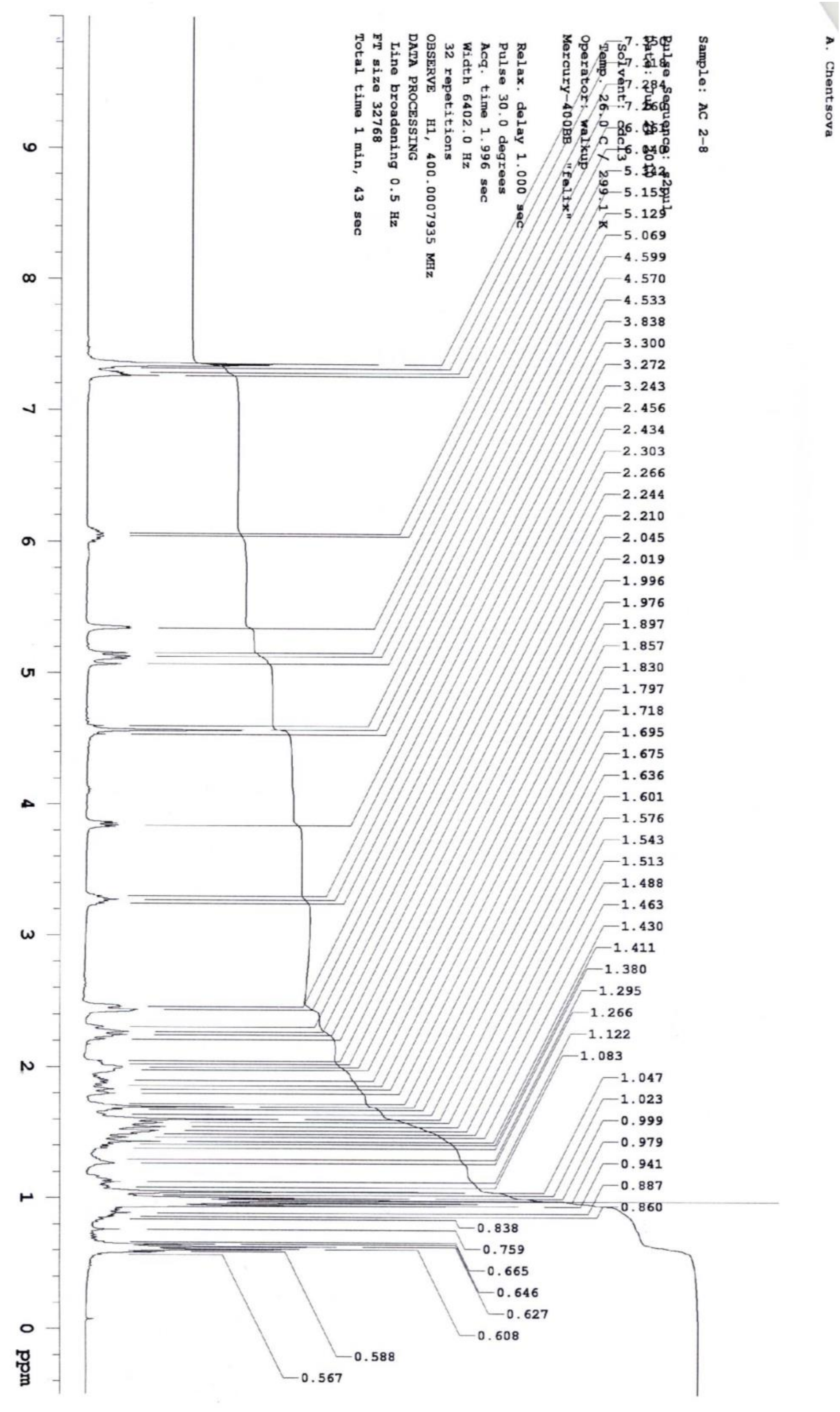

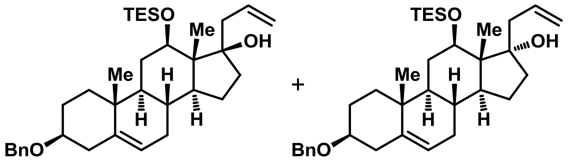

11 **epi-11**

^{13}C NMR (100 MHz, $CDCl_3$)

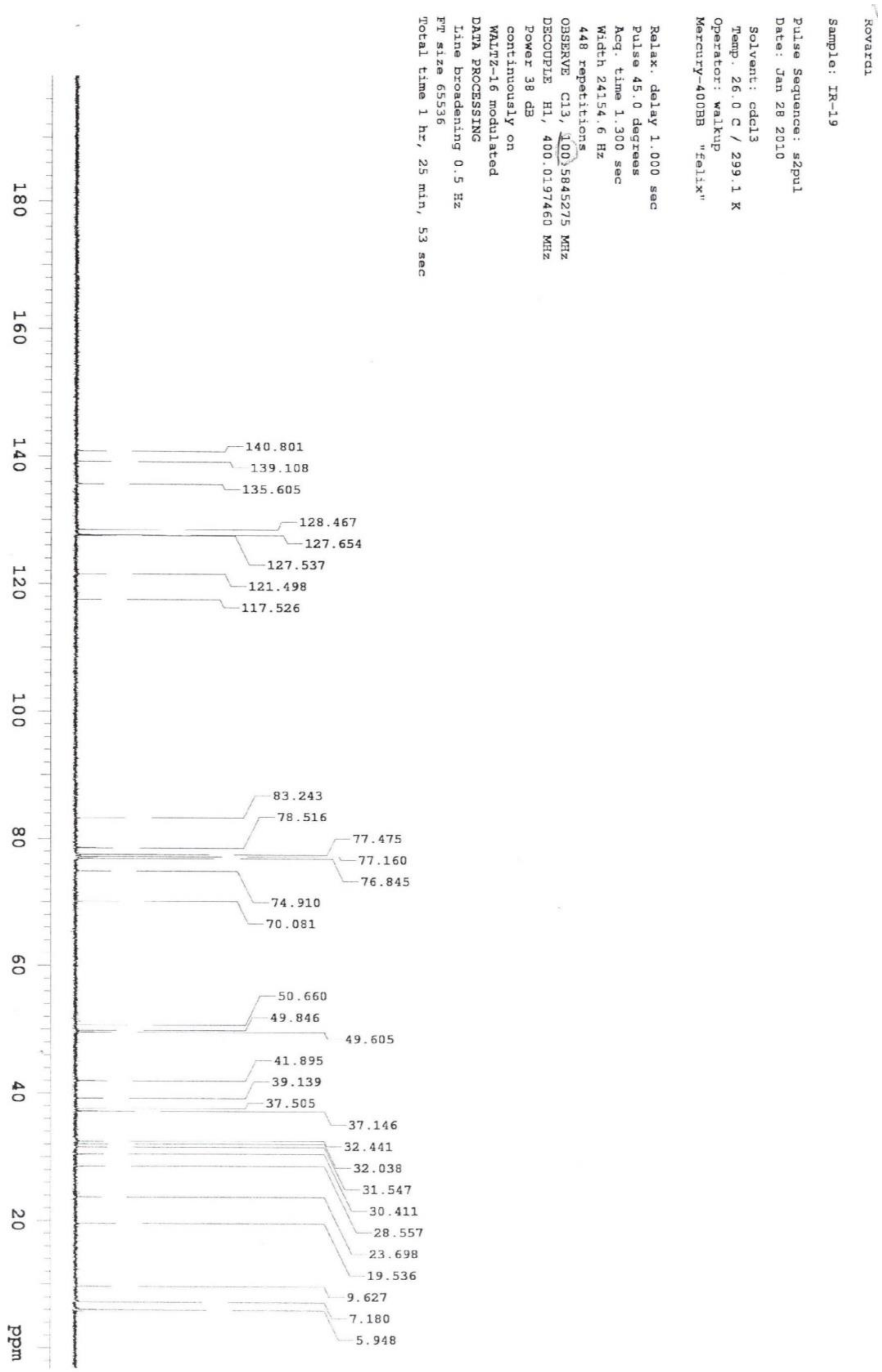

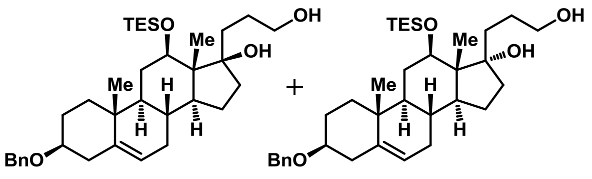

28 **epi-28**

^{1}H NMR (300 MHz, CDCl$_3$)

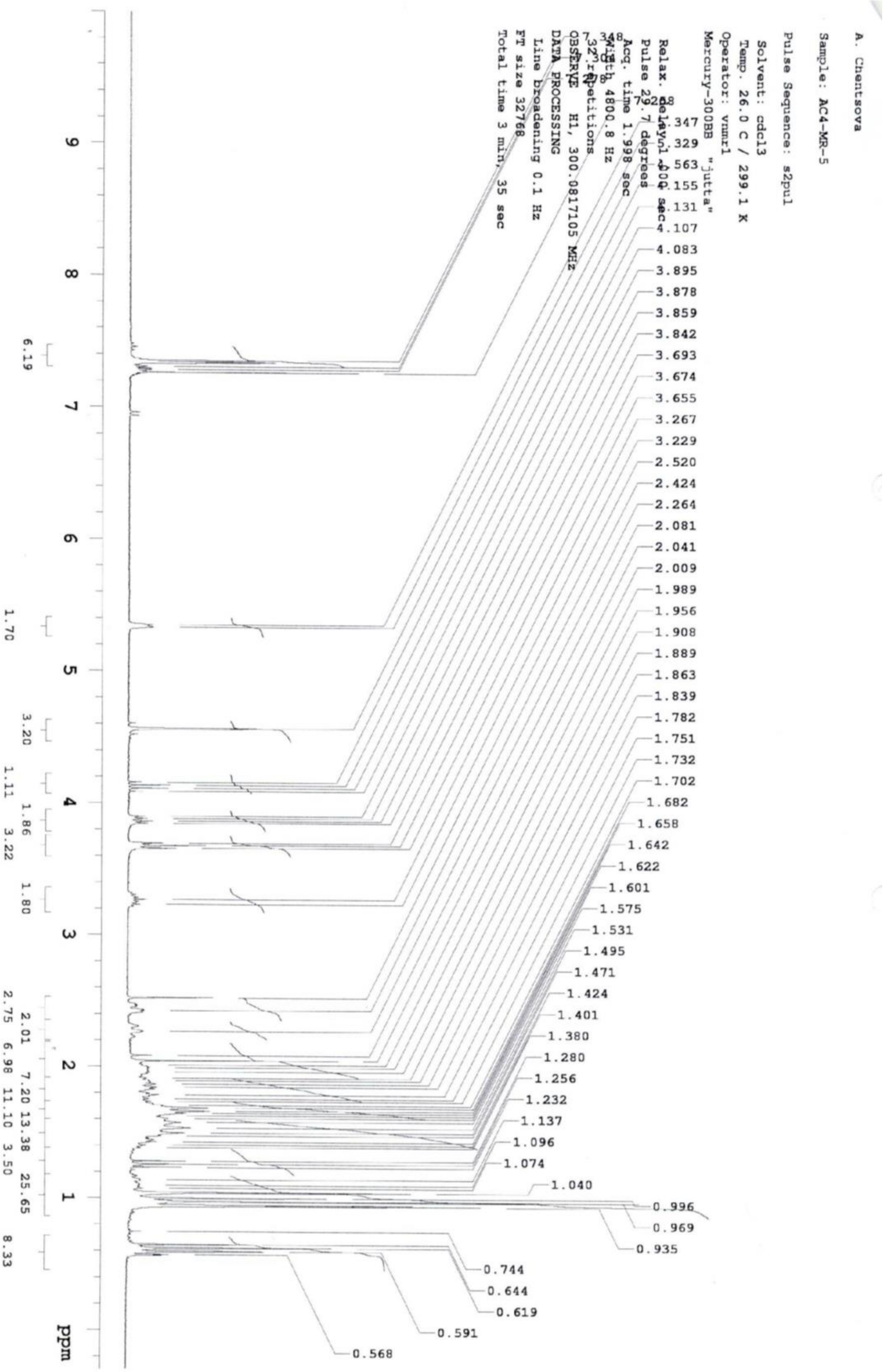

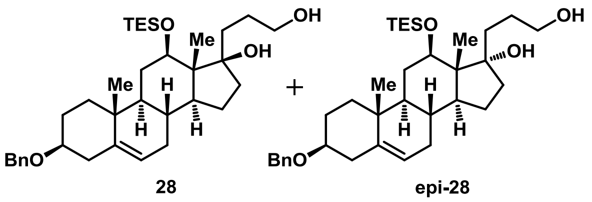

28 + **epi-28**

^{13}C NMR (75 MHz, $CDCl_3$)

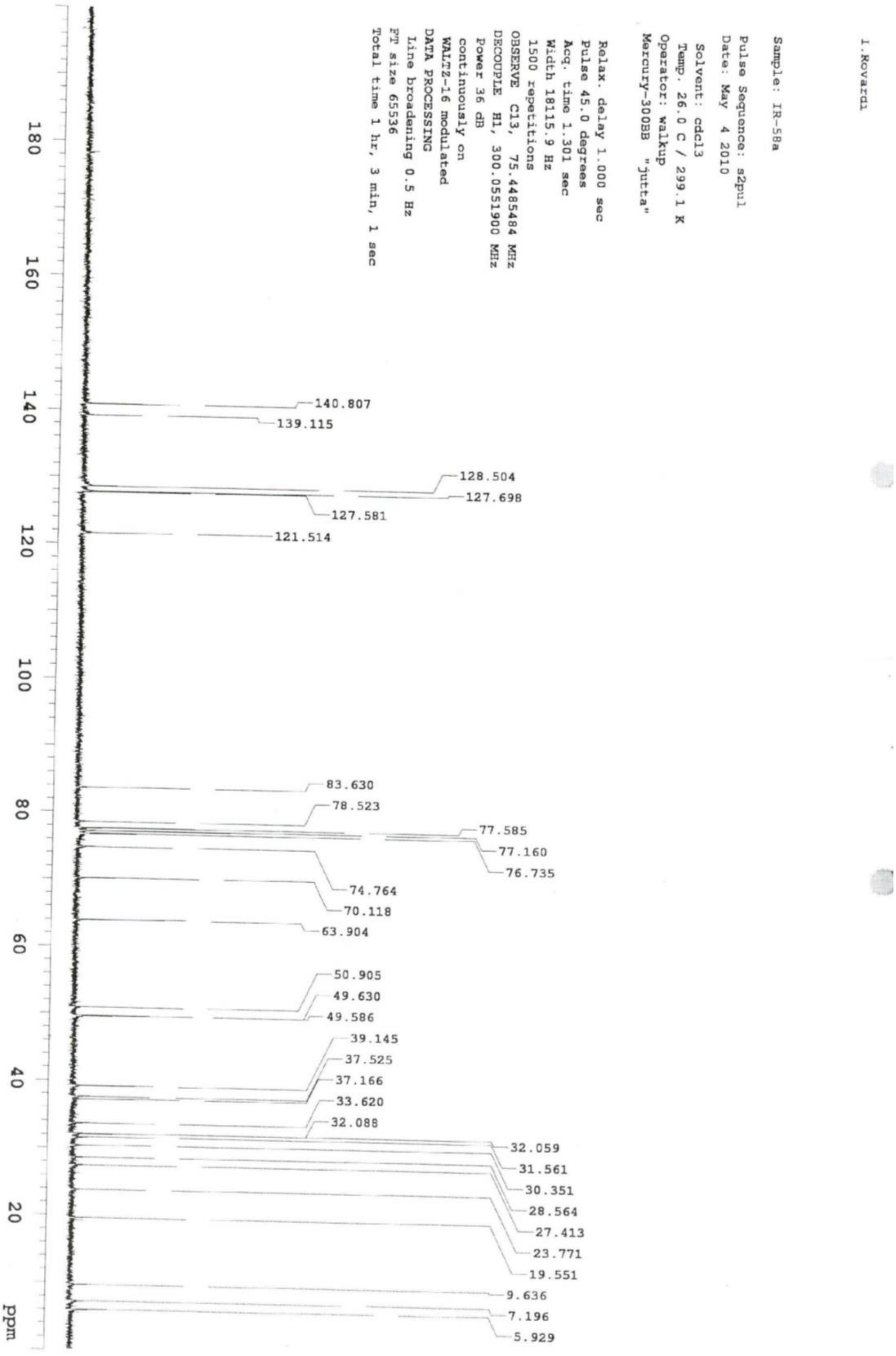

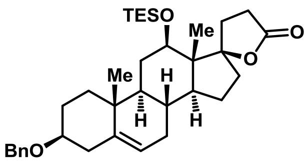

12

^{1}H NMR (400 MHz, $CDCl_3$)

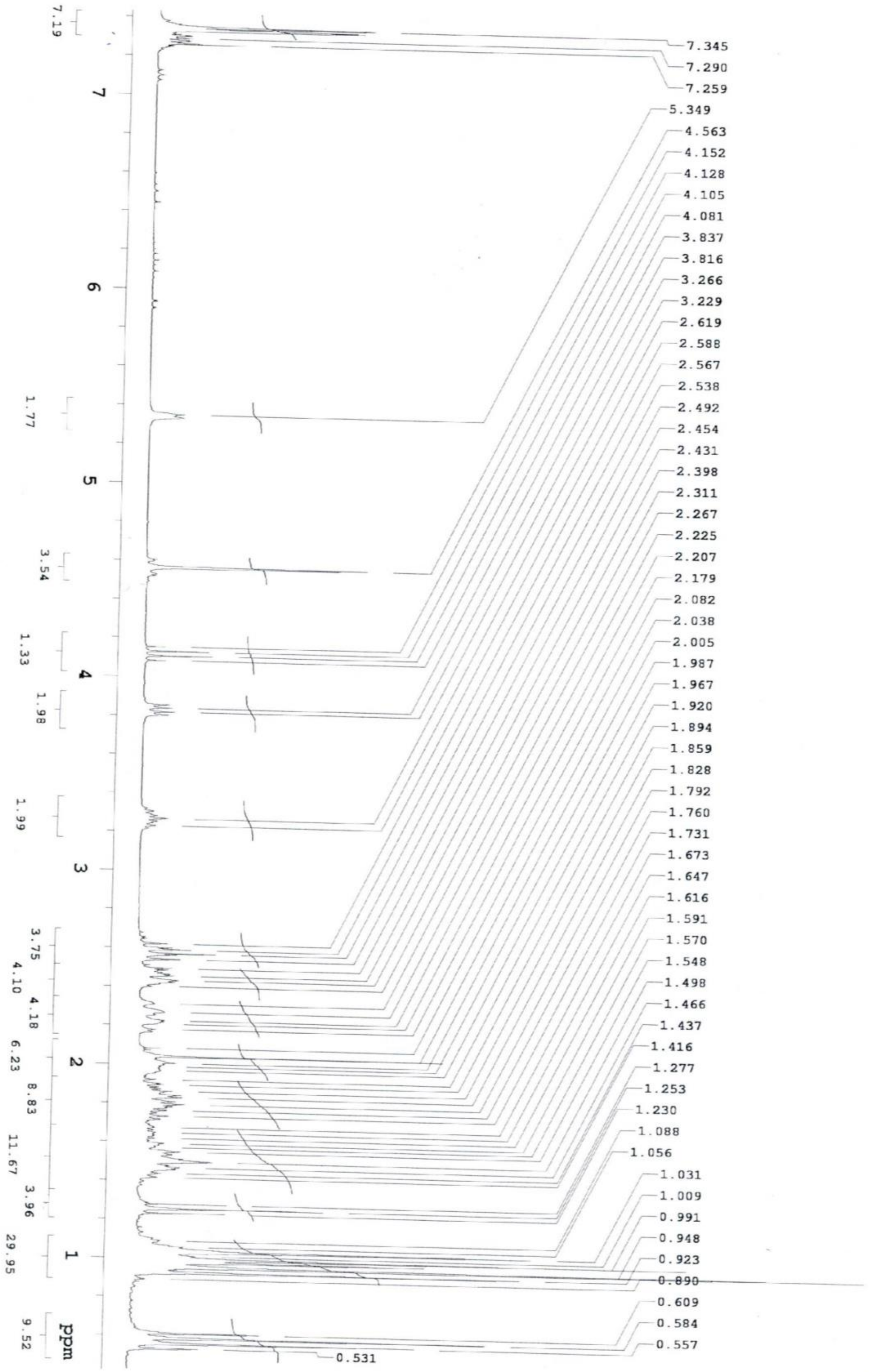

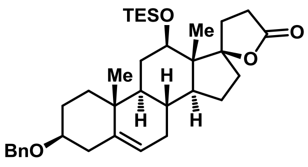

12

^{13}C NMR (100 MHz, $CDCl_3$)

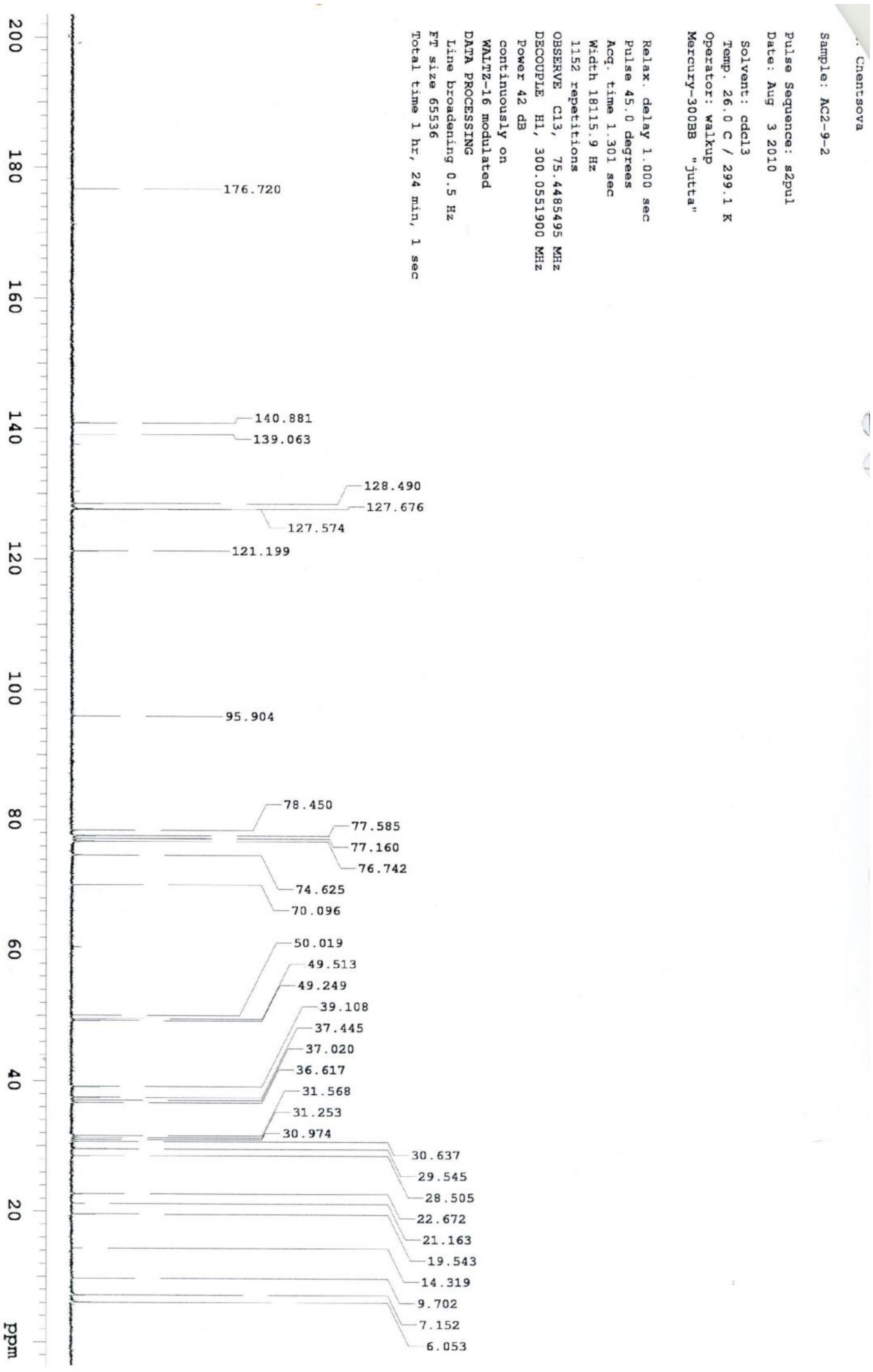

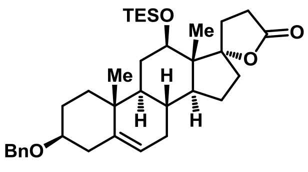

epi-12

^{1}H NMR (400 MHz, $CDCl_3$)

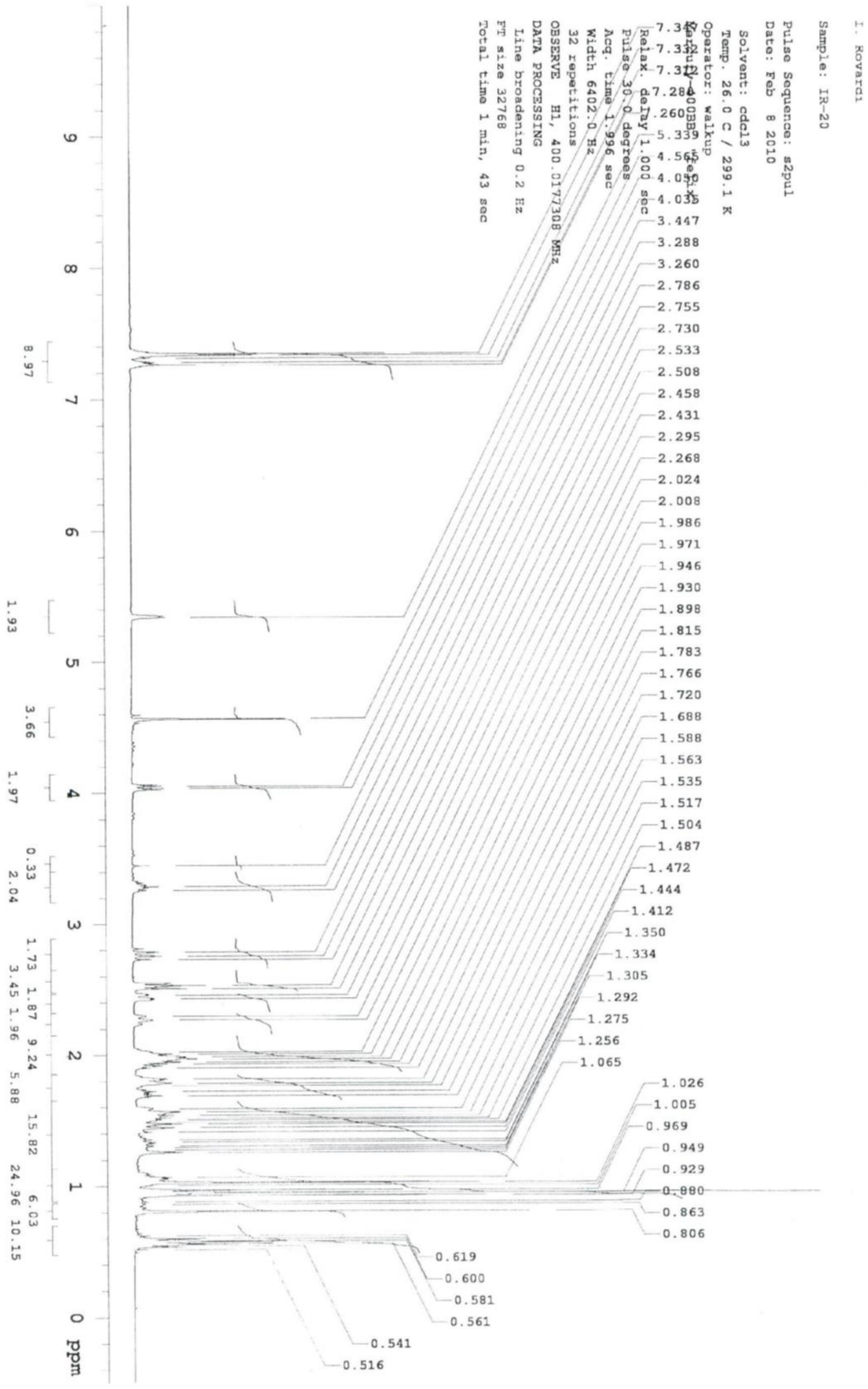

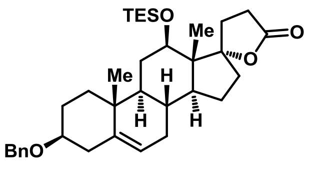

epi-12

^{13}C NMR (100 MHz, $CDCl_3$)

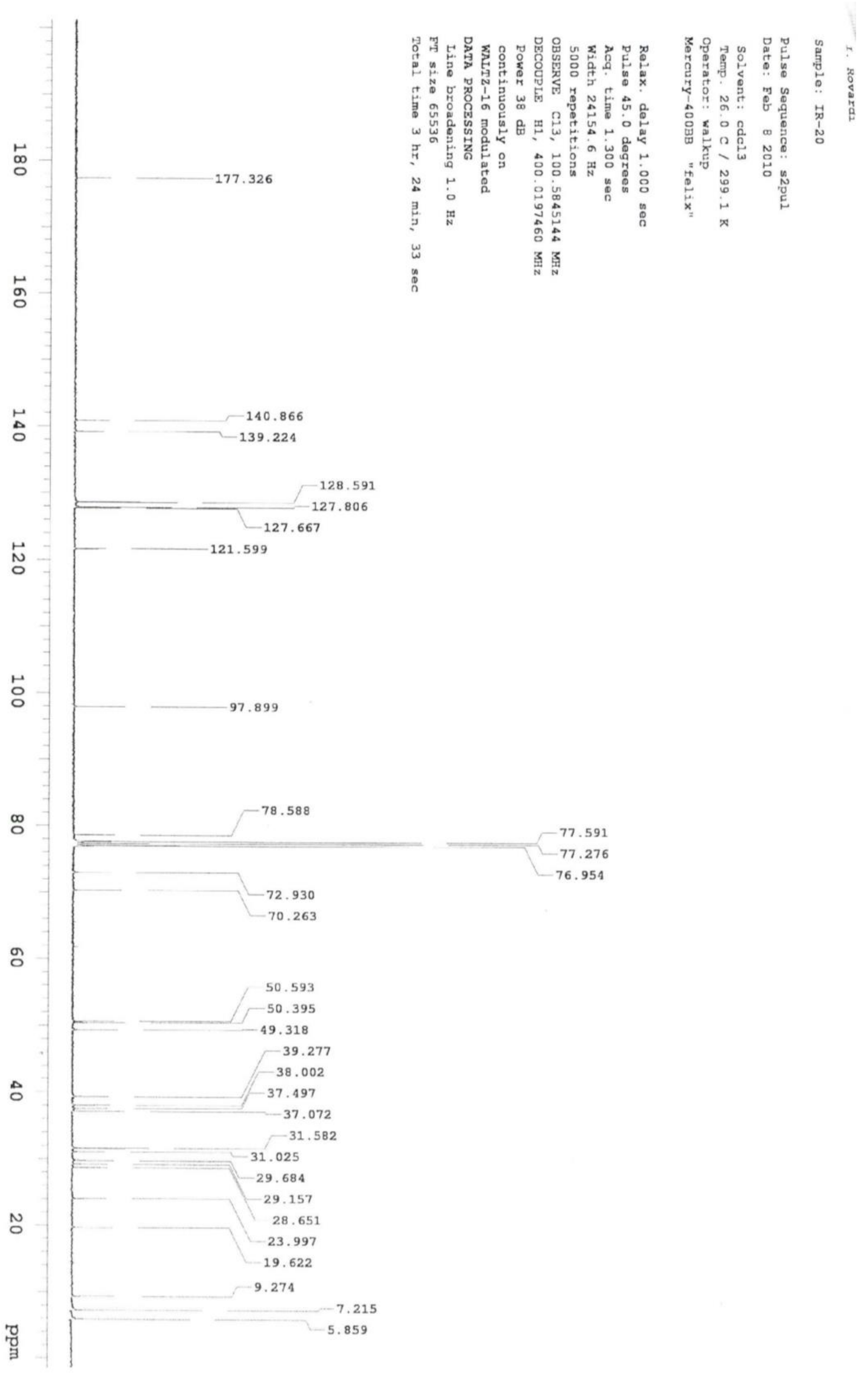

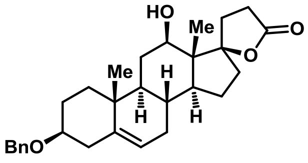

29

^{1}H NMR (400 MHz, $CDCl_3$)

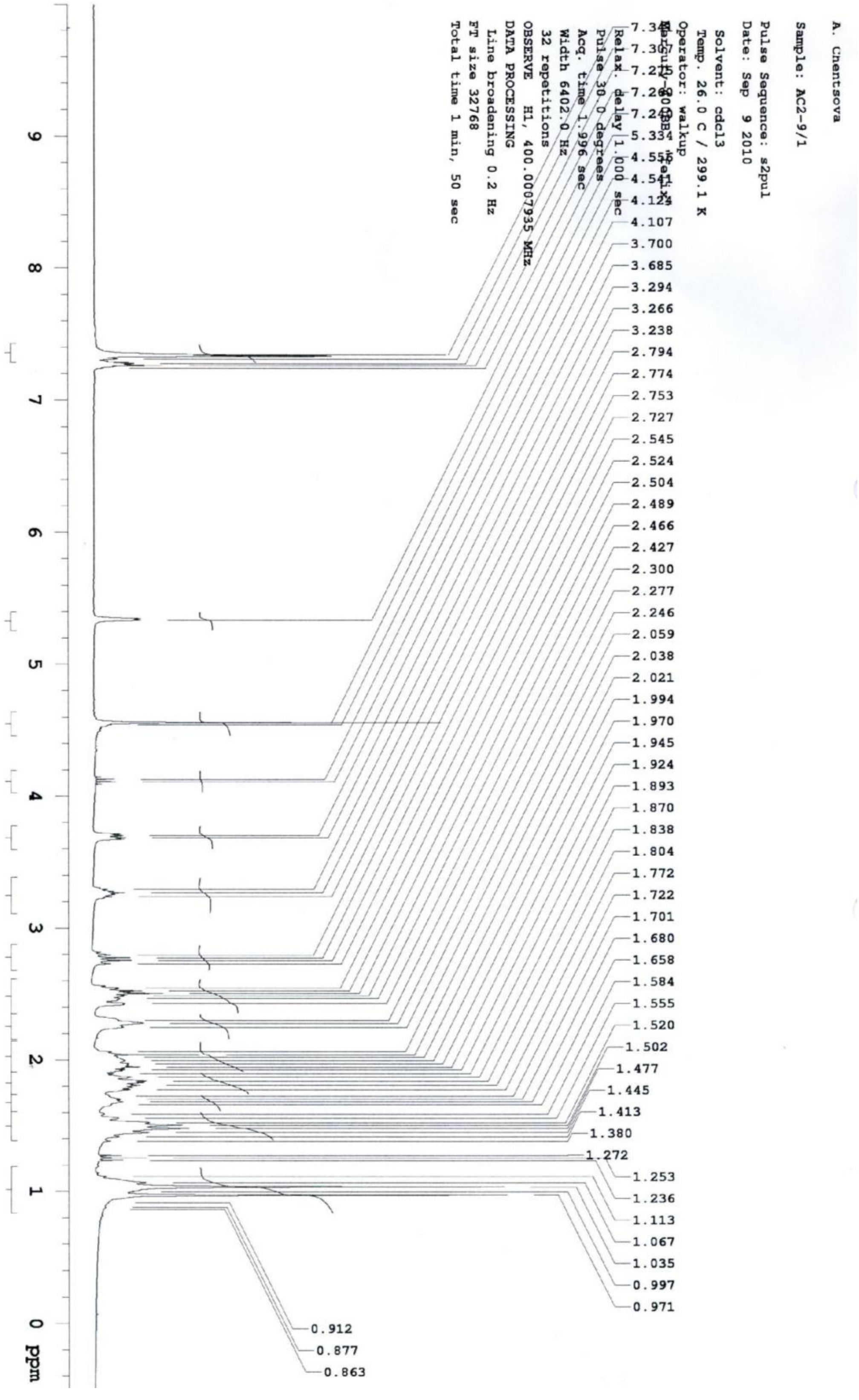

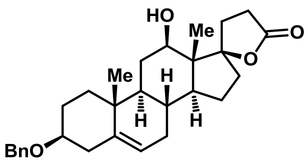

29

^{13}C NMR (100 MHz, $CDCl_3$)

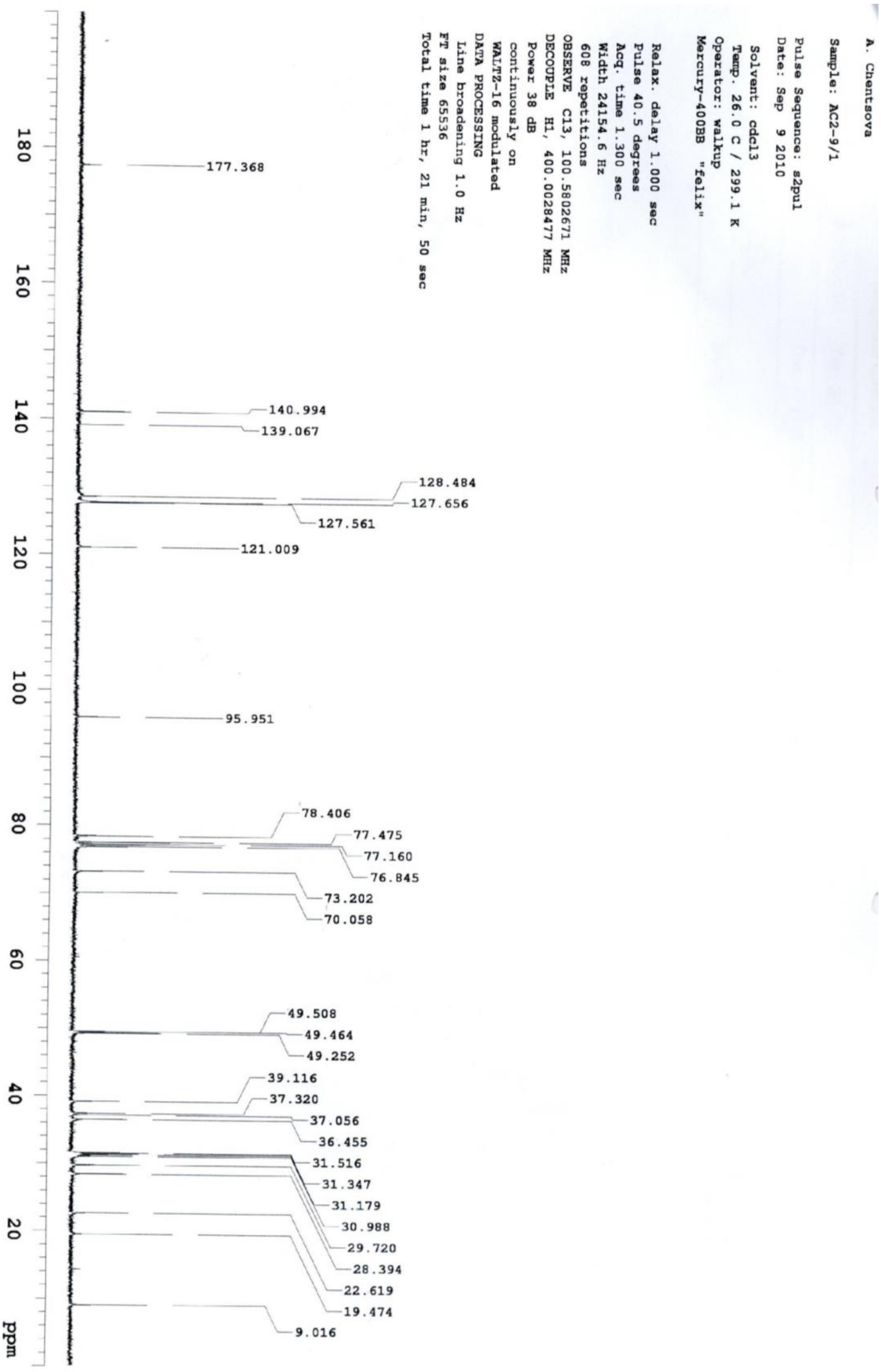

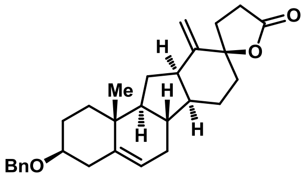

13

^{1}H NMR (300 MHz, $CDCl_3$)

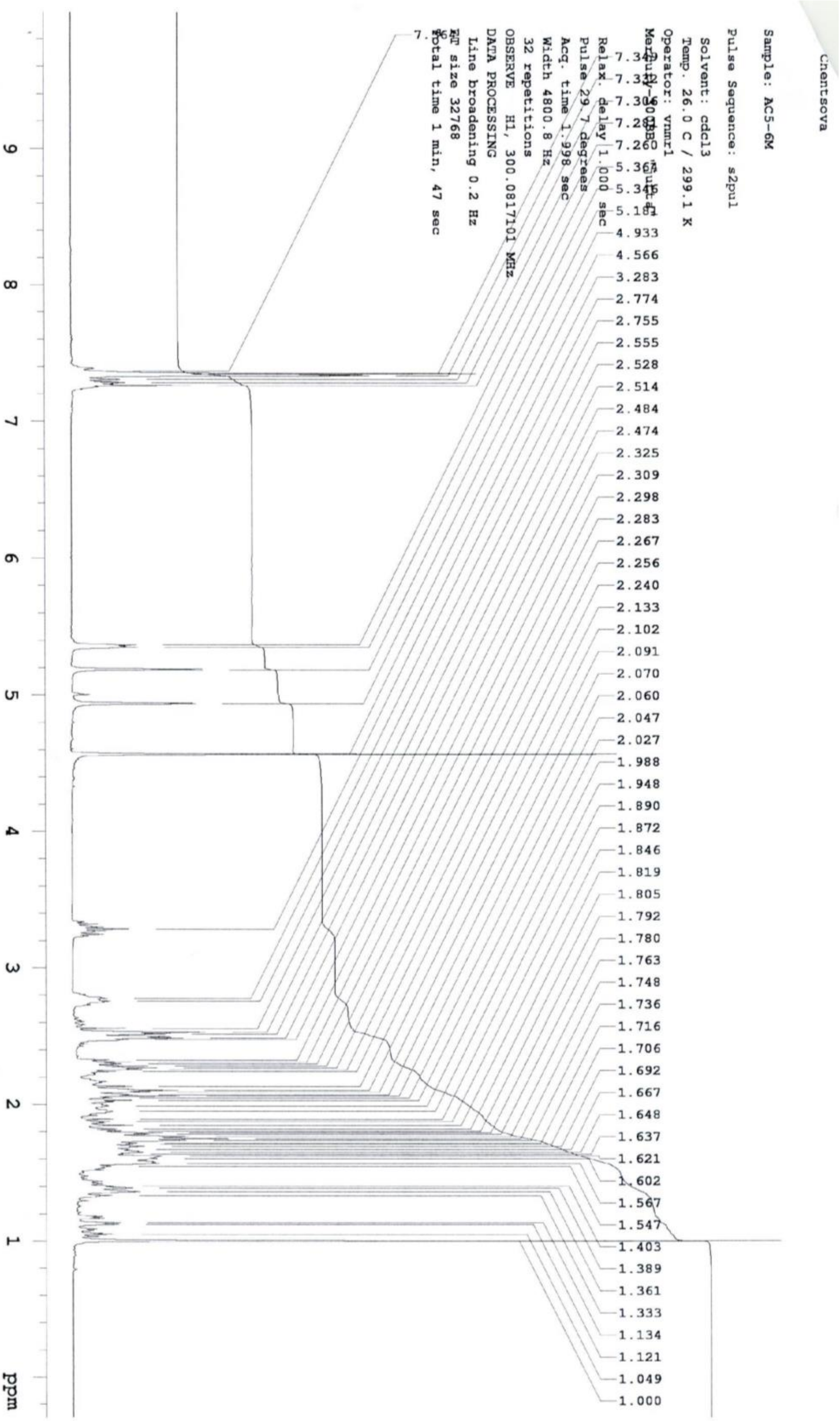

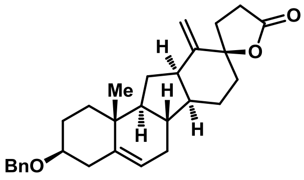

13

APT (75 MHz, $CDCl_3$)

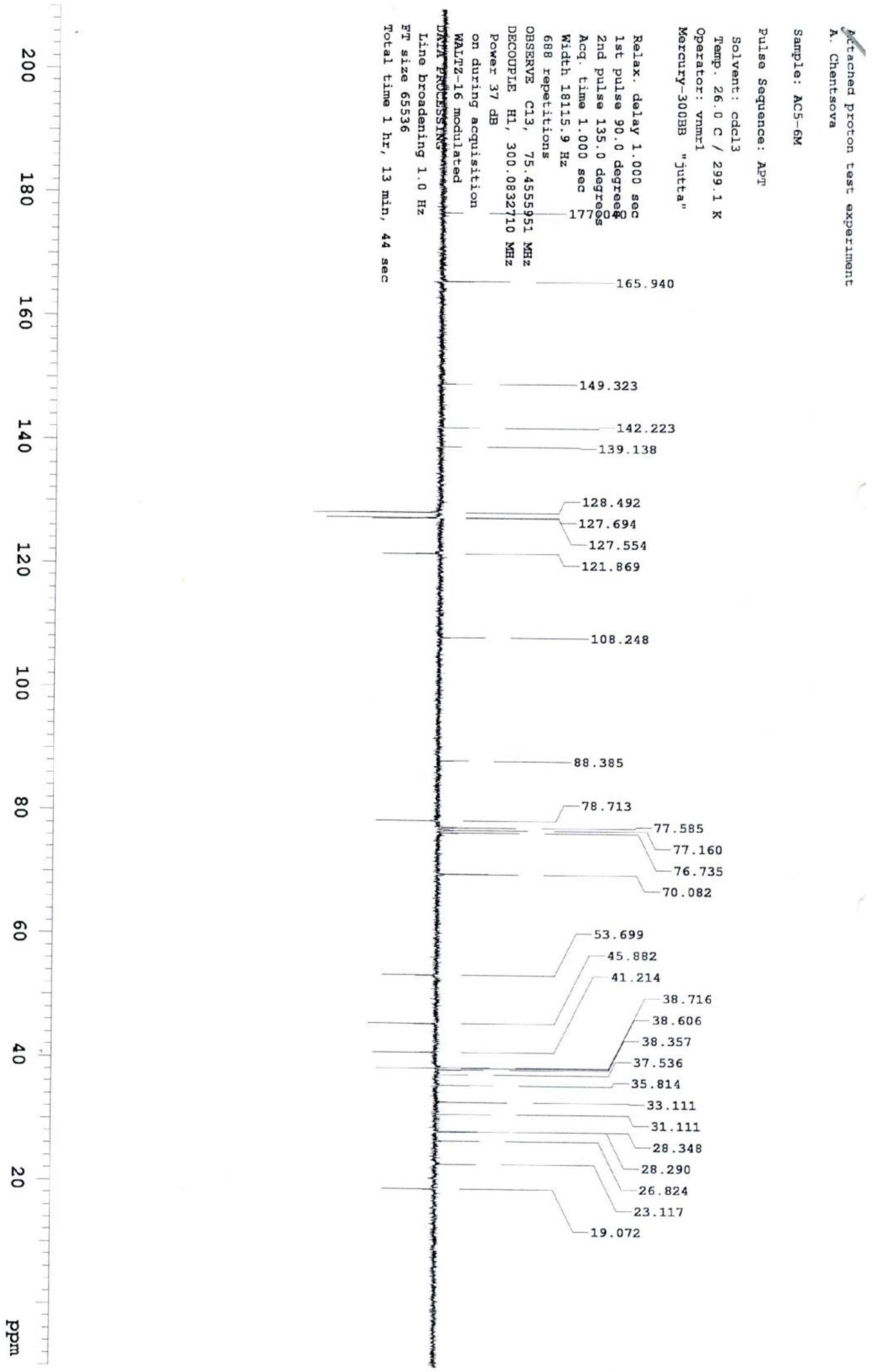

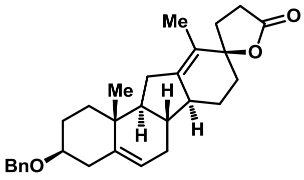

30

^{1}H NMR (300 MHz, $CDCl_3$)

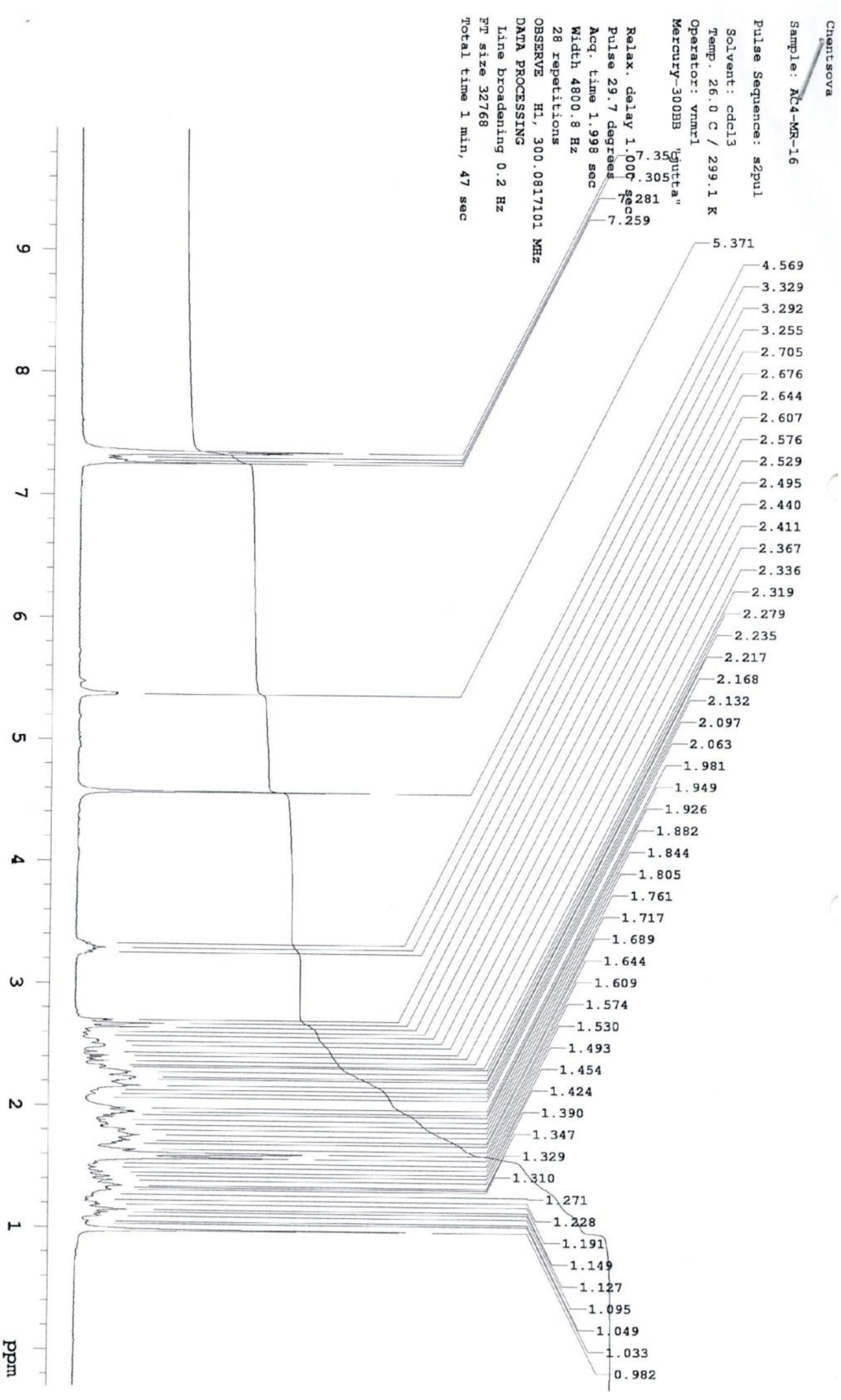

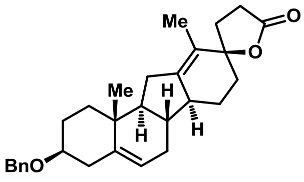

30

^{13}C NMR (75 MHz, $CDCl_3$)

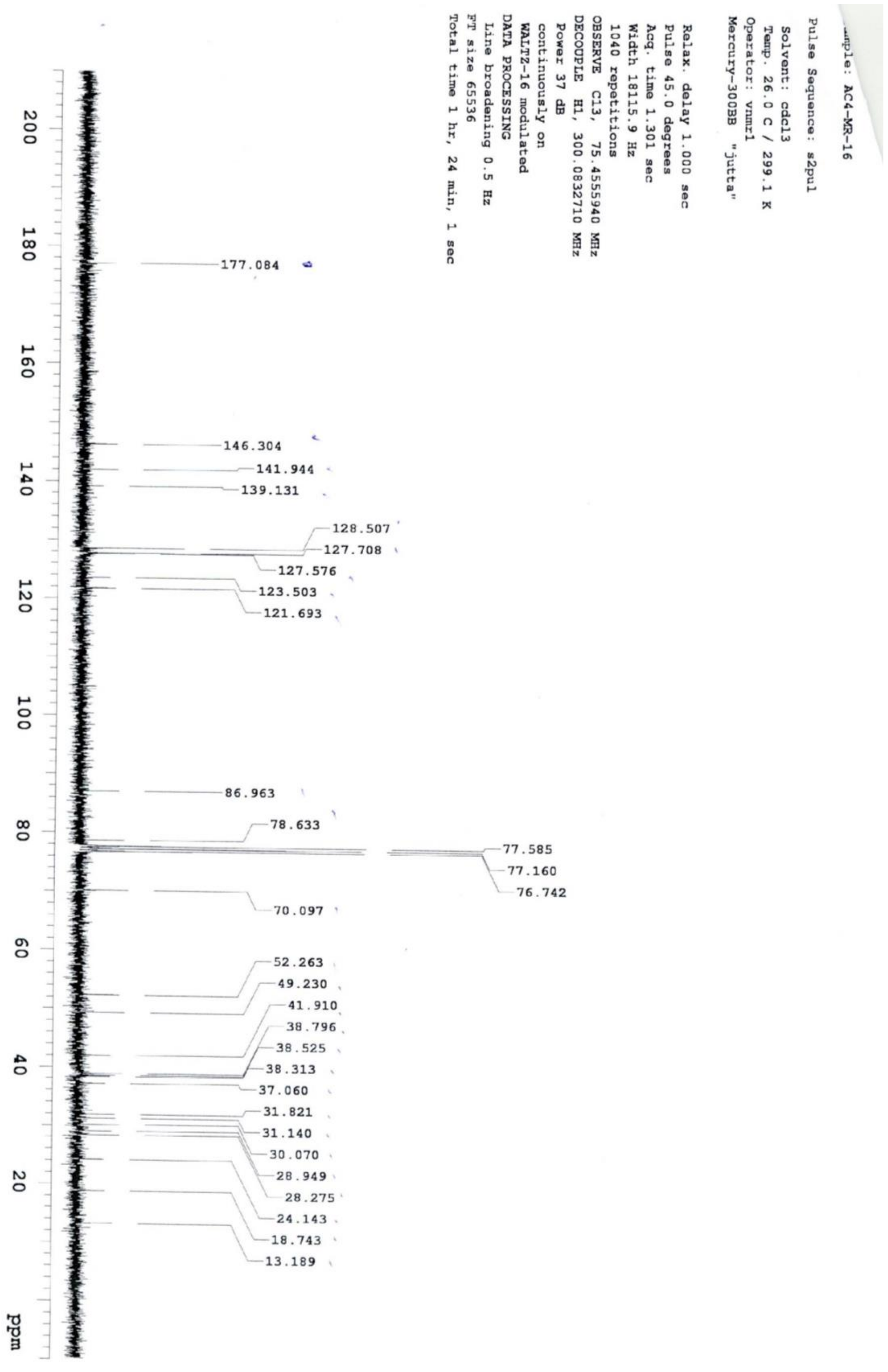

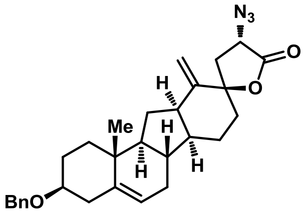

14

^{1}H NMR (400 MHz, $CDCl_3$)

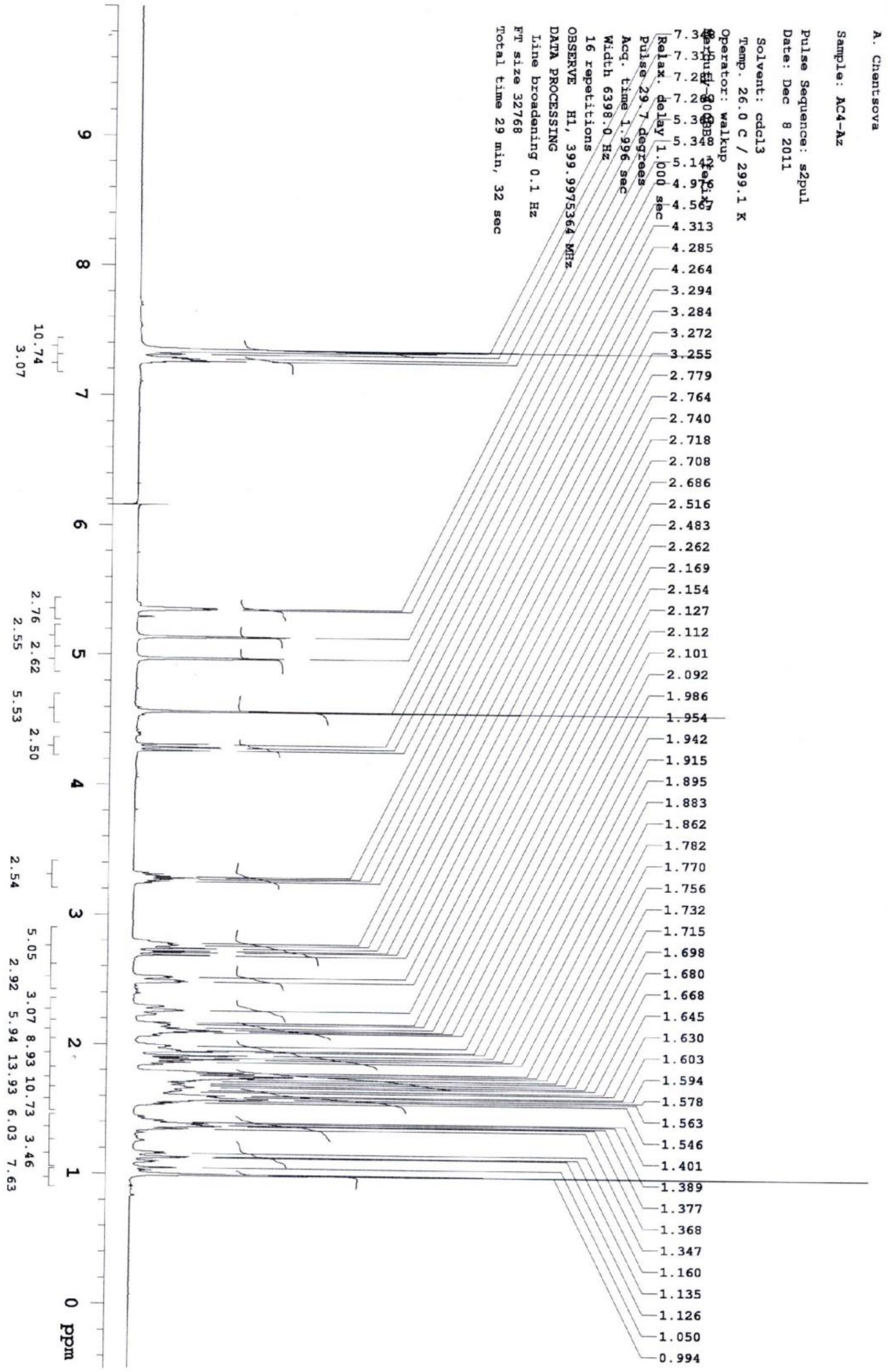

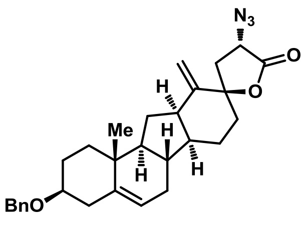

14

^{13}C NMR (100 MHz, $CDCl_3$)

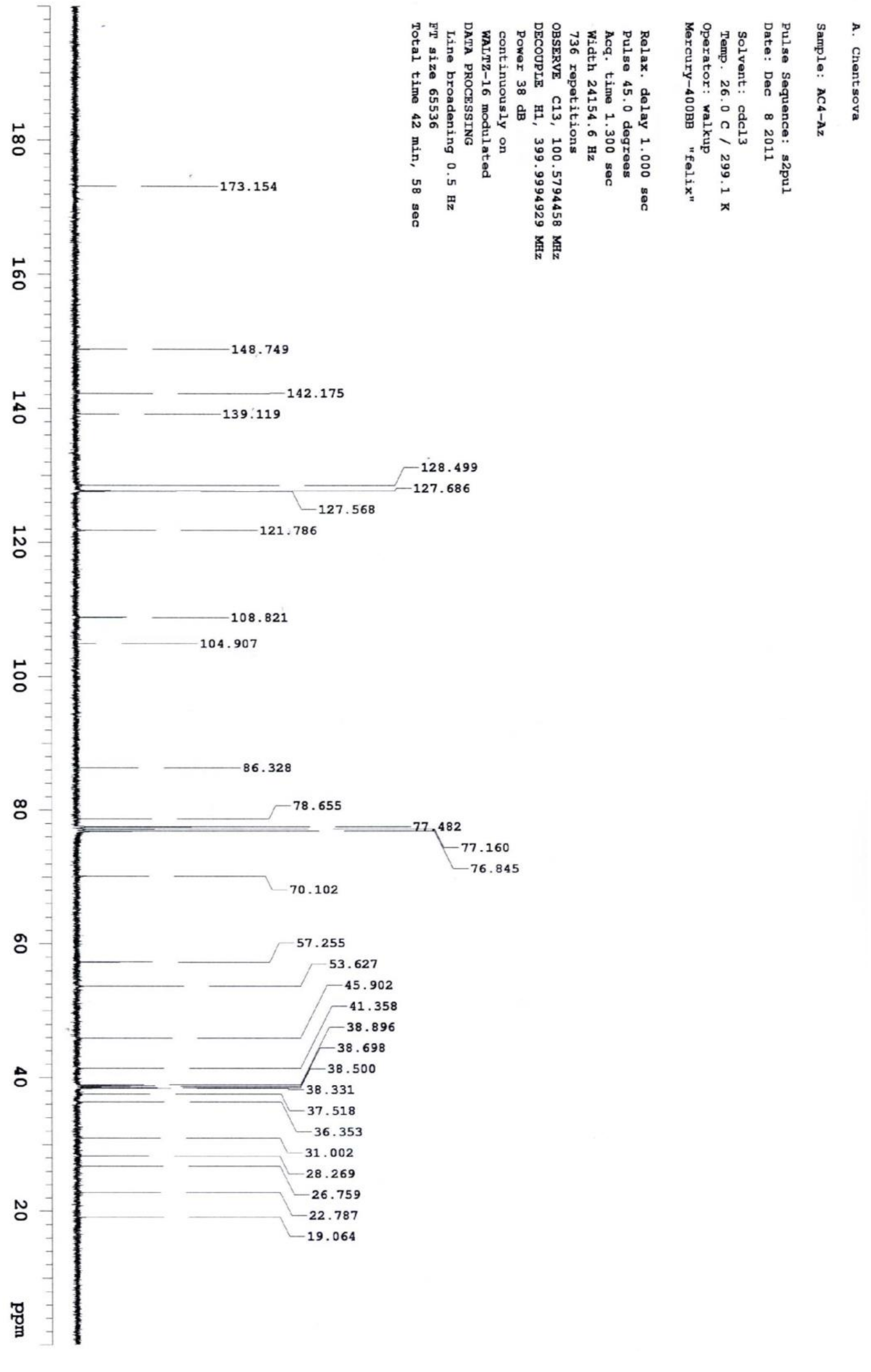

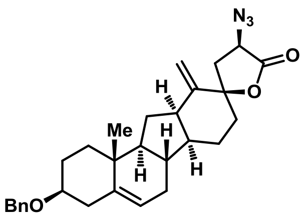

epi-14

^{1}H NMR (400 MHz, $CDCl_3$)

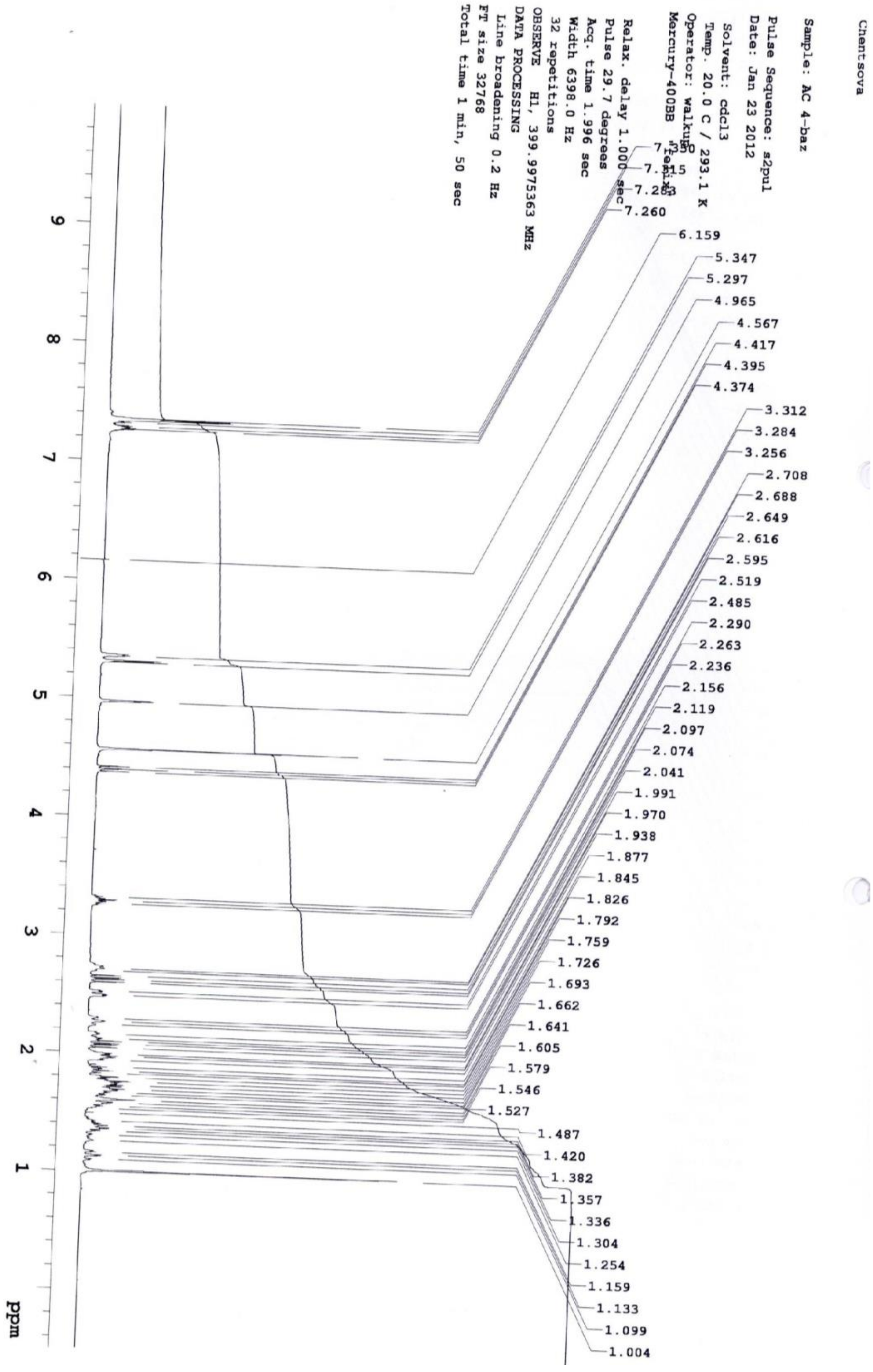

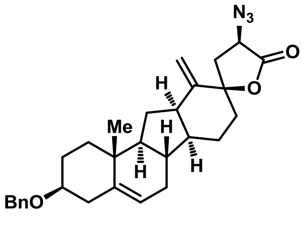

epi-14

^{13}C NMR (100 MHz, $CDCl_3$)

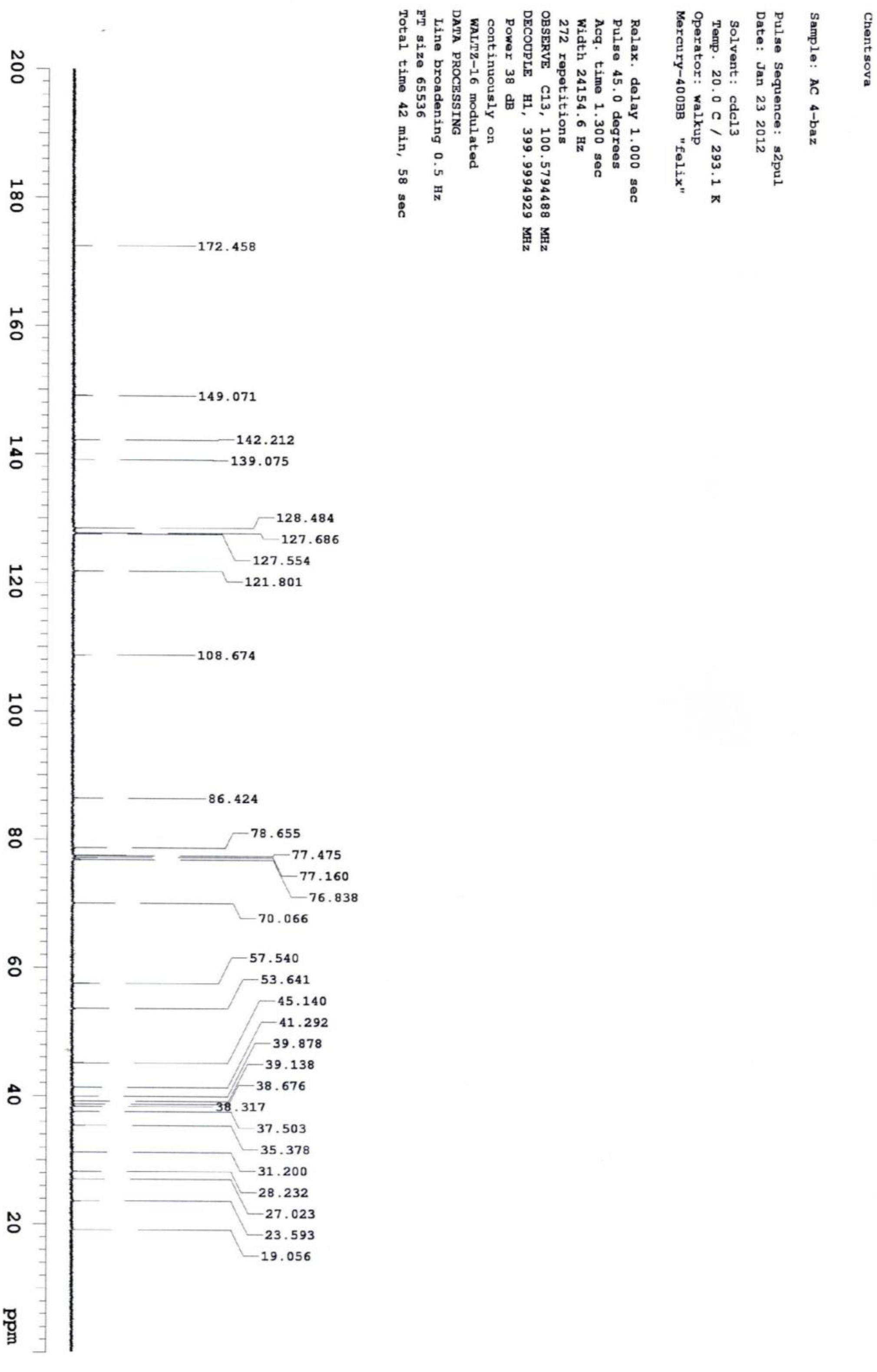

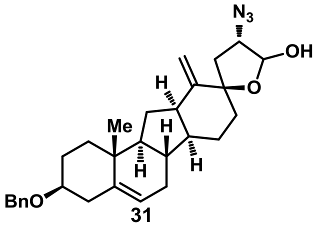

31

^{1}H NMR (400 MHz, CDCl$_3$)

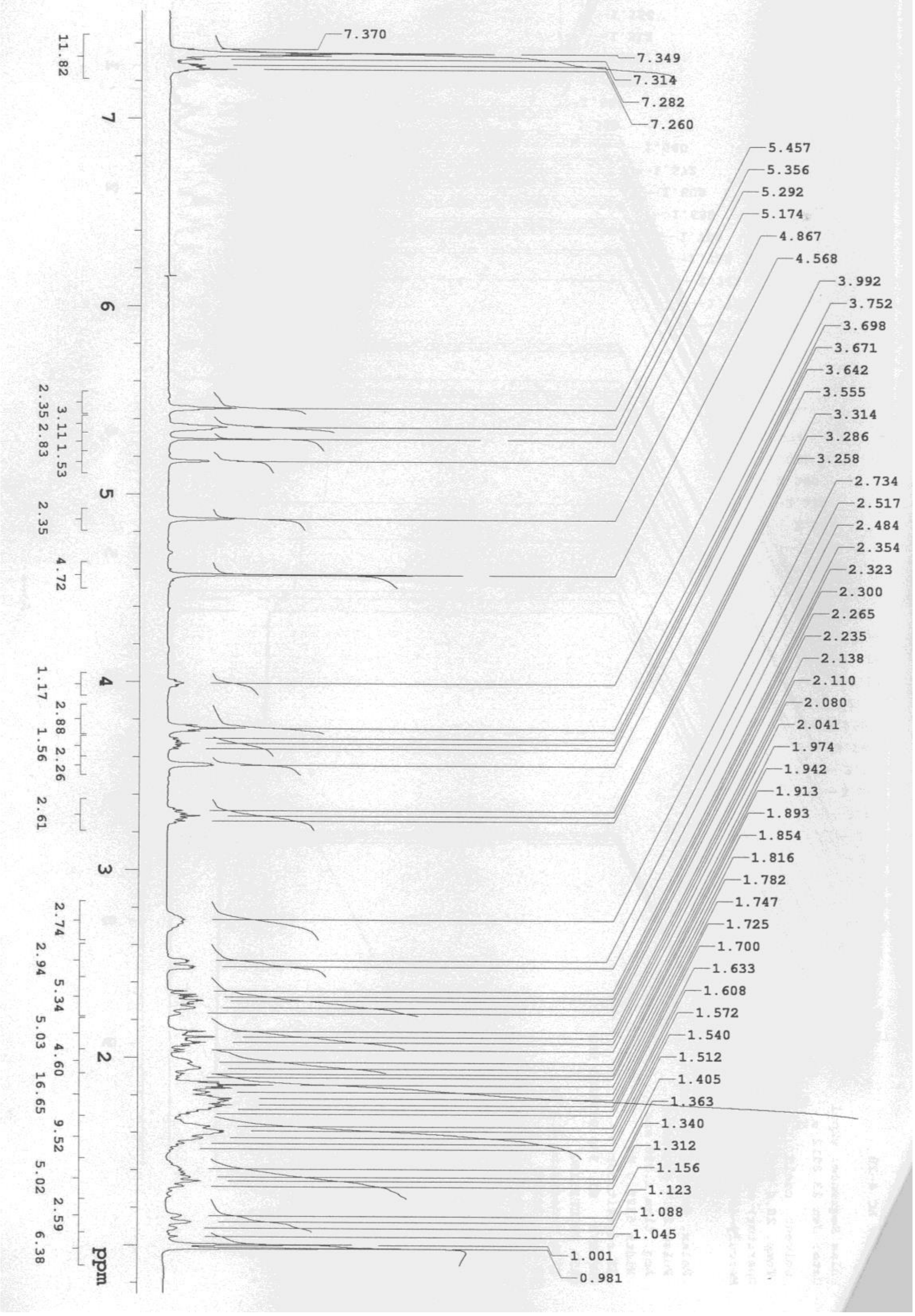

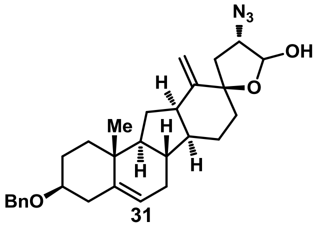

31

^{13}C NMR (100 MHz, $CDCl_3$)

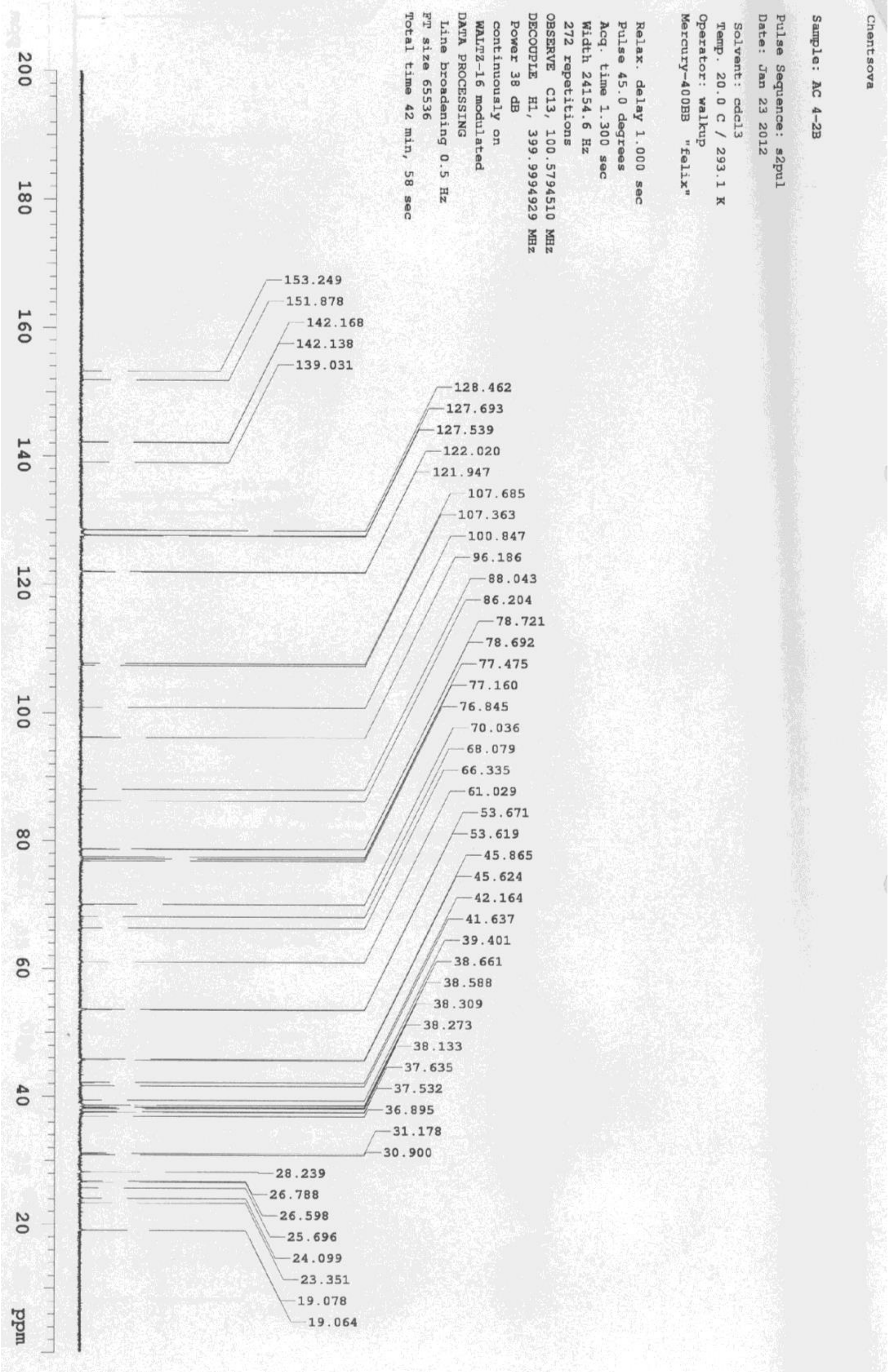

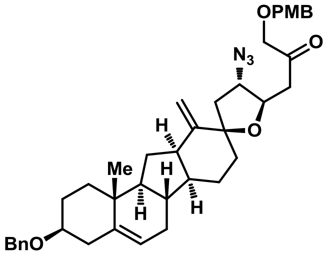

15

^{1}H NMR (300 MHz, $CDCl_3$)

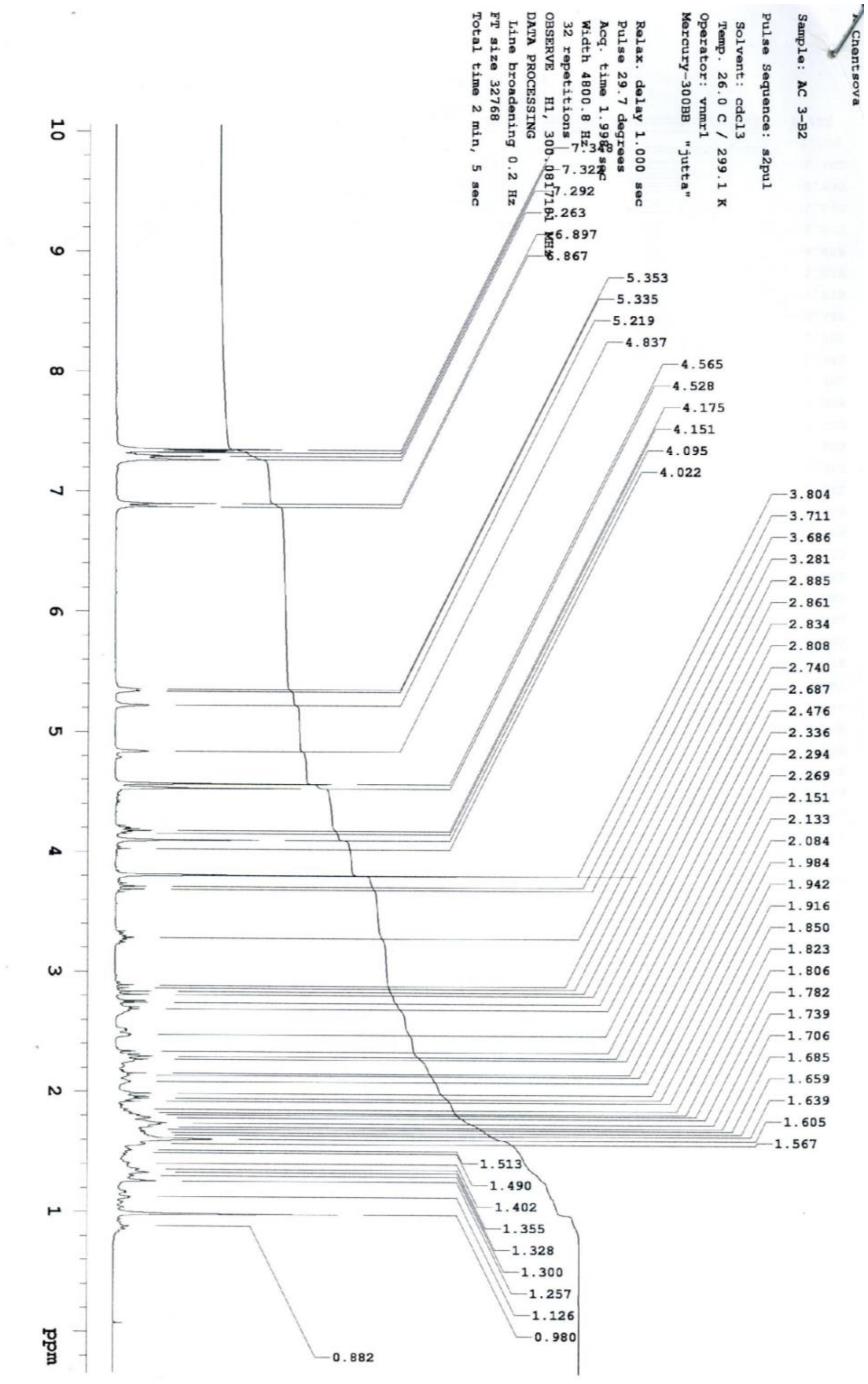

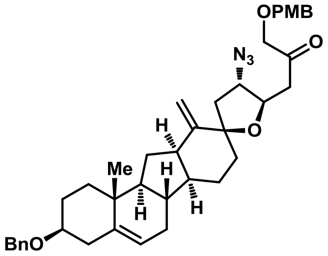

15

^{13}C NMR (100 MHz, $CDCl_3$)

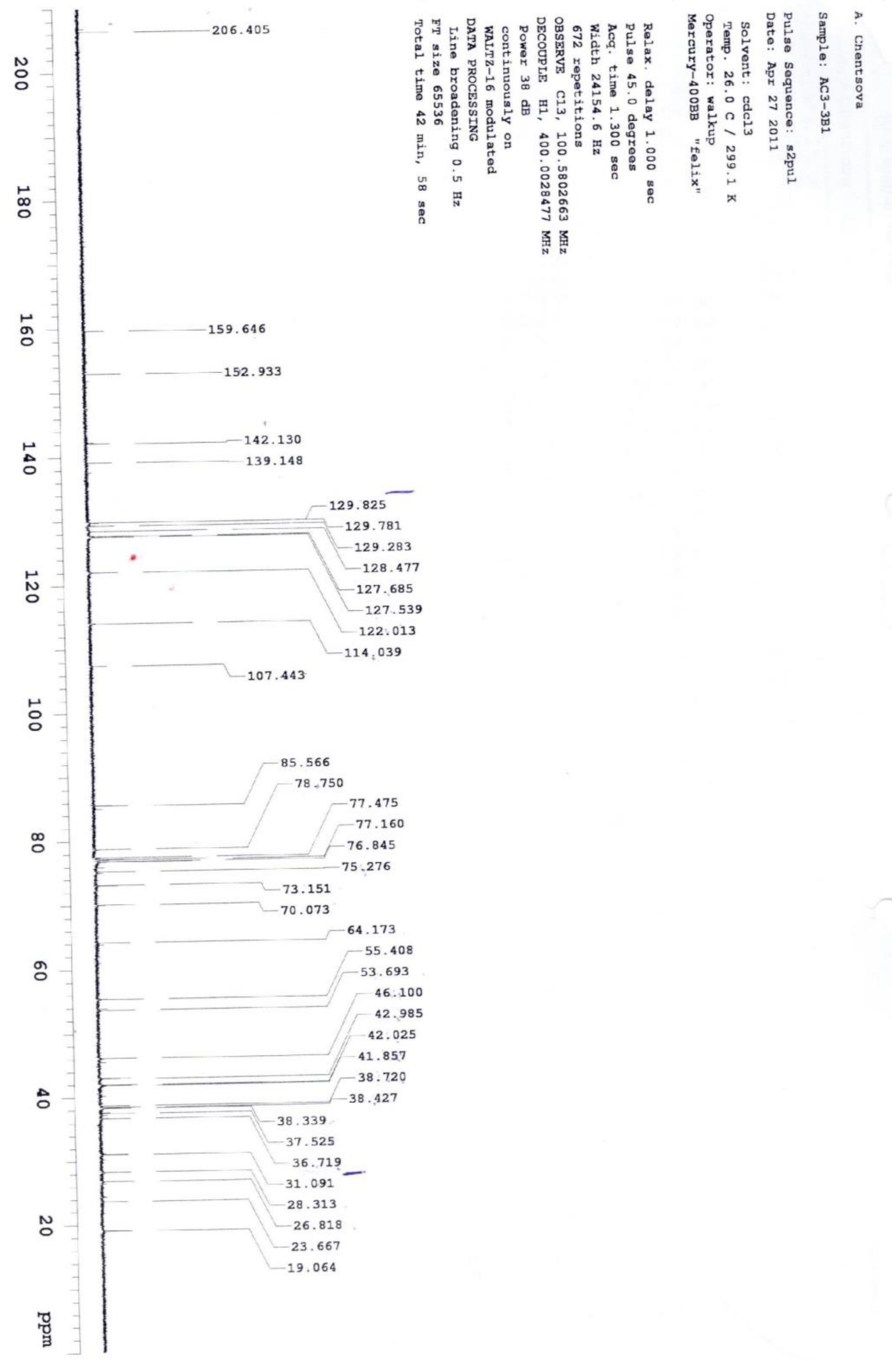
A. Chentsova
Sample: AC3-3B1
Pulse Sequence: s2pul
Date: Apr 27 2011
Solvent: cdcl3
Temp. 26.0 C / 299.1 K
Operator: walkup
Mercury-400BB "felix"
Relax. delay 1.000 sec
Pulse 45.0 degrees
Acq. time 1.300 sec
Width 24154.6 Hz
672 repetitions
OBSERVE C13, 100.5802663 MHz
DECOUPLE H1, 400.0028477 MHz
Power 38 dB
continuously on
WALTZ-16 modulated
DATA PROCESSING
Line broadening 0.5 Hz
FT size 65536
Total time 42 min, 58 sec
206.405
159.646
152.933
142.130
139.148
129.825
129.781
129.283
128.477
127.685
127.539
122.013
114.039
107.443
85.566
78.750
77.475
77.160
76.845
75.276
73.151
70.073
64.173
55.408
53.693
46.100
42.985
42.025
41.857
38.720
38.427
38.339
37.525
36.719
31.091
28.313
26.818
23.667
19.064
200
180
160
140
120
100
80
60
40
20
ppm

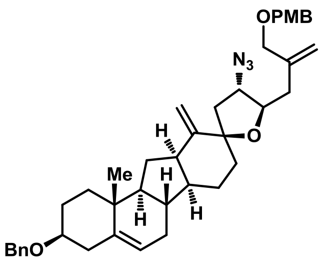
OPMB
N_3
H
Me
H
H
H
O
BnO

16

^{1}H NMR (400 MHz, $CDCl_3$)

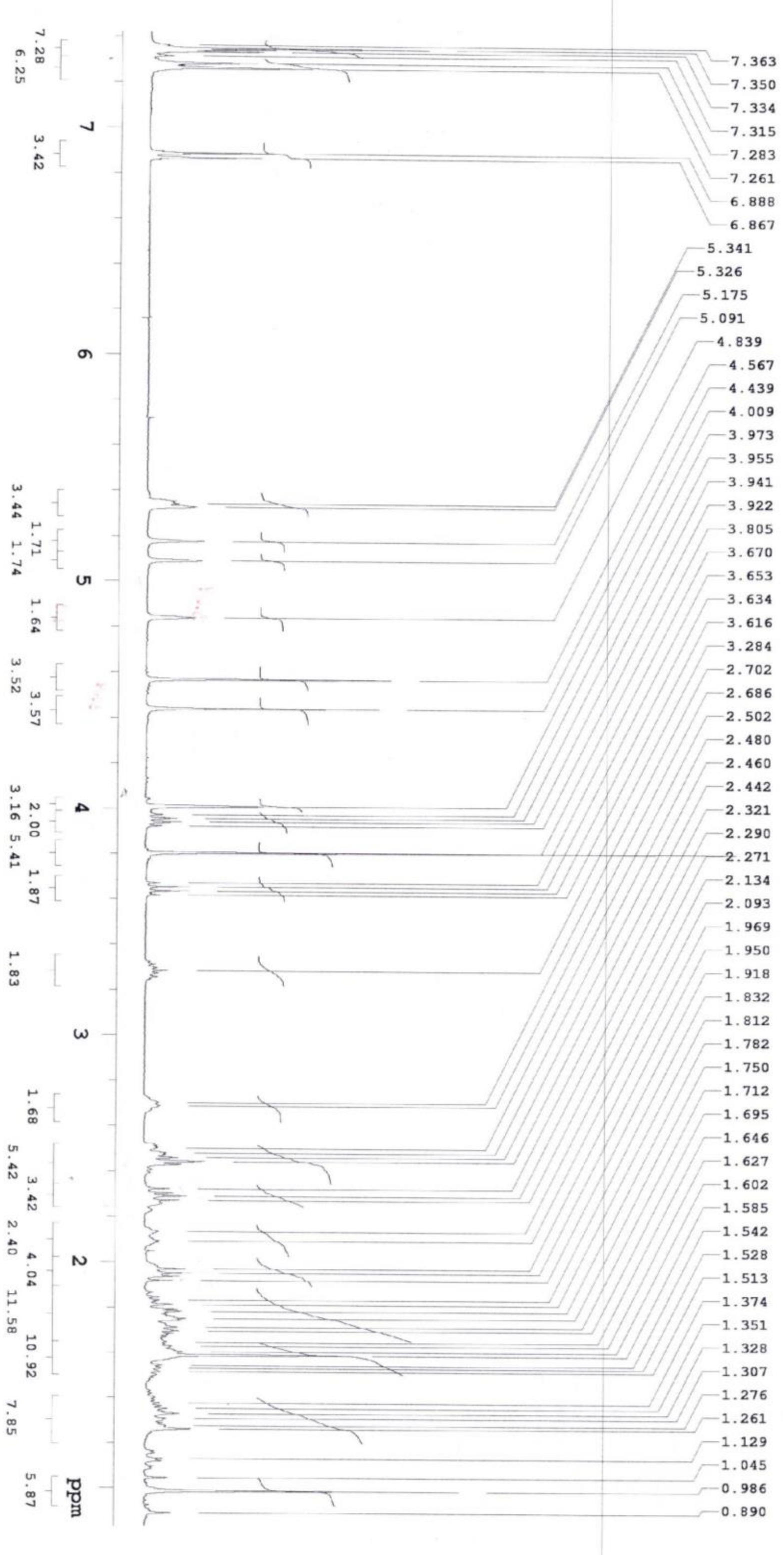

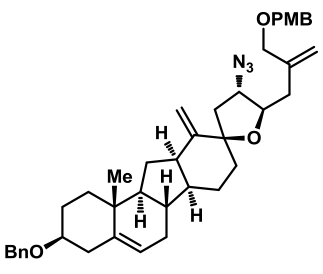

16

APT (100 MHz, $CDCl_3$)

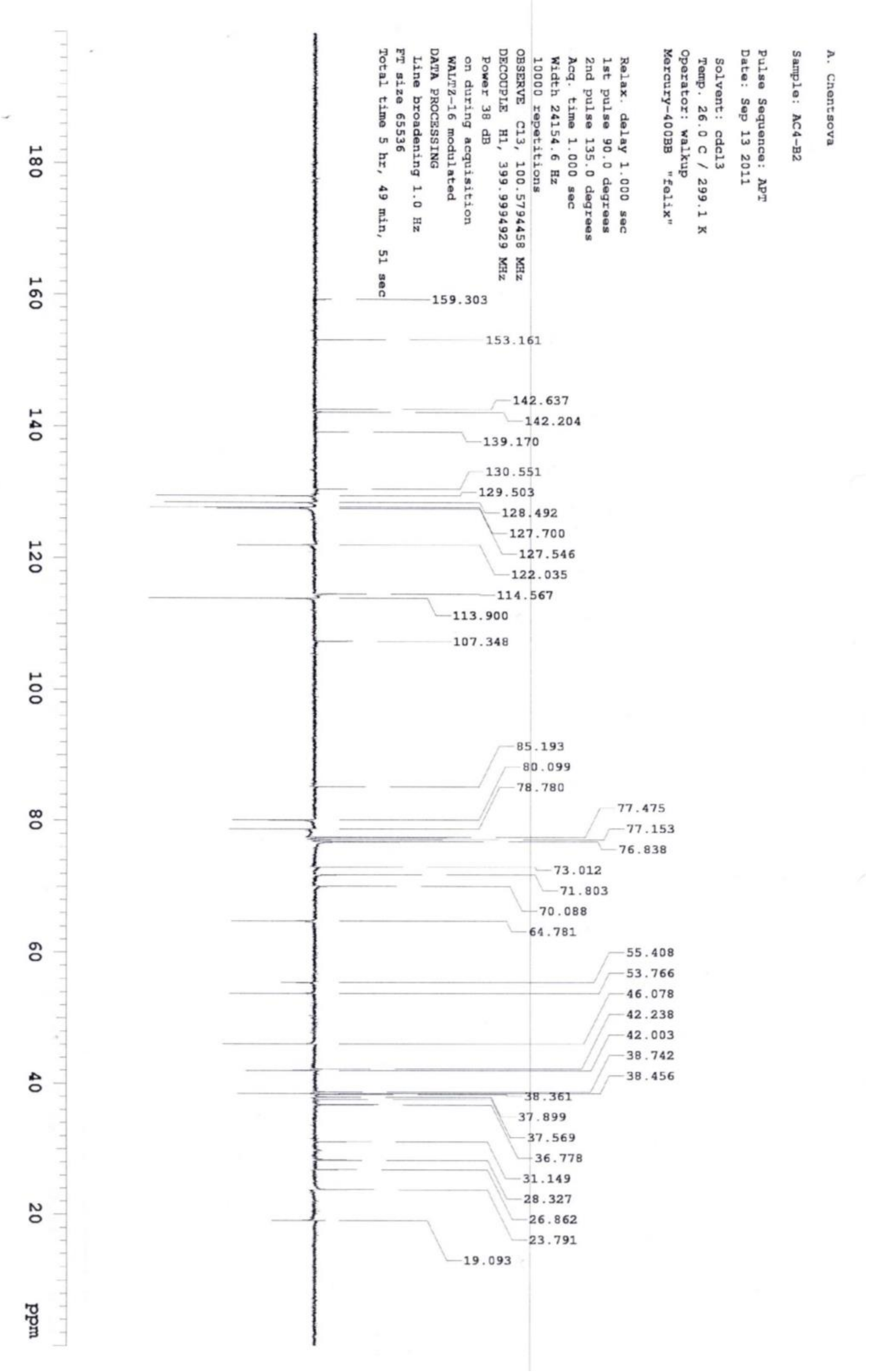

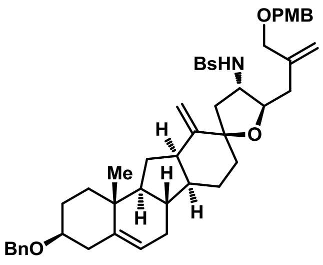

17

^{1}H NMR (400 MHz, $CDCl_3$)

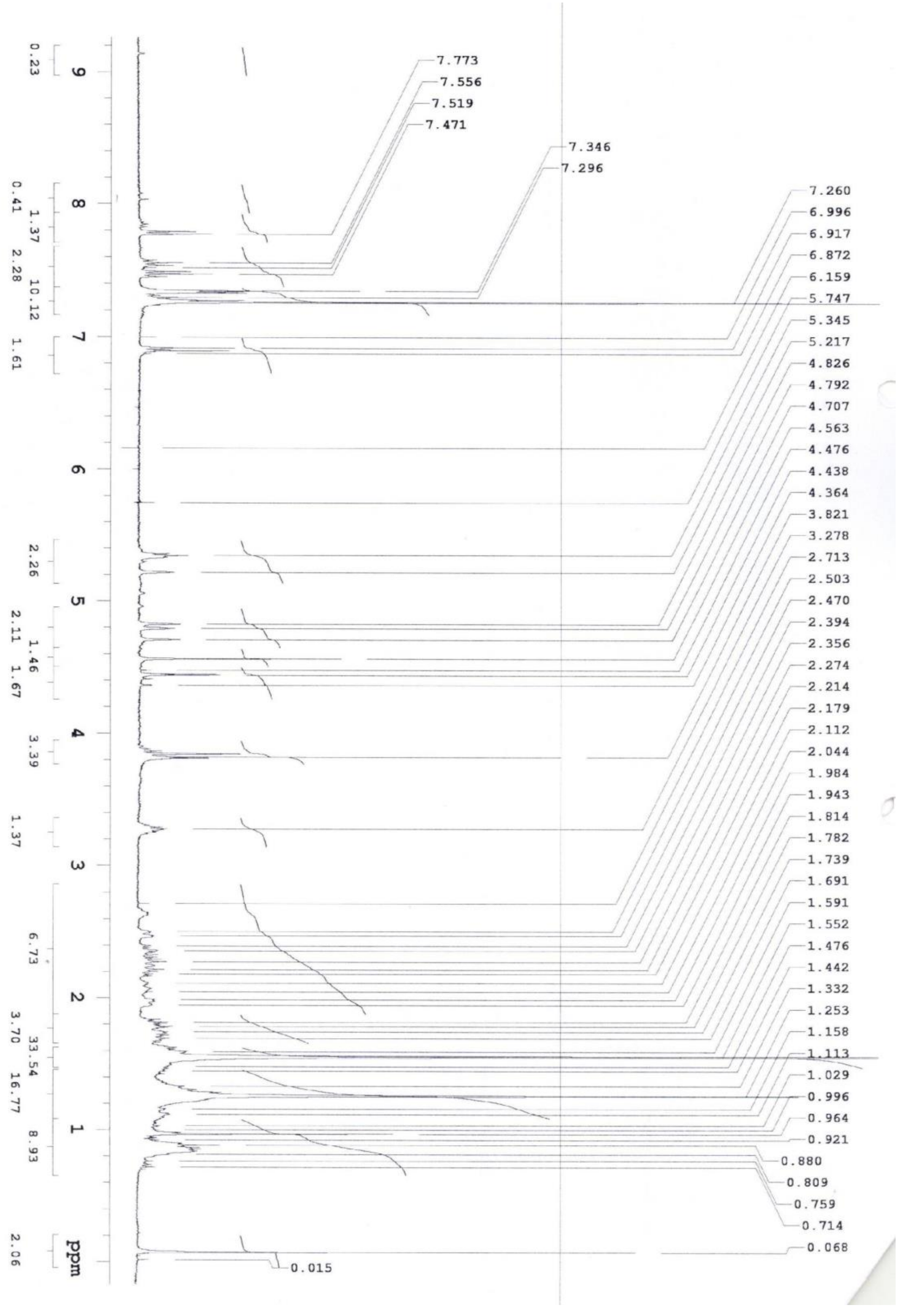

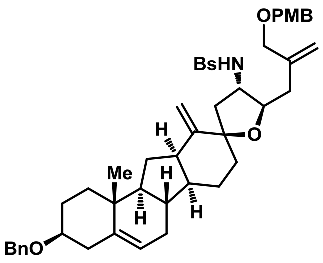

17

^{13}C NMR (75 MHz, $CDCl_3$)

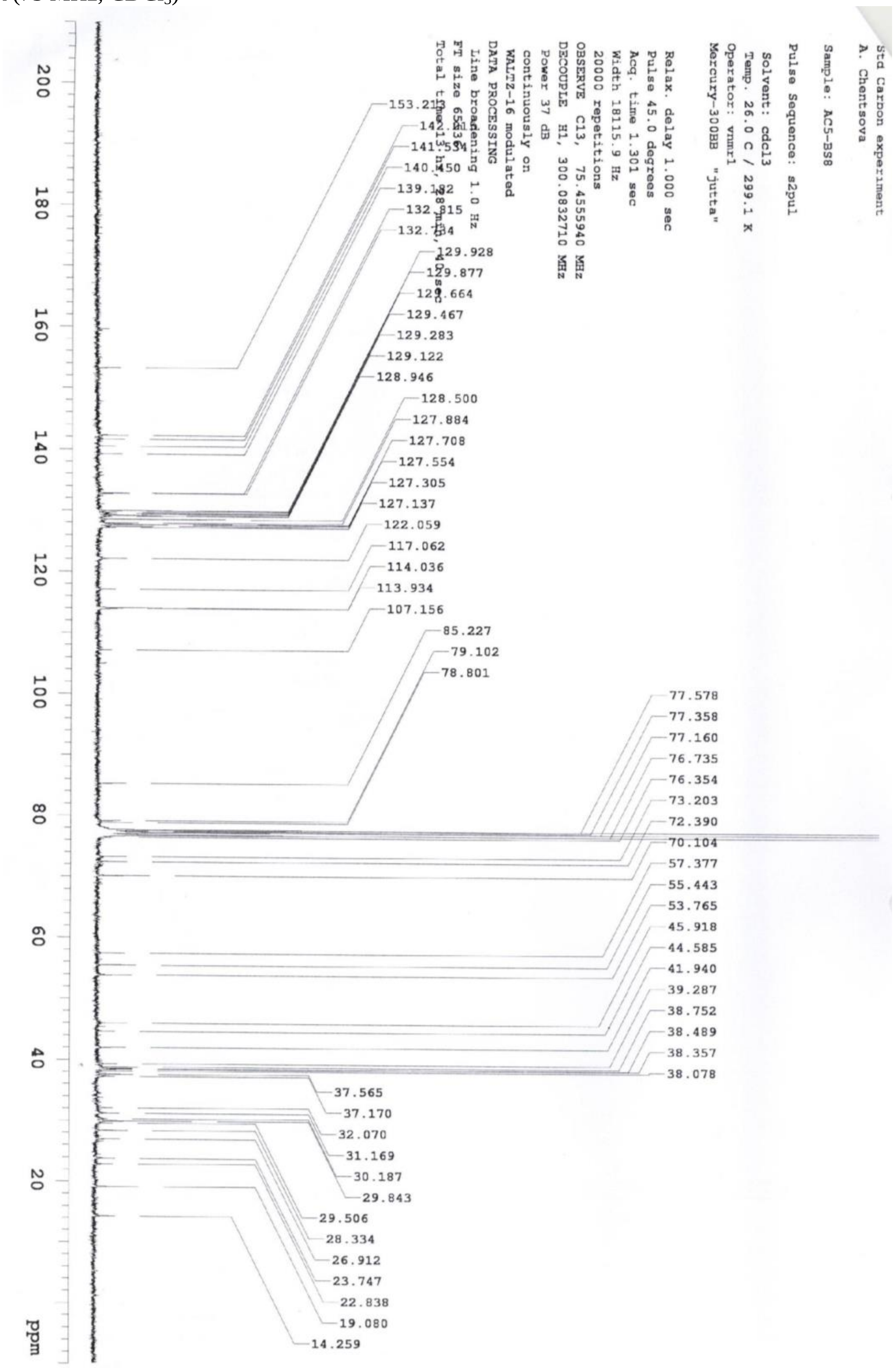

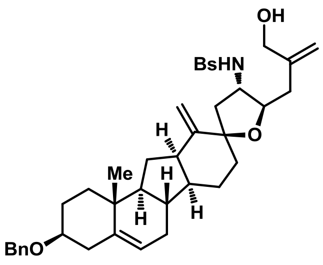

32

^{1}H NMR (400 MHz, $CDCl_3$)

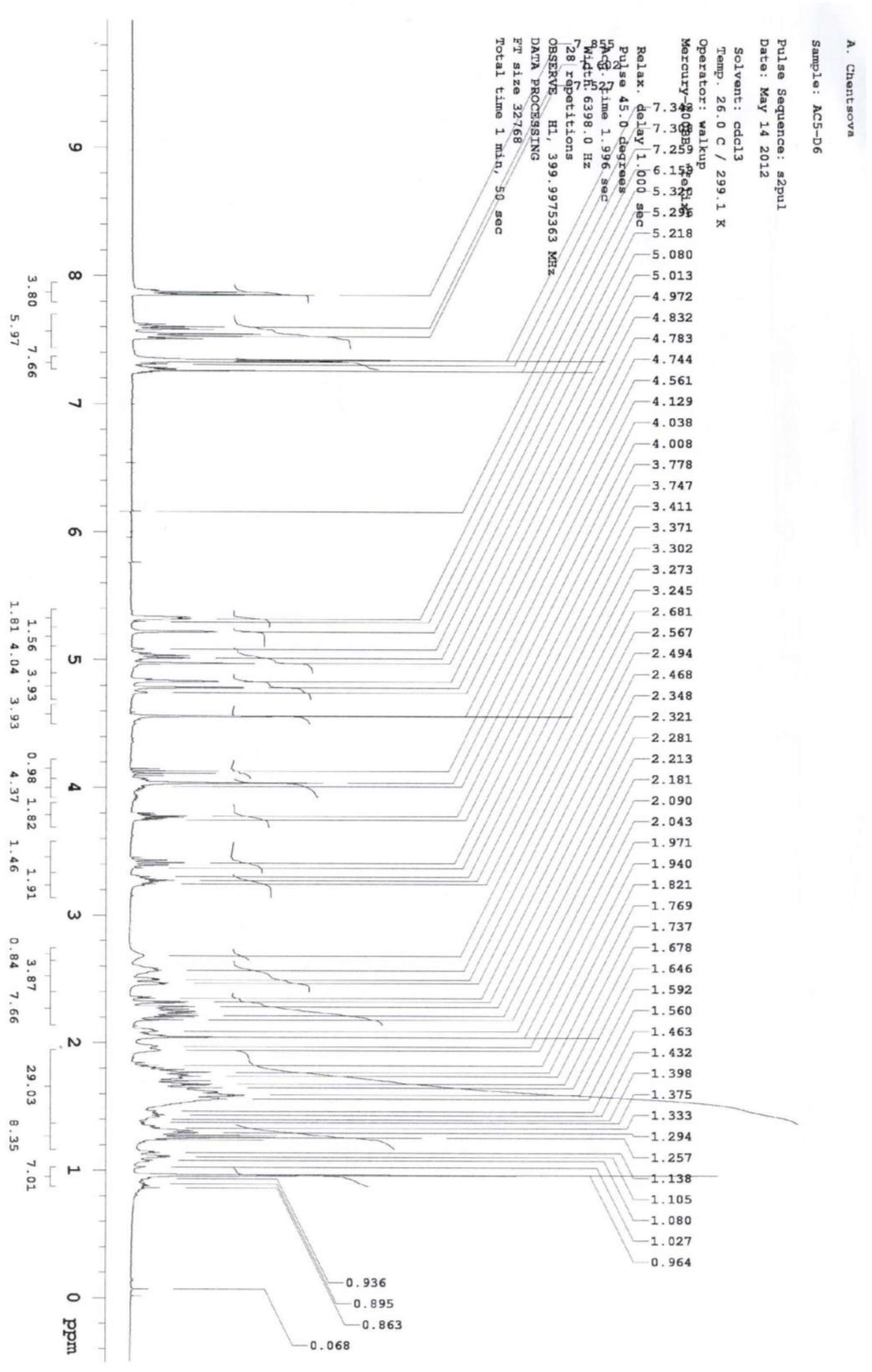

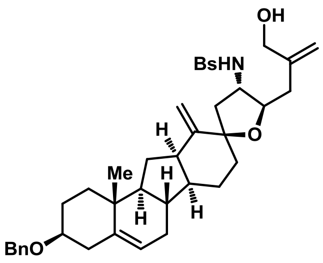

32

APT (75 MHz, $CDCl_3$)

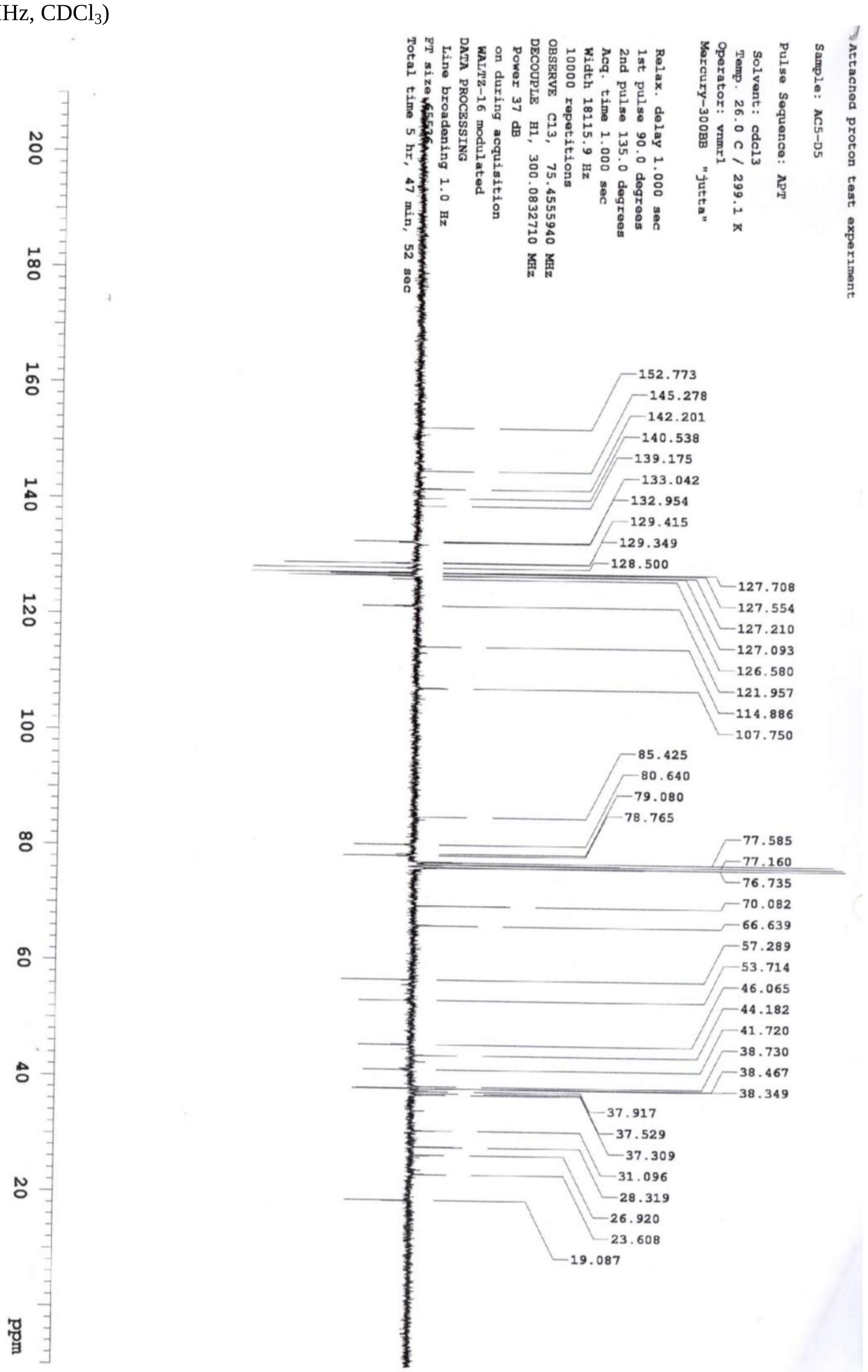

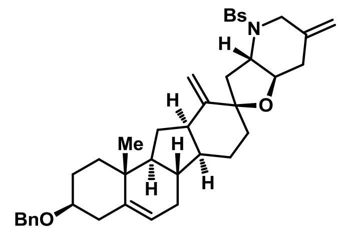

18

^{1}H NMR (300 MHz, $CDCl_3$)

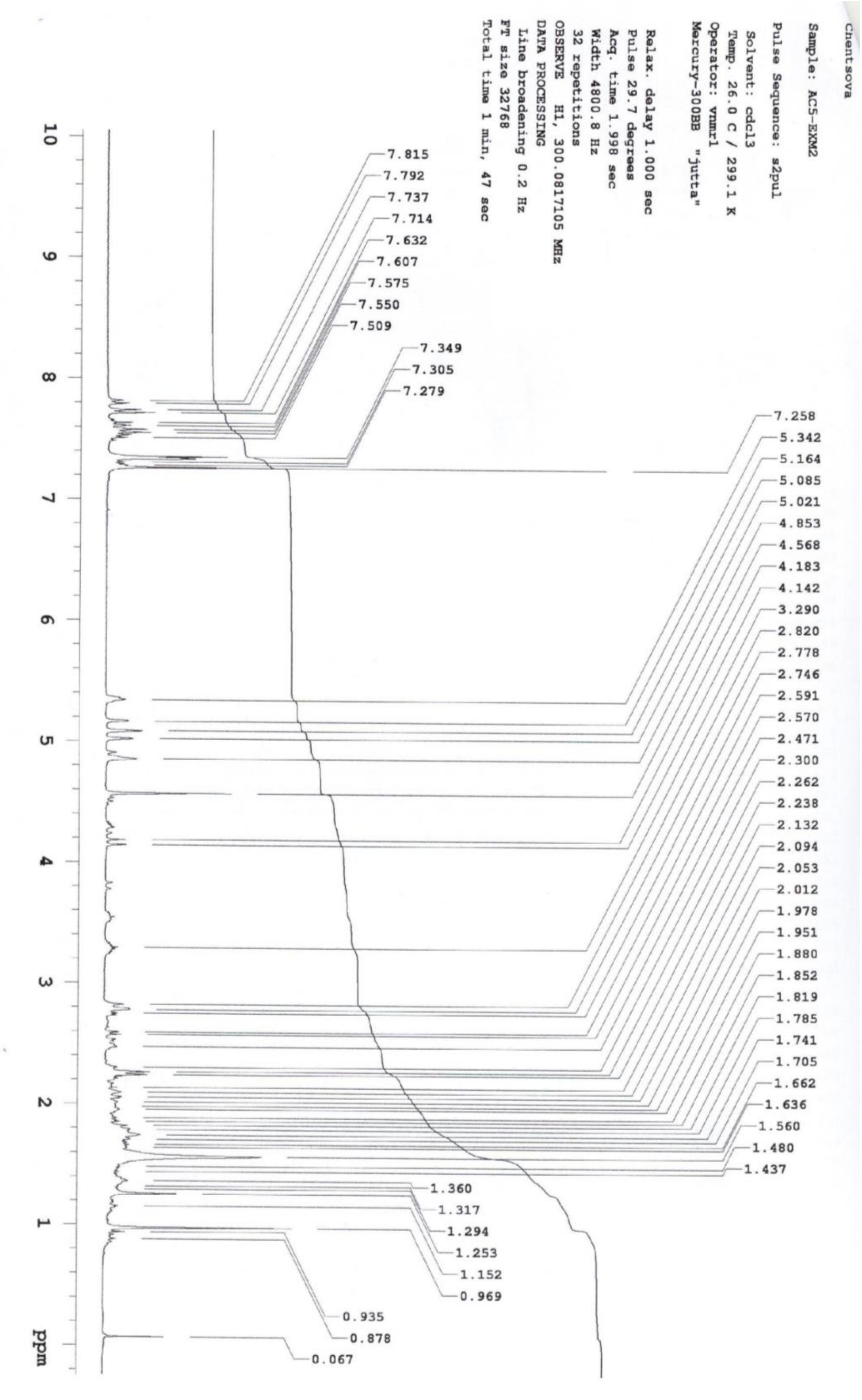

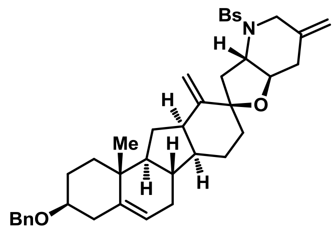

18

APT (100 MHz, $CDCl_3$)

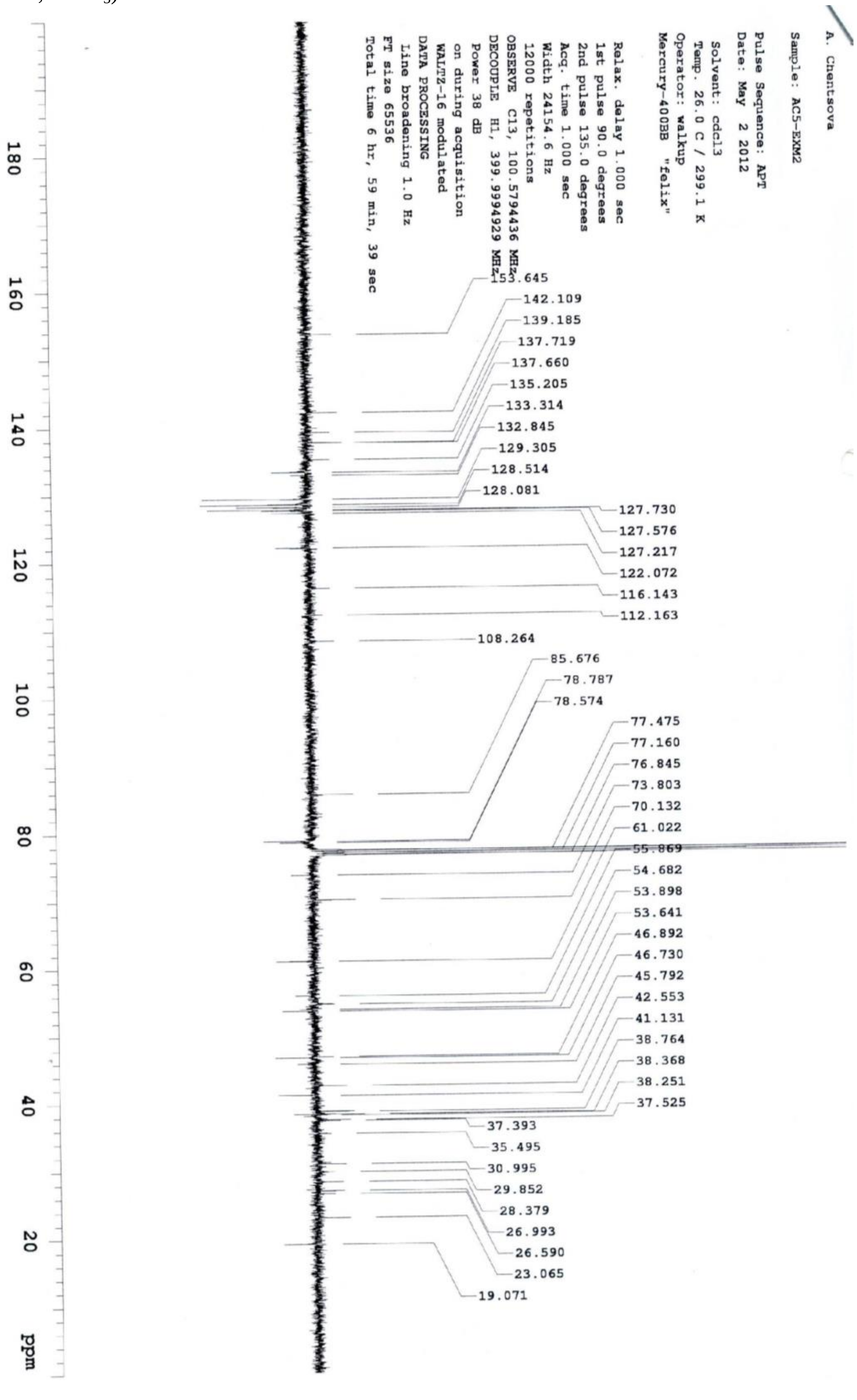

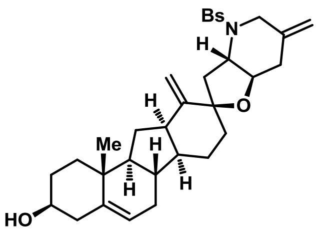

33

^{1}H NMR (400 MHz, $CDCl_3$)

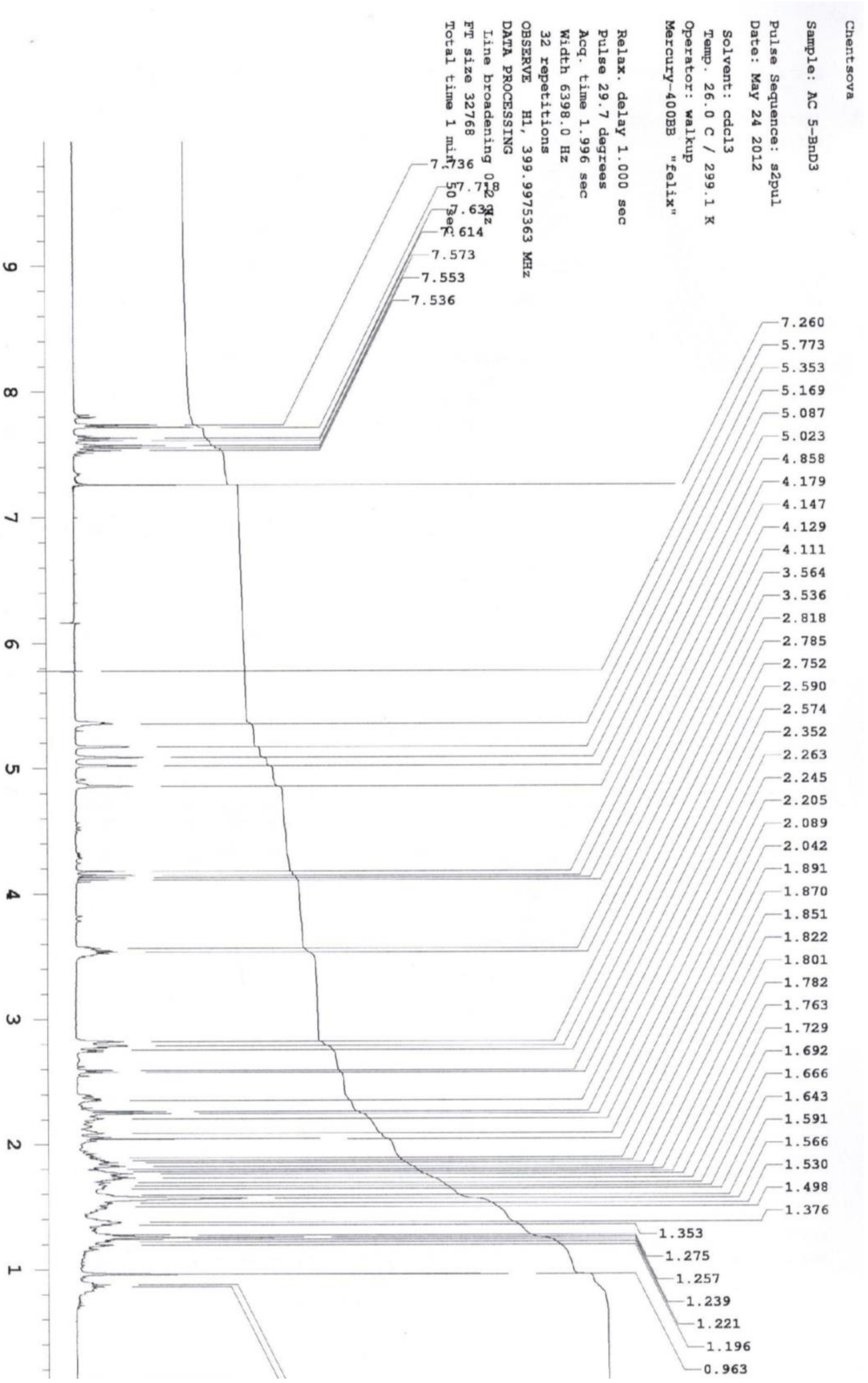

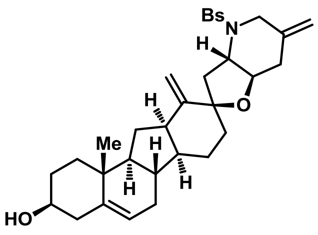

33

^{13}C NMR (100 MHz, $CDCl_3$)

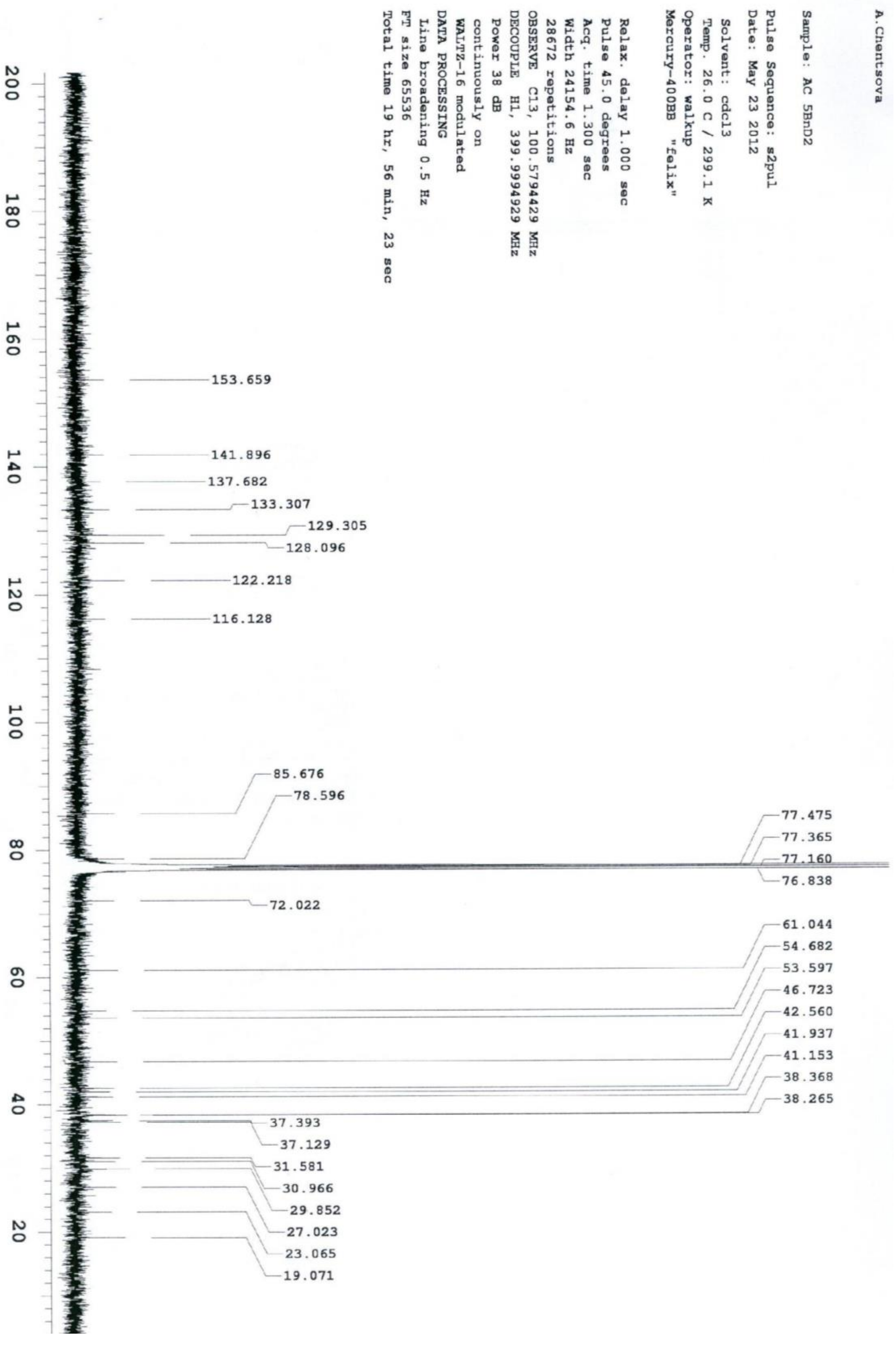

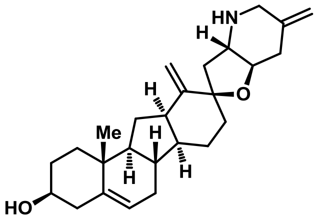

19

^{1}H NMR (400 MHz, $CDCl_3$)

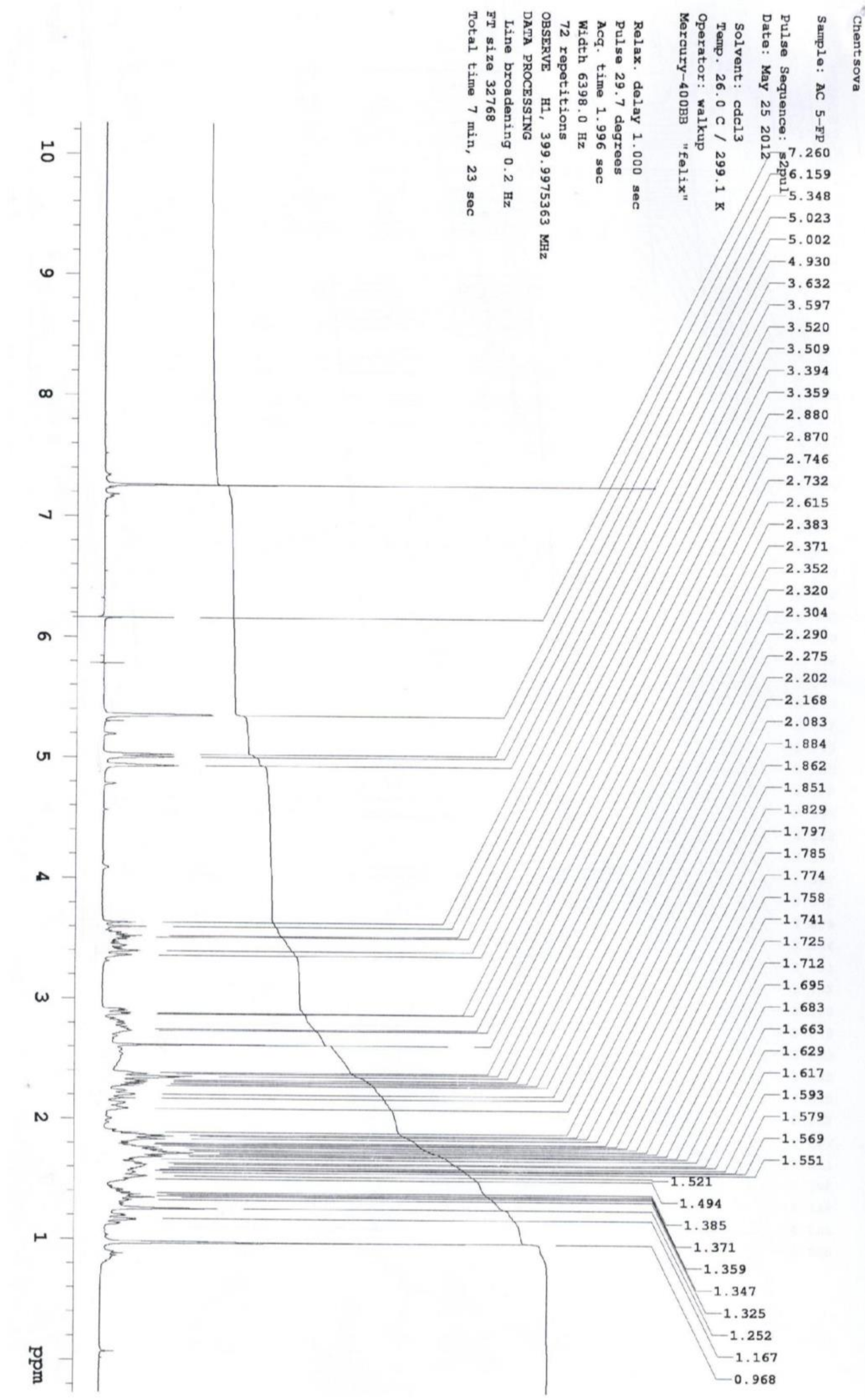

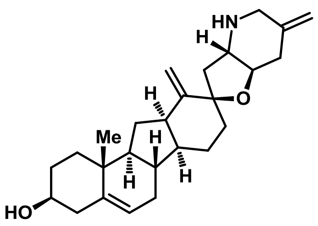

19

^{13}C NMR (175 MHz, $CDCl_3$)

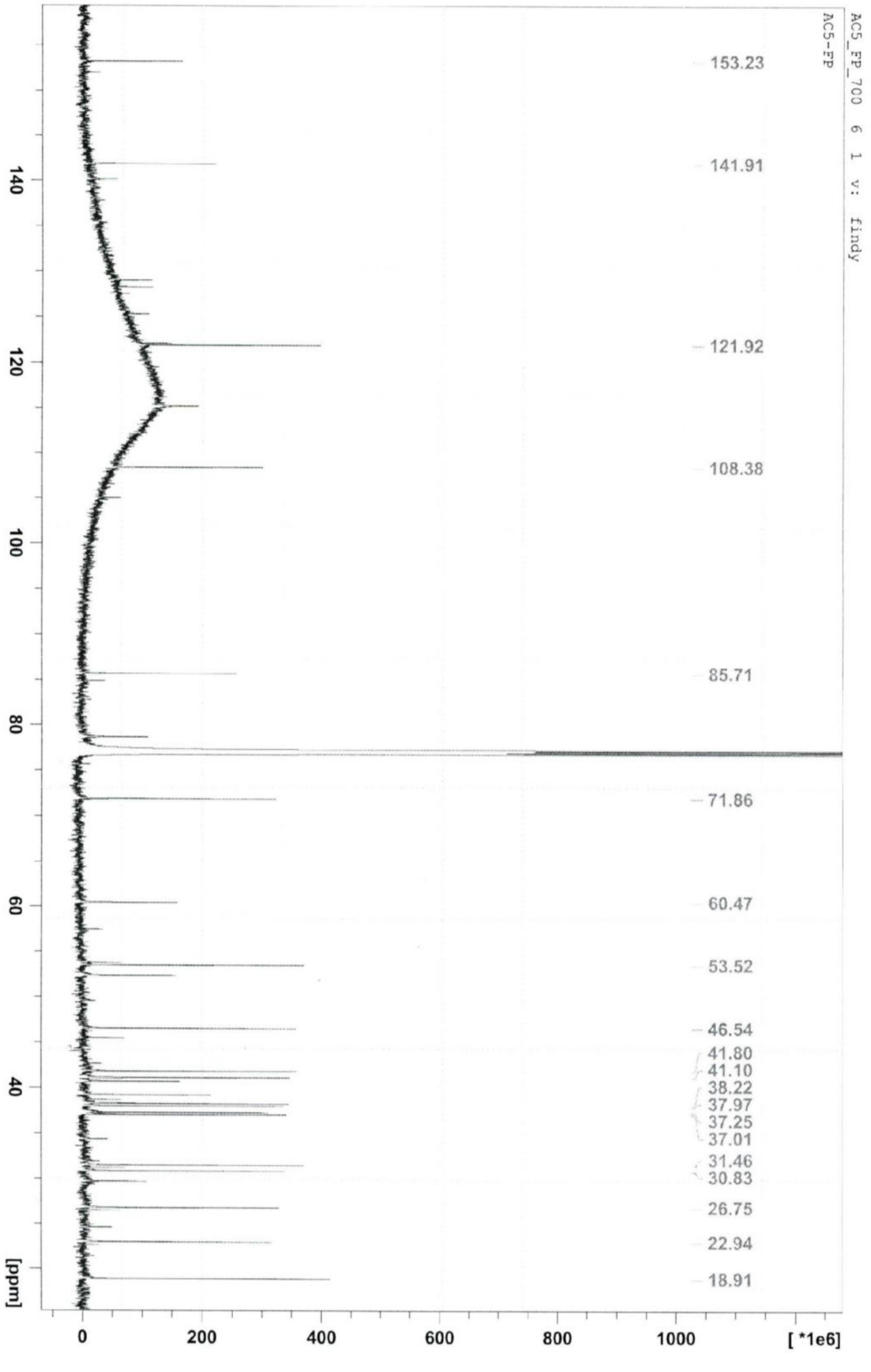

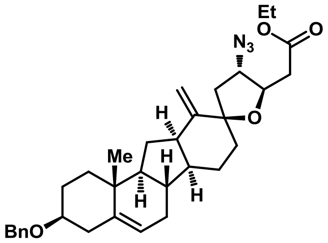

20

^{1}H NMR (400 MHz, $CDCl_3$)

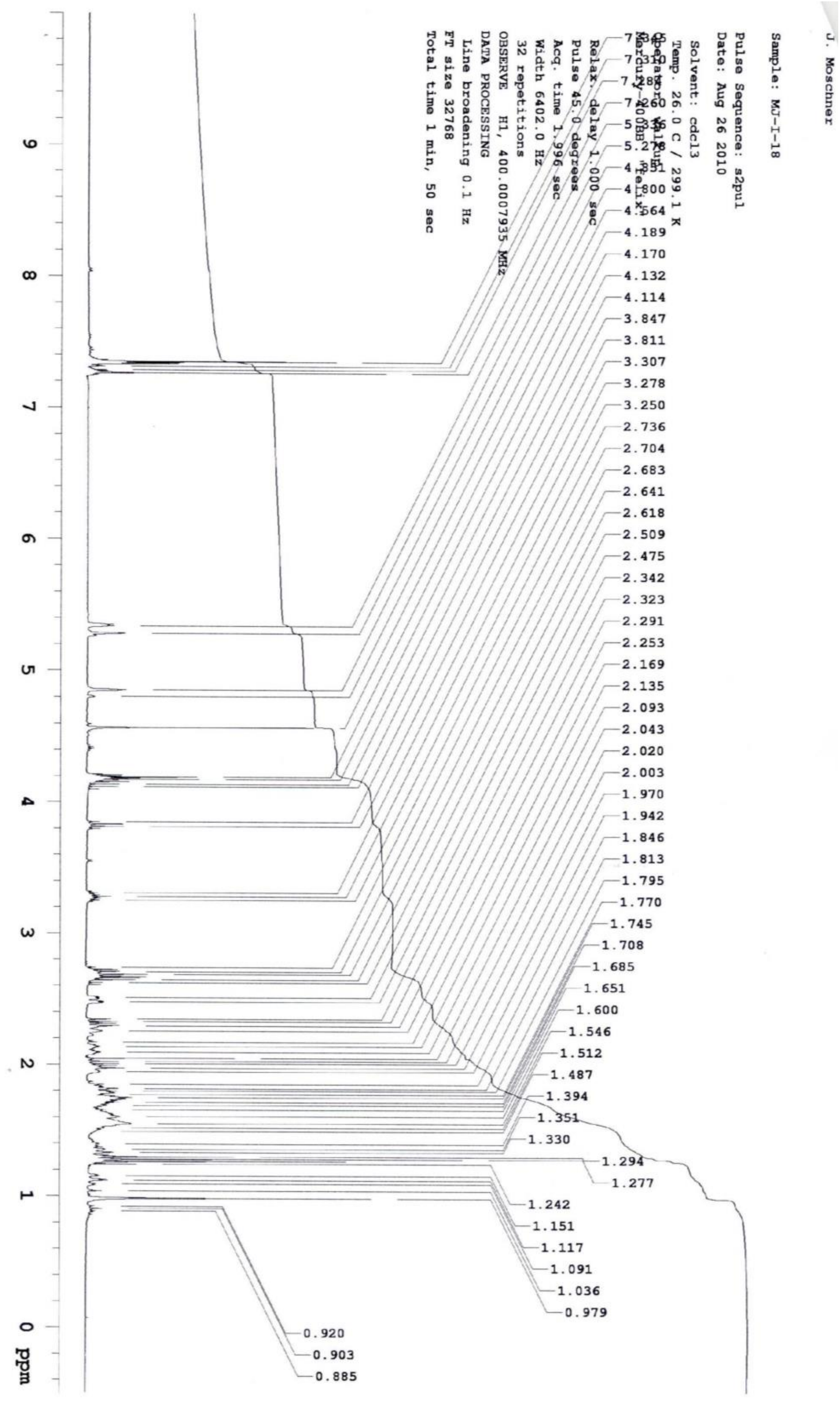

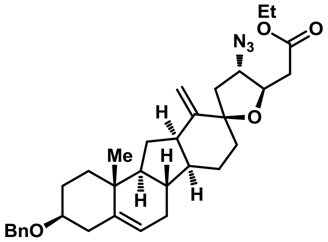

20

^{13}C NMR (75 MHz, $CDCl_3$)

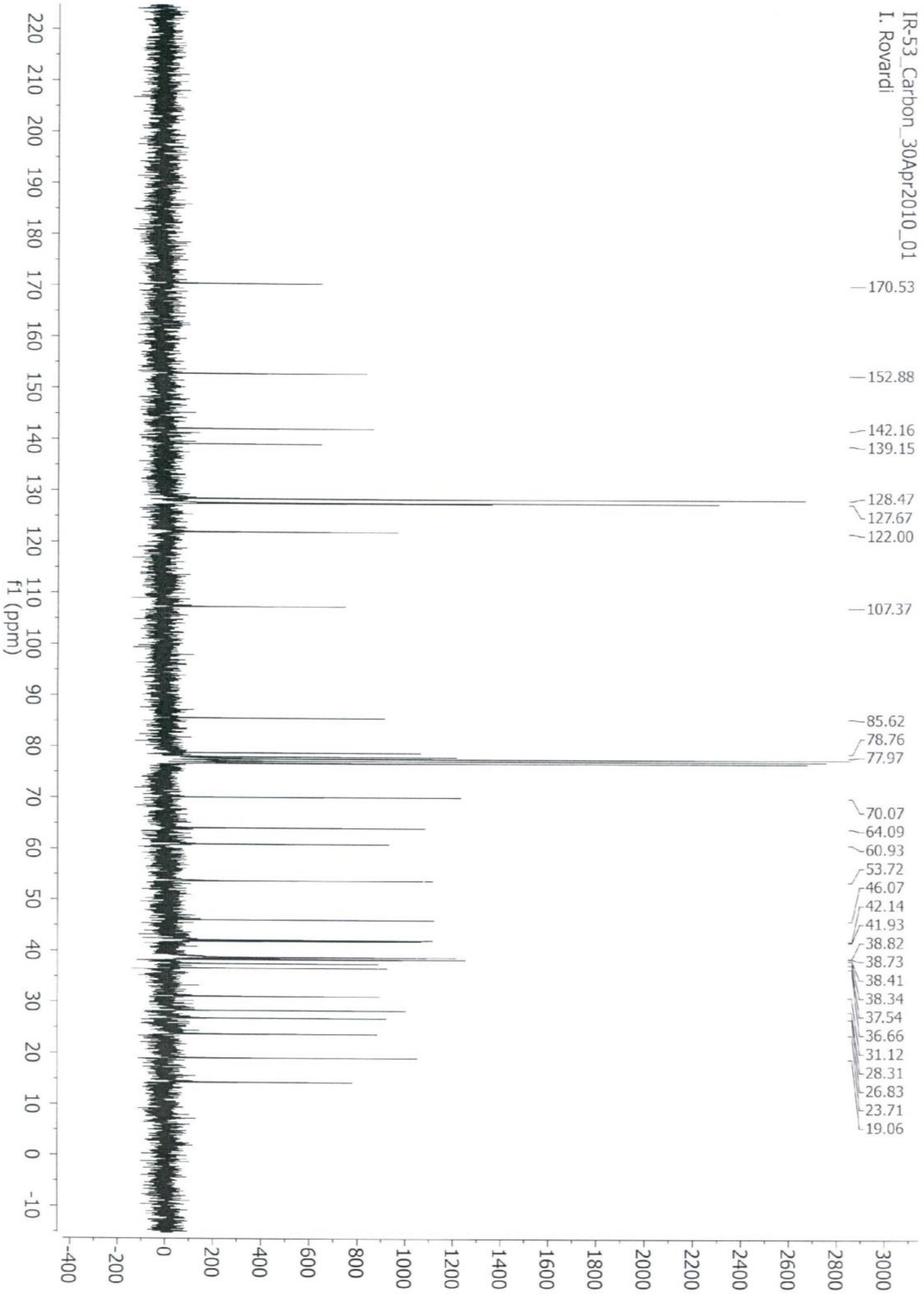

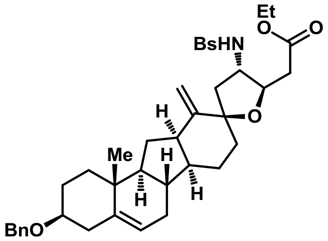

21

^{1}H NMR (300 MHz, $CDCl_3$)

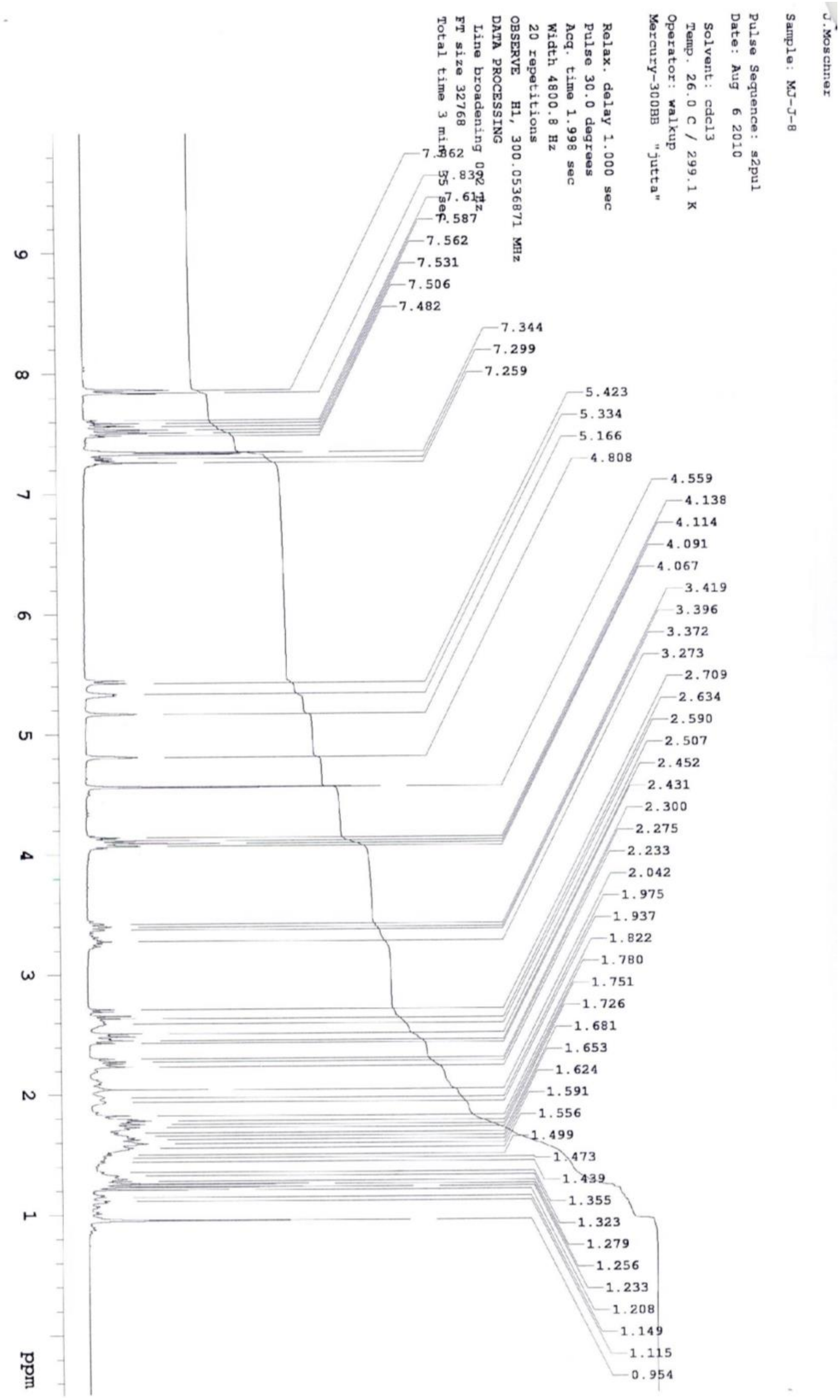

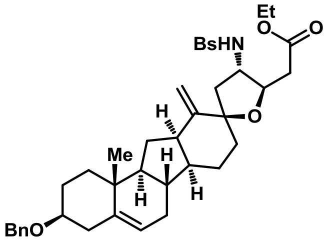

21

^{13}C NMR (75 MHz, $CDCl_3$)

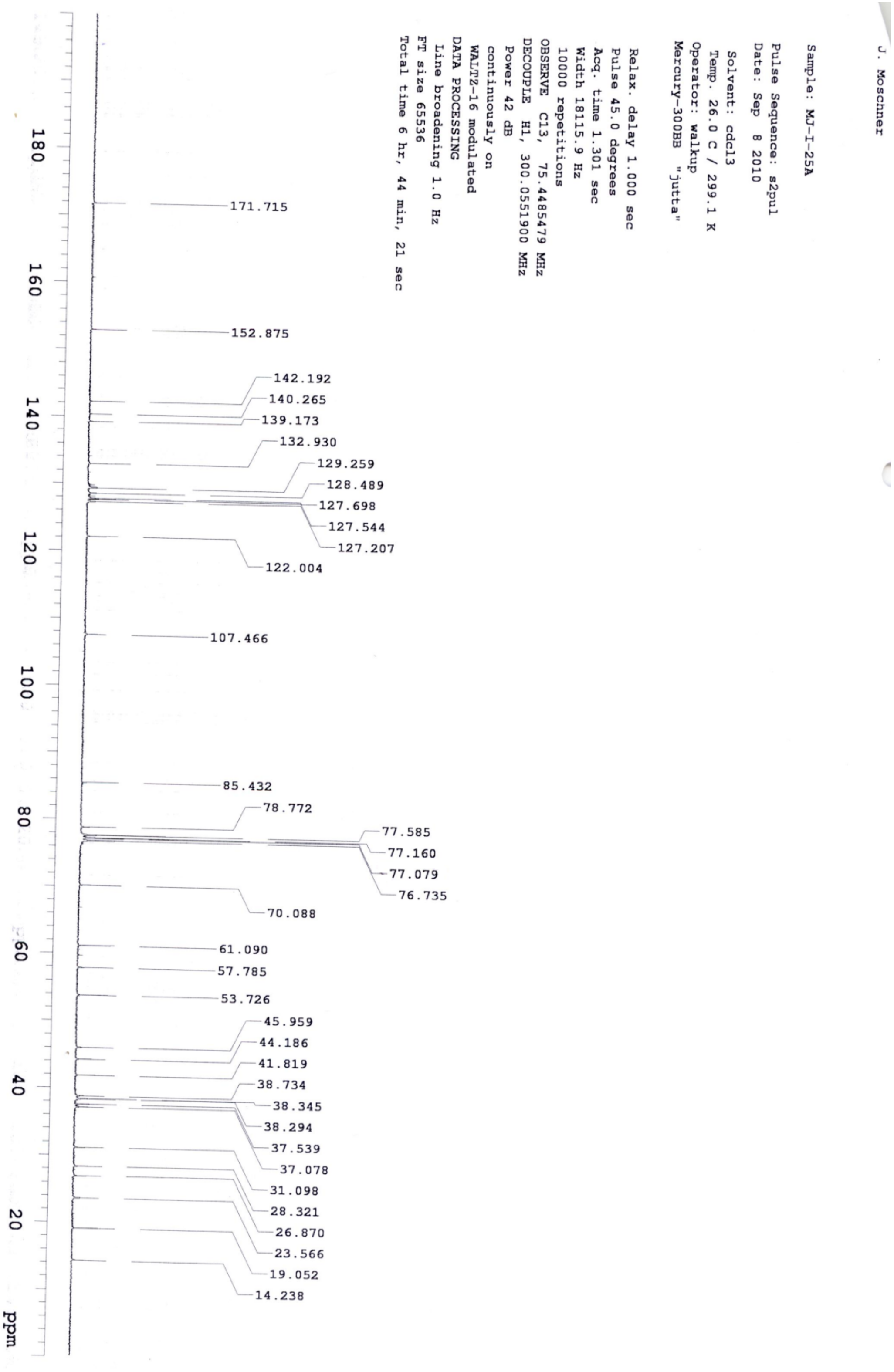

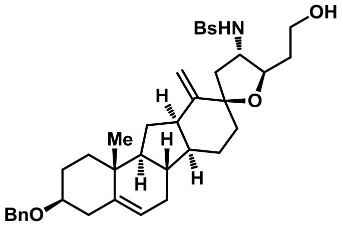

34

^{1}H NMR (300 MHz, $CDCl_3$)

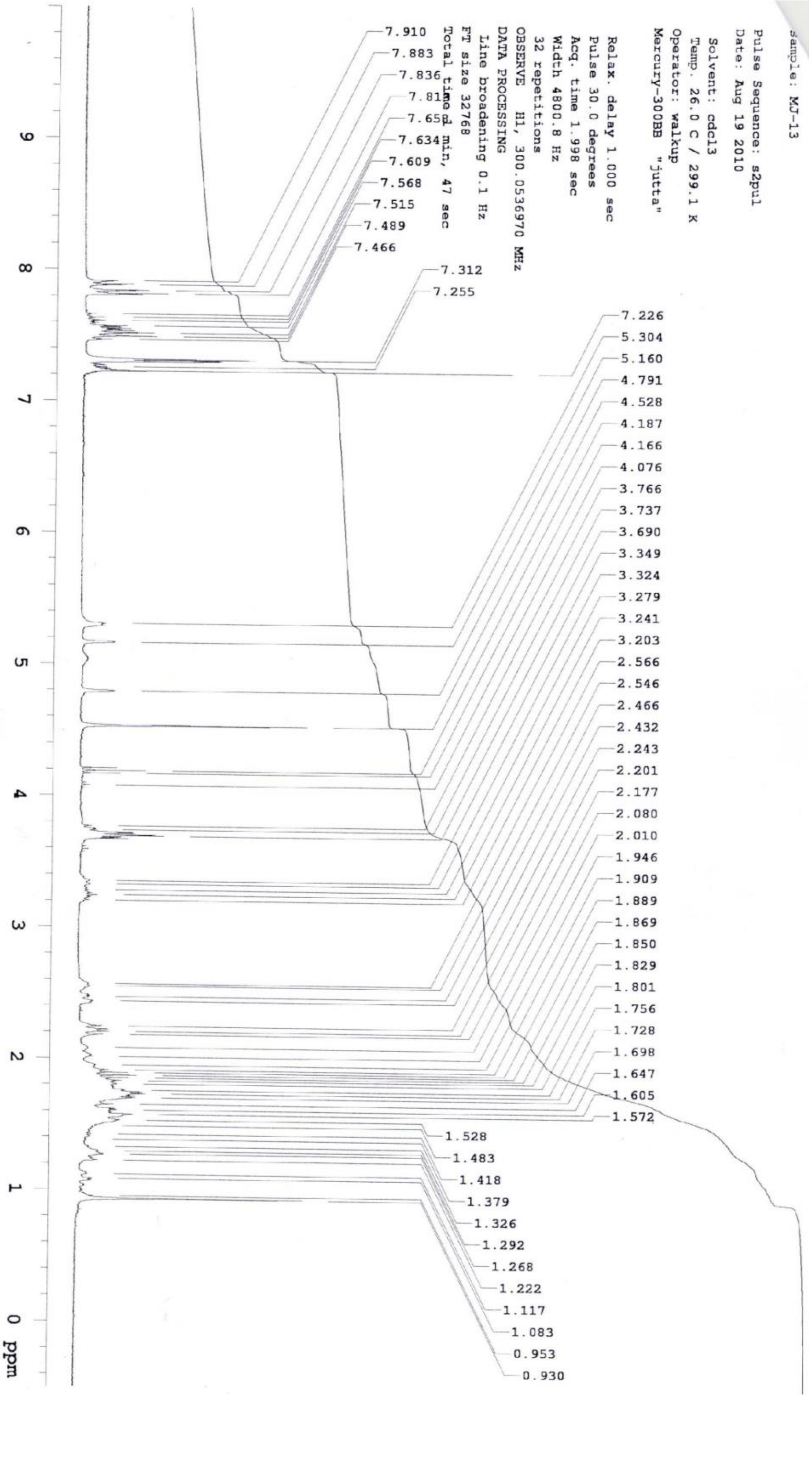

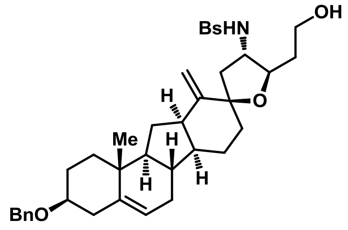

34

^{13}C NMR (100 MHz, $CDCl_3$)

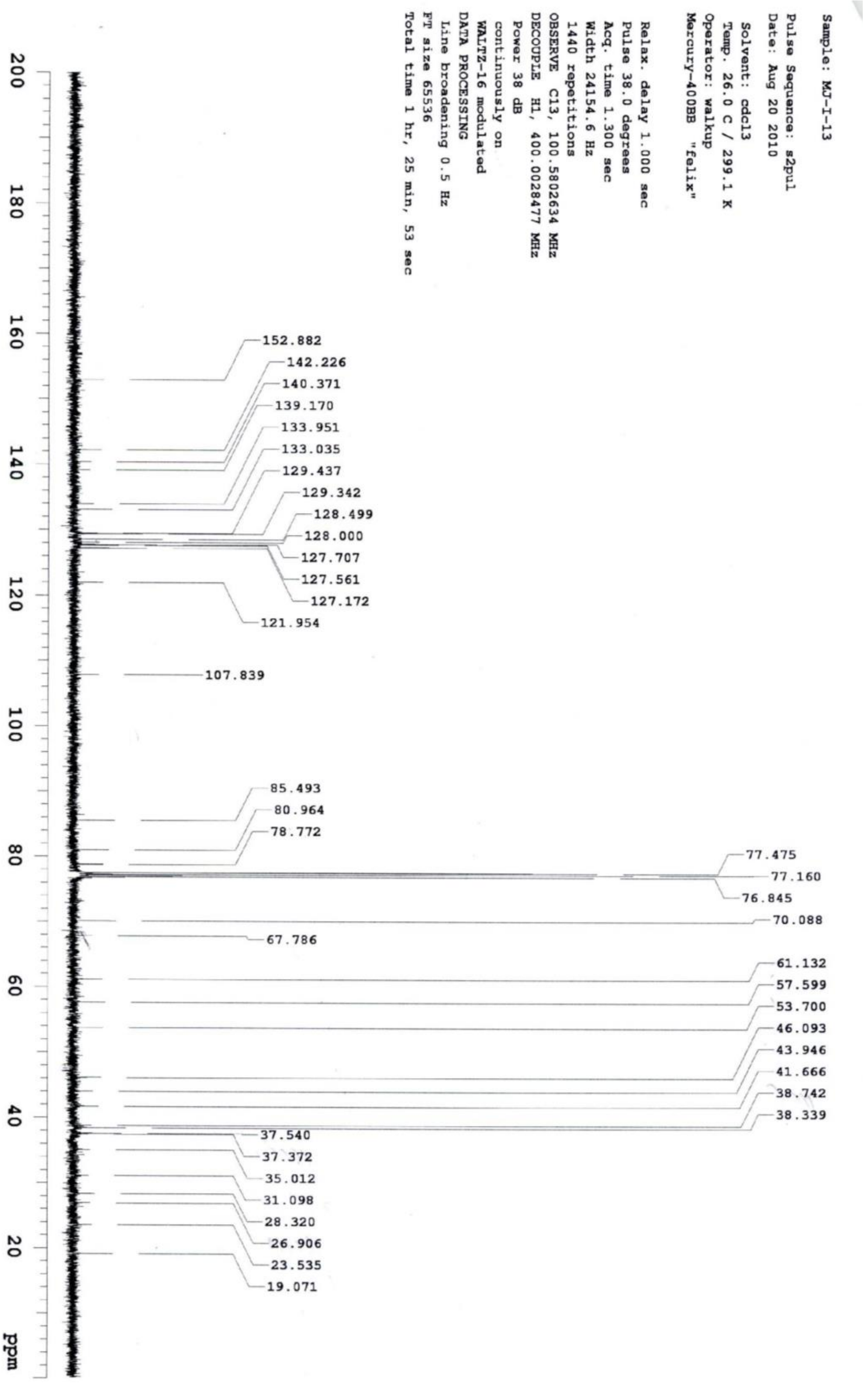

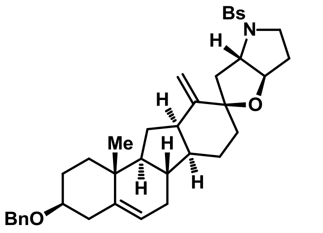

22

^{1}H NMR (400 MHz, $CDCl_3$)

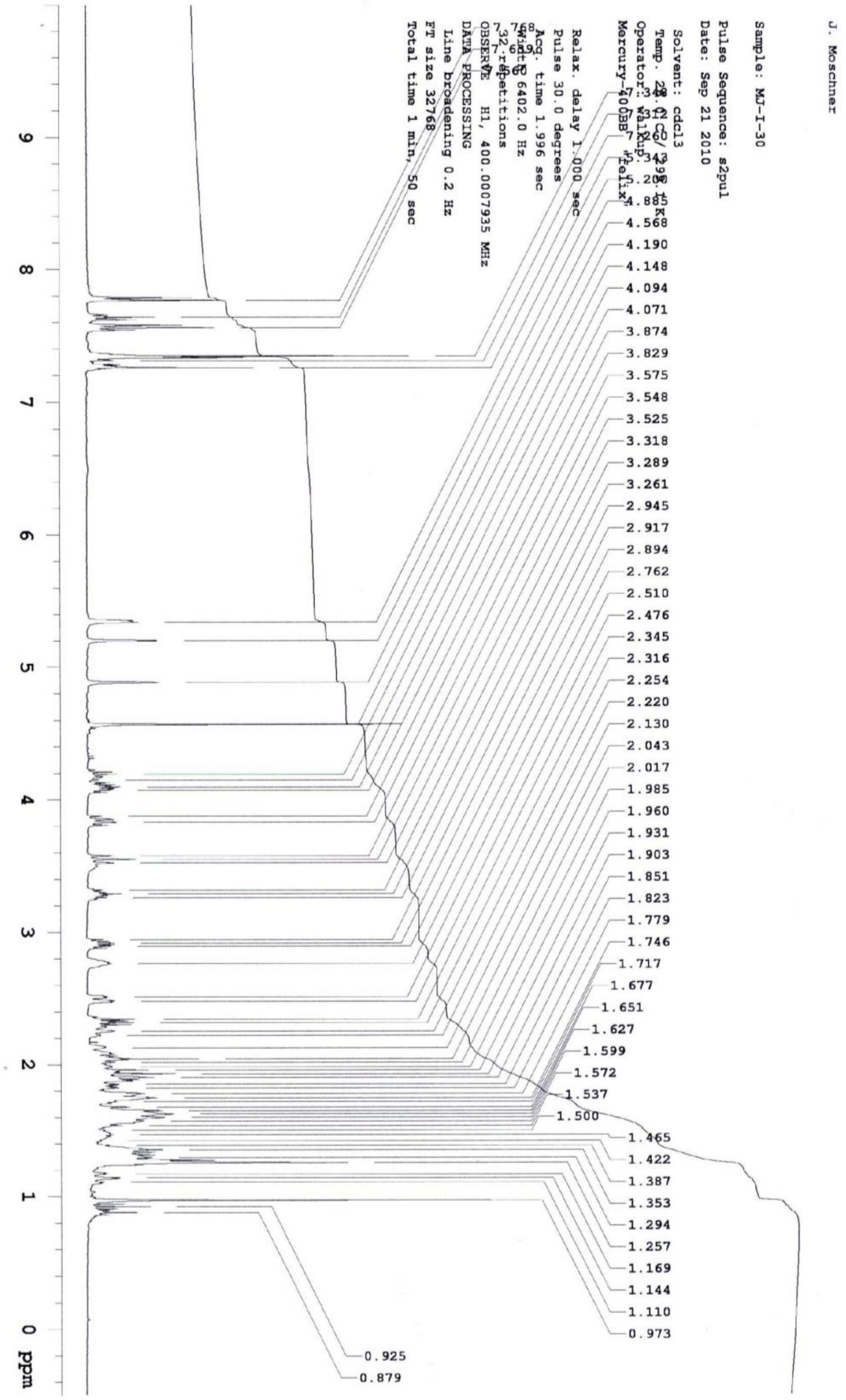
J. Moschner
Sample: MJ-I-30
Pulse Sequence: s2pul
Date: Sep 21 2010
Solvent: cdcl3
Relax. delay 1.000 sec
Pulse 30.0 degrees
Acq. time 1.996 sec
Width 6402.0 Hz
DATA PROCESSING
Line broadening 0.2 Hz
FT size 32768
Total time 1 min, 50 sec
OBSERVE H1, 400.0007935 MHz
4.885
4.568
4.190
4.148
4.094
4.071
3.874
3.829
3.575
3.548
3.525
3.318
3.289
3.261
2.945
2.917
2.894
2.762
2.510
2.476
2.345
2.316
2.254
2.220
2.130
2.043
2.017
1.985
1.960
1.931
1.903
1.851
1.823
1.779
1.746
1.717
1.677
1.651
1.627
1.599
1.572
1.537
1.500
1.465
1.422
1.387
1.353
1.294
1.257
1.169
1.144
1.110
0.973
0.925
0.879
9
8
7
6
5
4
3
2
1
0 ppm

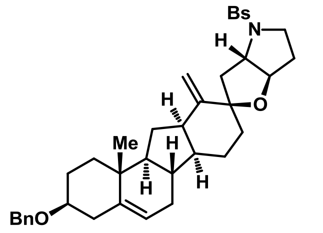
Bs
N
H
H
O
Me
H
H
H
BnO

22

APT (100 MHz, $CDCl_3$)

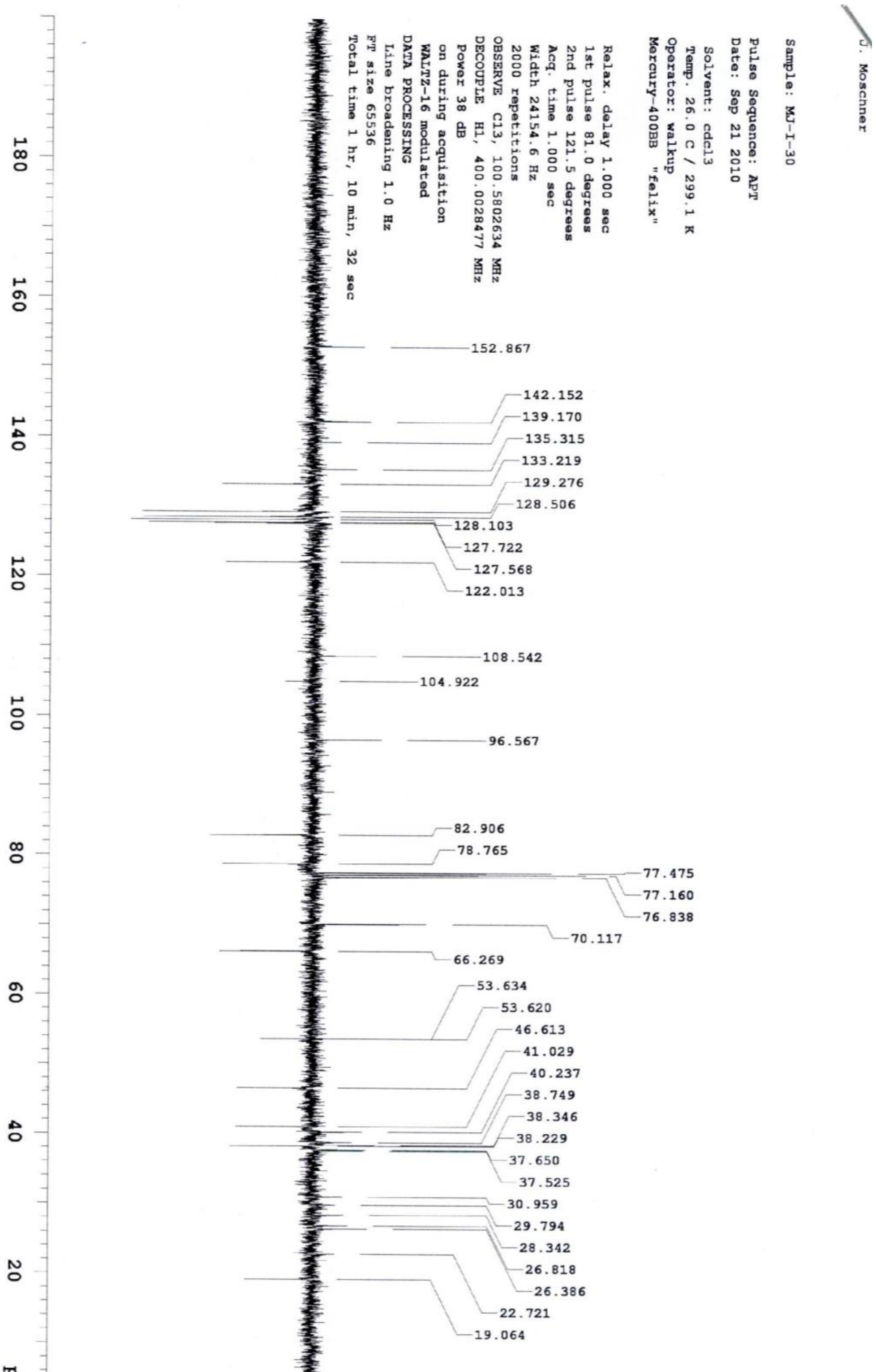

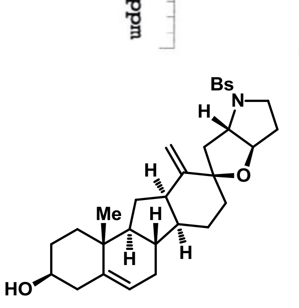

35

^{1}H NMR (400 MHz, $CDCl_3$)

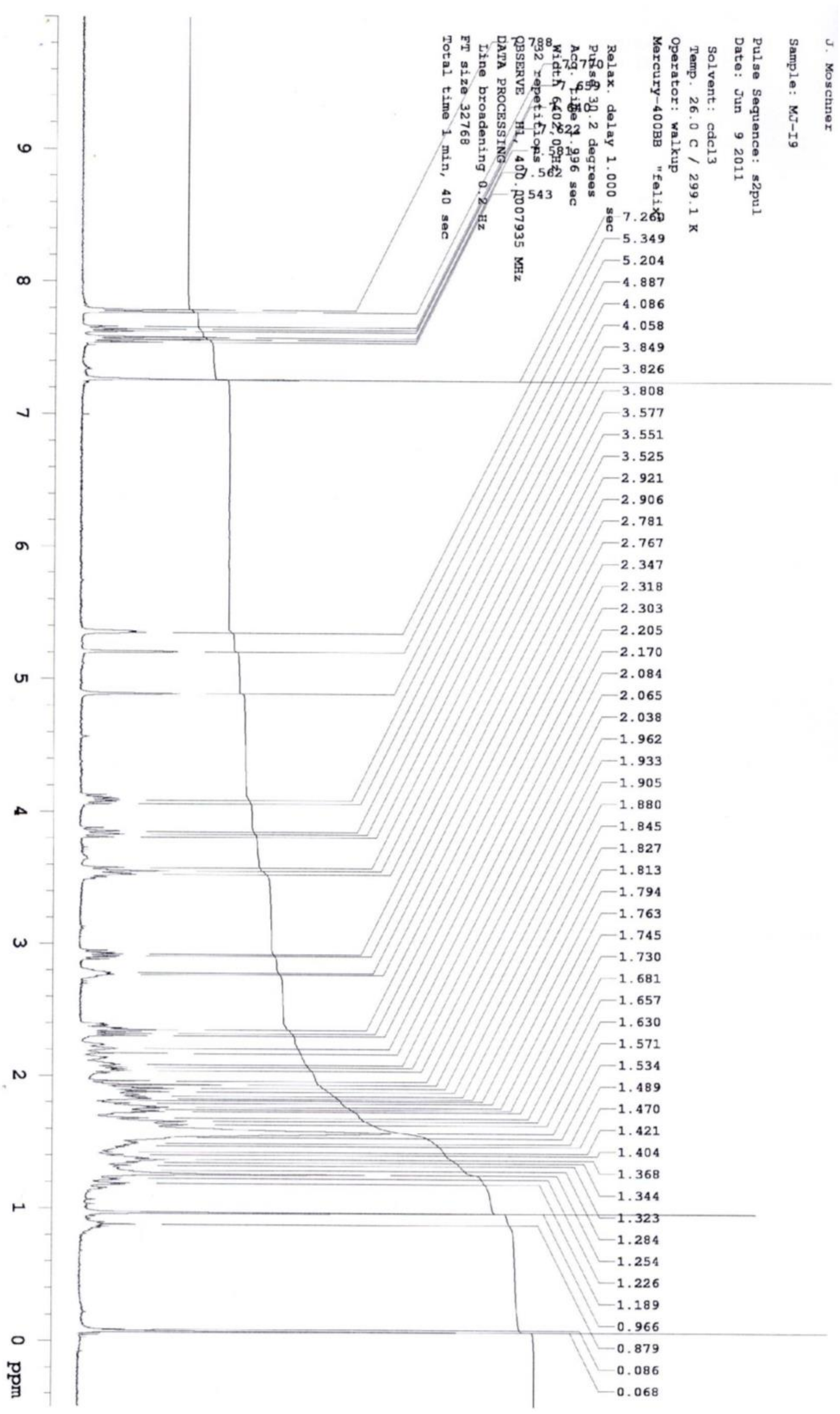

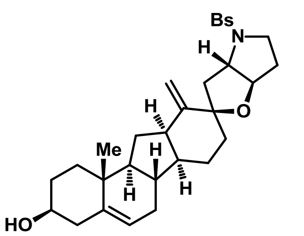

35

^{13}C NMR (100 MHz, $CDCl_3$)

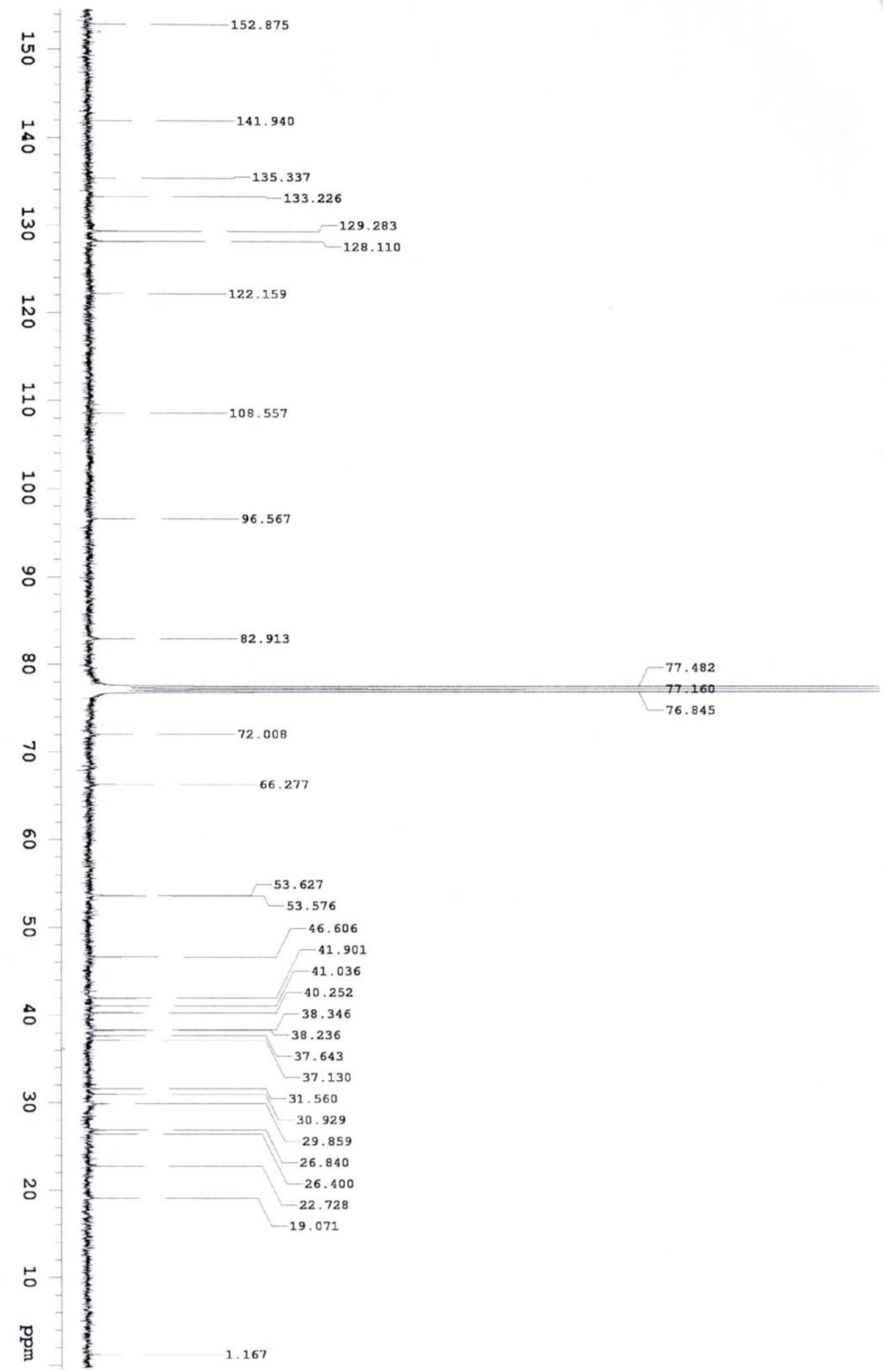

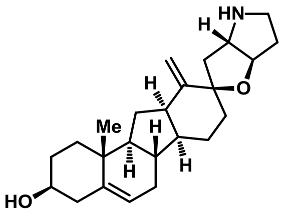

23

^{1}H NMR (400 MHz, $CDCl_3$)

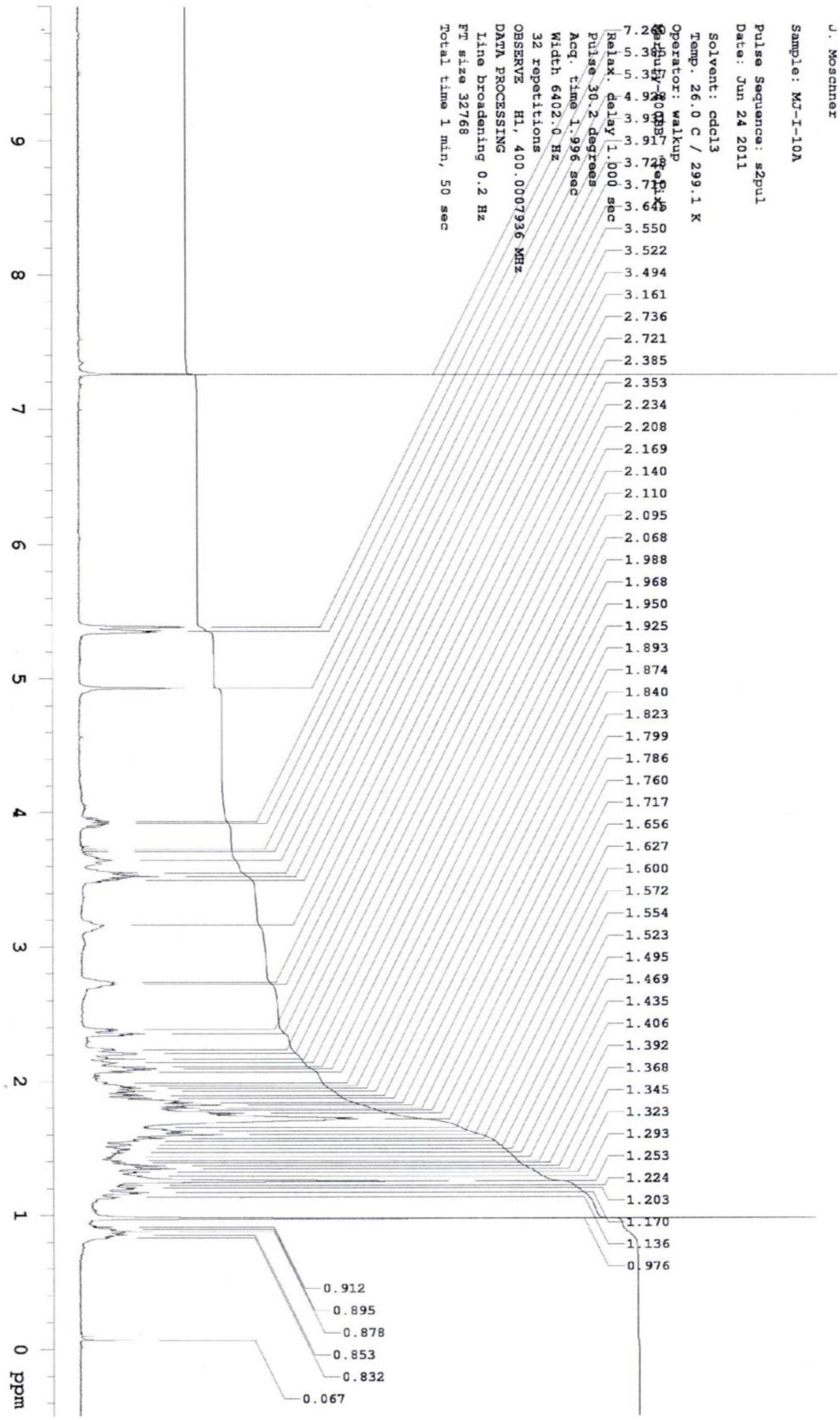
J. Moschner
Sample: MJ-I-10A
Pulse Sequence: s2pul
Date: Jun 24 2011
Solvent: cdcl3
Temp. 26.0 C / 299.1 K
Operator: walkup
Relax. delay 1.000 sec
Pulse 30.2 degrees
Acq. time 1.996 sec
Width 6402.0 Hz
32 repetitions
OBSERVE H1, 400.0007936 MHz
DATA PROCESSING
Line broadening 0.2 Hz
FT size 32768
Total time 1 min, 50 sec
3.917
3.550
3.522
3.494
3.161
2.736
2.721
2.385
2.353
2.234
2.208
2.169
2.140
2.110
2.095
2.068
1.988
1.968
1.950
1.925
1.893
1.874
1.840
1.823
1.799
1.786
1.760
1.717
1.656
1.627
1.600
1.572
1.554
1.523
1.495
1.469
1.435
1.406
1.392
1.368
1.345
1.323
1.293
1.253
1.224
1.203
1.170
1.136
0.976
0.912
0.895
0.878
0.853
0.832
0.067
ppm

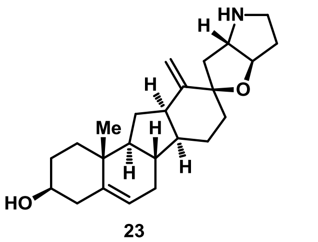
HN
H
H
Me
H
H
O
H
HO

23

^{13}C NMR (100 MHz, $CDCl_3$)

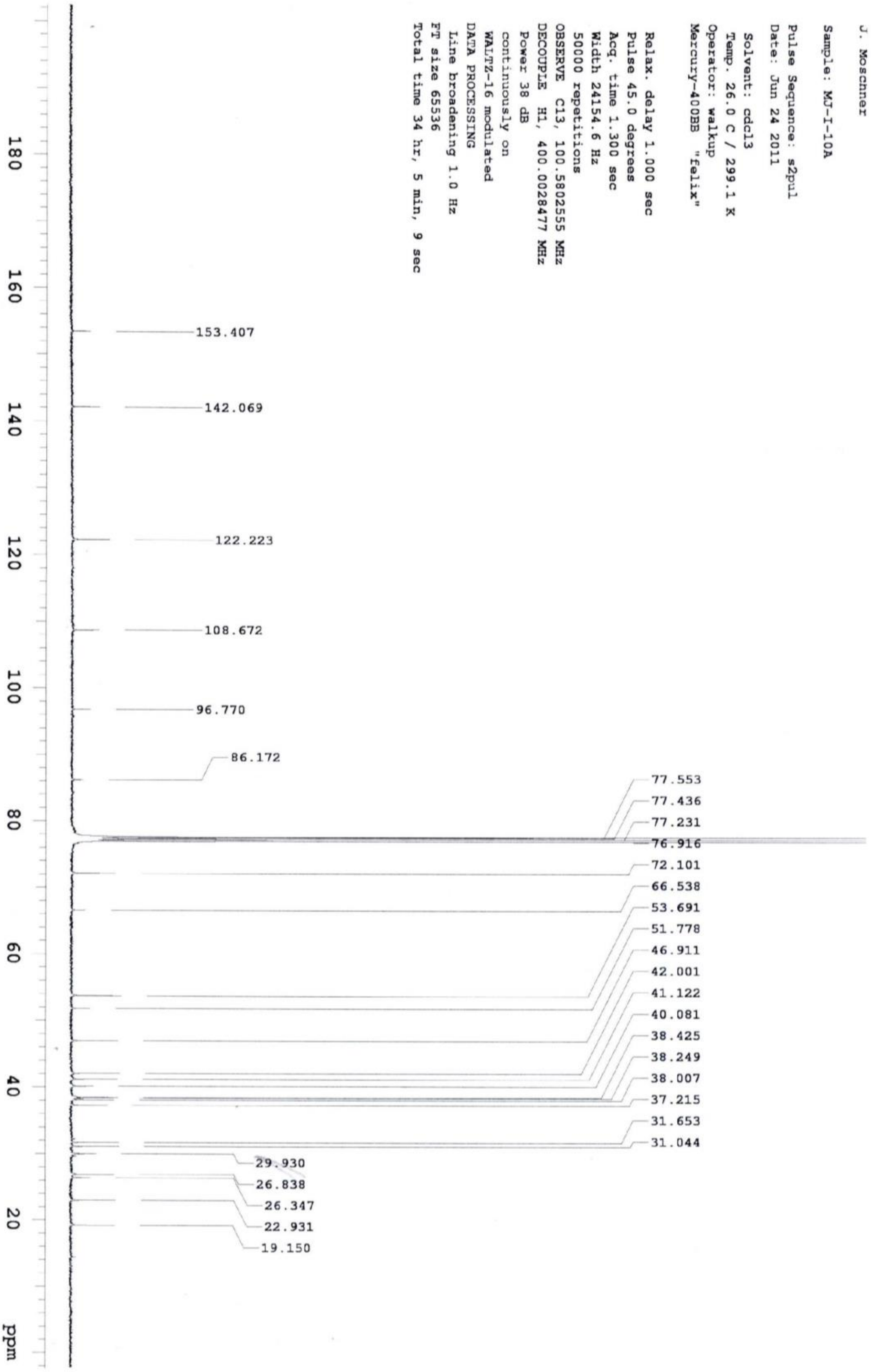

Supplement: File 1 — Experimental details and analytical data of all synthesized compounds are provided. [file Beilstein_J_Org_Chem-09-2328-s001.pdf]
